# Supplementary material for: Altered mRNA transport and local translation in iNeurons with RNA binding protein knockdown
Source: bioRxiv. 2024 Sep 27:2024.09.26.615153. Preprint. [Version 1] doi: 10.1101/2024.09.26.615153 (PMC11463369; doi:10.1101/2024.09.26.615153)
Supplement: Supplement 1 [file media-1.pdf]

Supplemental material for: Altered mRNA transport and local translation in iNeurons with RNA binding protein knockdown

Authors: Rachael Dargan[1], Alla Mikheenko[2], Nicholas L. Johnson[1,3], Benjamin Packer[1], Ziyi Li[1,3], Emma J. Craig[1], Stephanie L. Sarbanes[4], Colleen Bereda[1], Puja R. Mehta[2], Matthew Keuss[2], Mike A. Nalls[1,3], Yue A. Qi[1], Cory A. Weller[1,3], Pietro Fratta[2,5], Veronica H. Ryan[1]

Supplementary figures and legends

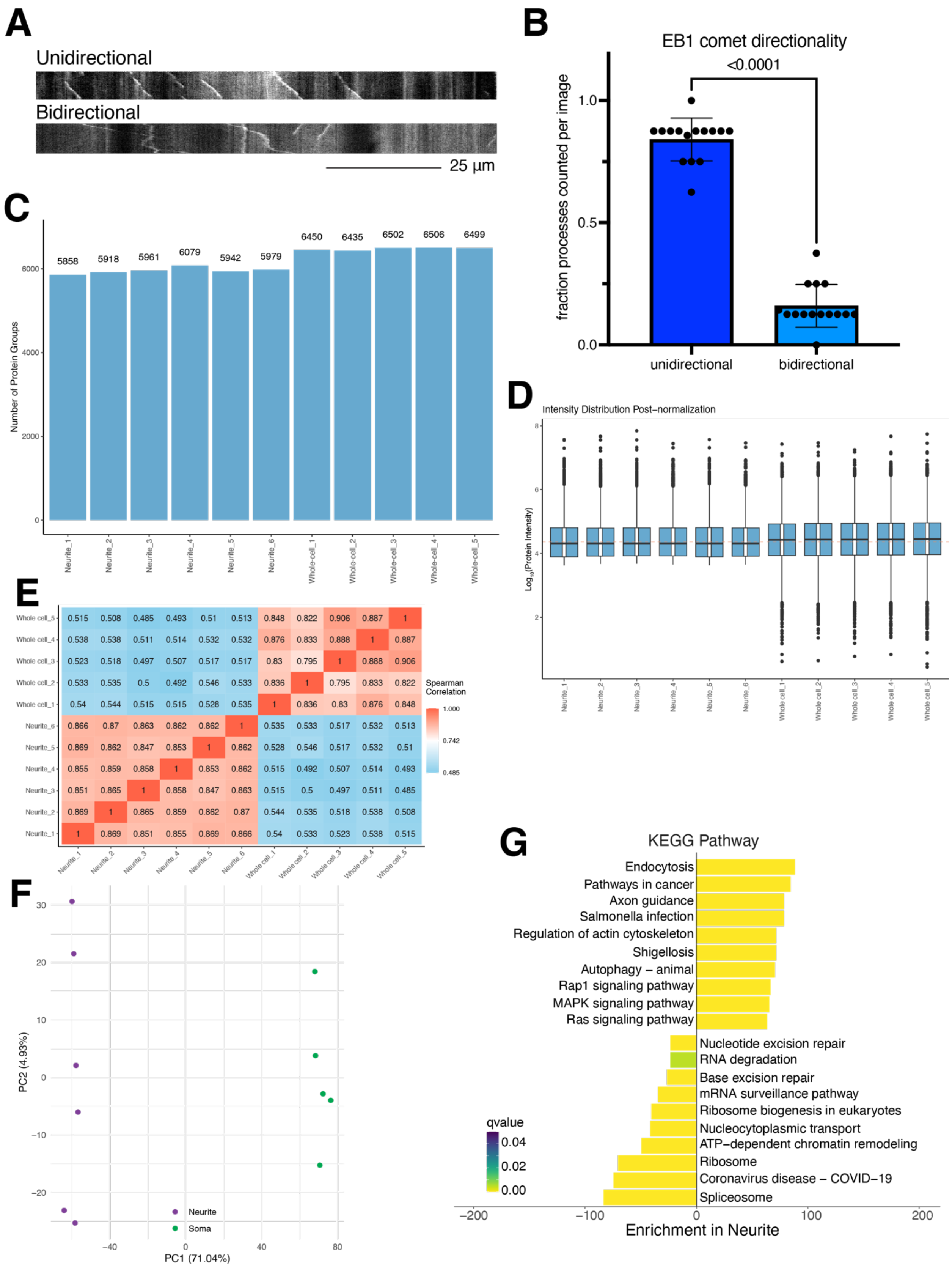

Figure S1: Neurites on underside of membrane are primarily axons. Related to Figure 1.

A. Representative kymographs of EB1 comets from 30 second videos. Processes with unidirectional EB1 comets (top, axons) and bidirectional comets (bottom, dendrites) are shown. Scale bar 25 μm.
B. Quantification of EB1 comet directionality. Neurites from 15 images taken from 2 chambers were quantified as either unidirectional or bidirectional and the fraction of total processes counted per image. 7-8 processes were quantified per image. p-value from two-tailed paired t-test.
C. Number of protein groups identified for each sample; neurite and whole cell samples had a similar number of protein groups.
D. Protein intensity distribution values after normalization for each sample shows similar distributions.
E. Correlation plot of proteomics data shows high correlation between samples from the same cellular fraction.
F. PCA plot of neurite and soma samples shows most of the difference between samples comes from the cellular fraction, not sample-to-sample difference.
G. KEGG analysis of proteins enriched or depleted in neurites shows axon and neurodegeneration-associated terms for proteins enriched in neurites, while proteins enriched in the whole cell fraction are related to nuclear functions (base repair, nucleocytoplasmic transport, splicing).

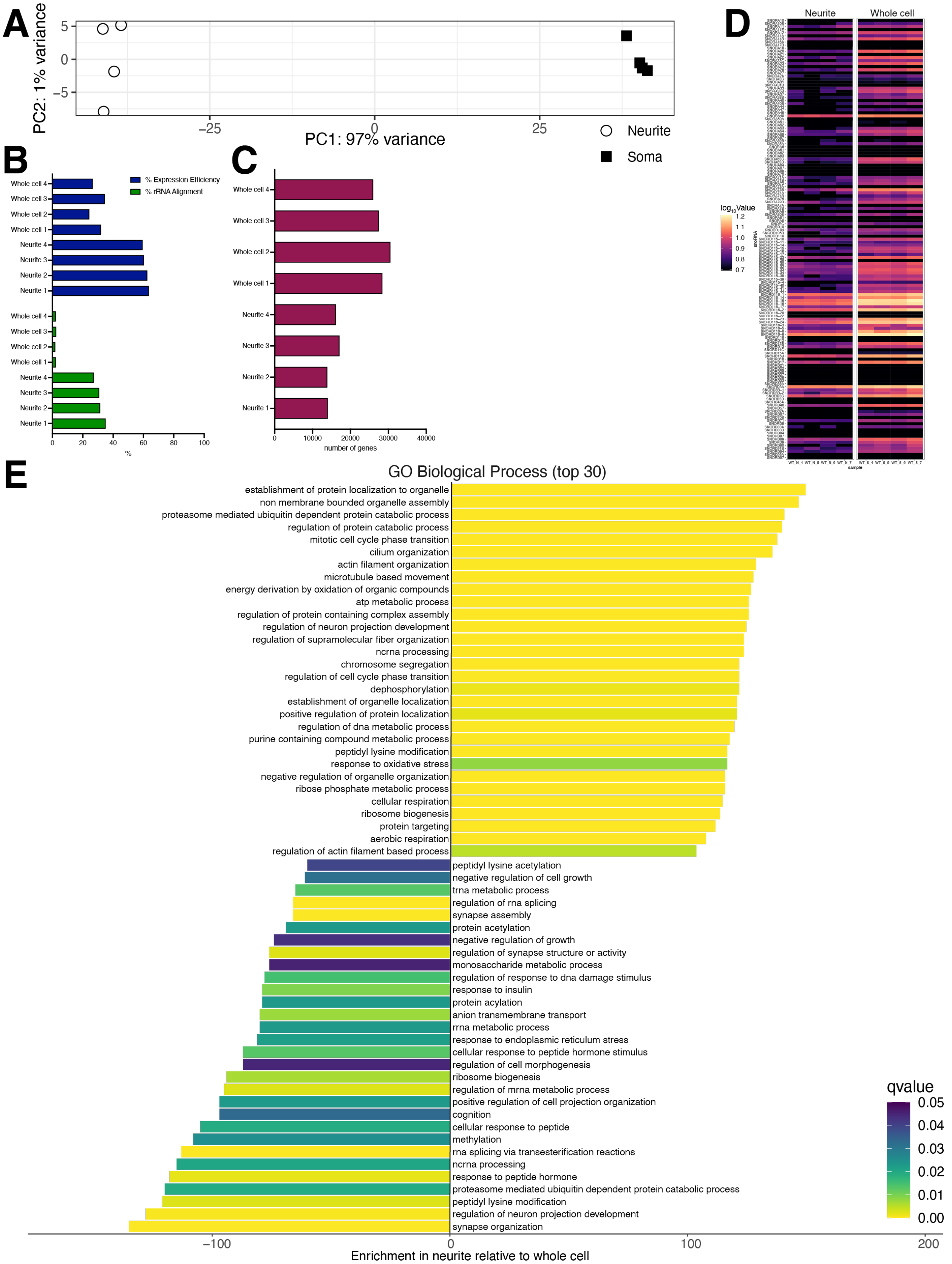
A
PC1: 97% variance
PC2: 1% variance
Neurite
Soma
B
% Expression Efficiency
% rRNA Alignment
Whole cell 4
Whole cell 3
Whole cell 2
Whole cell 1
Neurite 4
Neurite 3
Neurite 2
Neurite 1
%
C
number of genes
D
Neurite
Whole cell
log10Value
snoRNA
sample
E
GO Biological Process (top 30)
establishment of protein localization to organelle
non membrane bounded organelle assembly
proteasome mediated ubiquitin dependent protein catabolic process
regulation of protein catabolic process
mitotic cell cycle phase transition
cilium organization
actin filament organization
microtubule based movement
energy derivation by oxidation of organic compounds
atp metabolic process
regulation of protein containing complex assembly
regulation of neuron projection development
regulation of supramolecular fiber organization
ncrna processing
chromosome segregation
regulation of cell cycle phase transition
dephosphorylation
establishment of organelle localization
positive regulation of protein localization
regulation of dna metabolic process
purine containing compound metabolic process
peptidyl lysine modification
response to oxidative stress
negative regulation of organelle organization
ribose phosphate metabolic process
cellular respiration
ribosome biogenesis
protein targeting
aerobic respiration
regulation of actin filament based process
peptidyl lysine acetylation
negative regulation of cell growth
trna metabolic process
regulation of rna splicing
synapse assembly
protein acetylation
negative regulation of growth
regulation of synapse structure or activity
monosaccharide metabolic process
regulation of response to dna damage stimulus
response to insulin
protein acylation
anion transmembrane transport
rrna metabolic process
response to endoplasmic reticulum stress
cellular response to peptide hormone stimulus
regulation of cell morphogenesis
ribosome biogenesis
regulation of mrna metabolic process
positive regulation of cell projection organization
cognition
cellular response to peptide
methylation
rna splicing via transesterification reactions
ncrna processing
response to peptide hormone
proteasome mediated ubiquitin dependent protein catabolic process
peptidyl lysine modification
regulation of neuron projection development
synapse organization
qvalue
Enrichment in neurite relative to whole cell

Figure S2: Differences between neurite and whole cell fractions by transcriptomics. Related to Figure 2.

A. Principal component analysis of transcriptomics data shows clear separation of neurite fractions from whole cell fractions along a single axis accounting for 97% of the variance between samples.
B. Percent expression efficiency and percent ribosomal RNA alignment for neurite and whole cell samples. rRNA alignment is higher for neurite samples.
C. Number of genes aligned to for neurite and whole cell samples.
D. Heatmap of snoRNA expression values in each sample for neurite and whole cell samples. As expected, whole cell samples have higher amounts of snoRNAs.
E. GO Biological Process terms enriched in neurite (top, positive values) or whole cell (bottom, negative values) show many terms related to ribosomes, transport, and neurons.

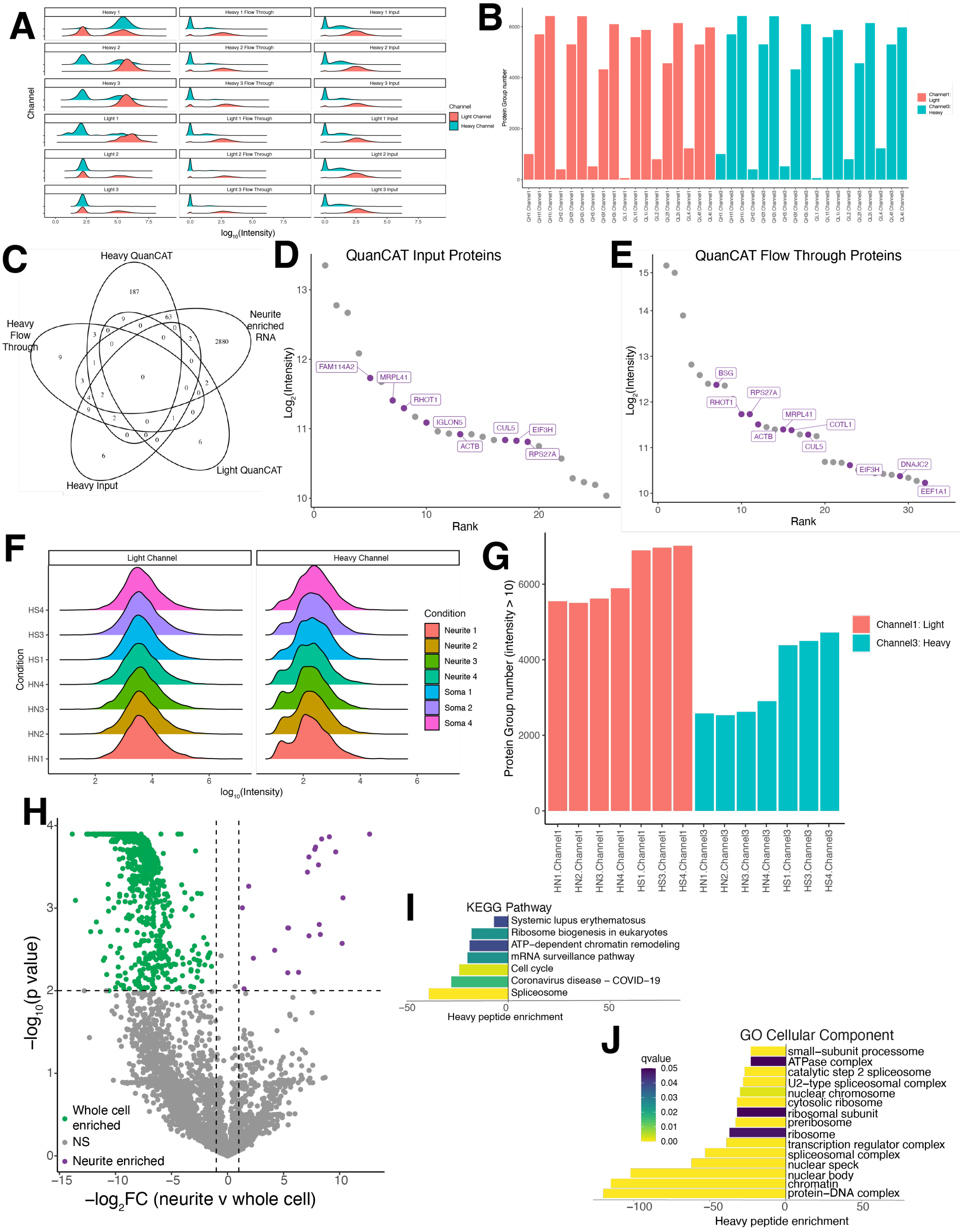

Figure S3: QuanCAT and pSILC quality control. Related to Figure 4.

A. Density plots of heavy and light channels for each QuanCAT sample, including inputs and controls.

B. Number of identified protein groups in both channels for all QuanCAT samples.
C. Venn diagram identifying overlap between the heavy QuanCAT samples, light QuanCAT samples (not exposed to heavy amino acids but treated with AHA), heavy flow through, and heavy input samples, as well as the neurite enriched RNAs. 8 proteins overlapped between the heavy QuanCAT and heavy flow through samples, while 4 overlapped between the input and heavy QuanCAT, demonstrating the specificity of the AHA isolation.
D. Rank plot of heavy proteins identified in the input fractions shows very few proteins, with some overlaps with the transcripts enriched in neurites (purple).
E. Rank plot of heavy proteins identified in the flow through fractions shows very few proteins, with some overlaps with the transcripts enriched in neurites (purple).
F. Density plots of heavy and light channels for each pSILAC sample.
G. Number of identified protein groups in both channels for all pSILAC experiments.
H. Volcano plot of neurite (purple) and soma (green) enriched newly translated proteins. More proteins are translated in the neuritic fraction than the soma fraction. n=4 neurite samples, n=3 whole cell samples.
I. KEGG pathway analysis of proteins containing heavy amino acids in neurites versus whole cell shows many pathways related to nuclear functions, like ribosome biogenesis and chromatin remodeling.
J. Cell component GO analysis of proteins containing heavy amino acids in neurites versus whole cell shows many nucleus-related terms.

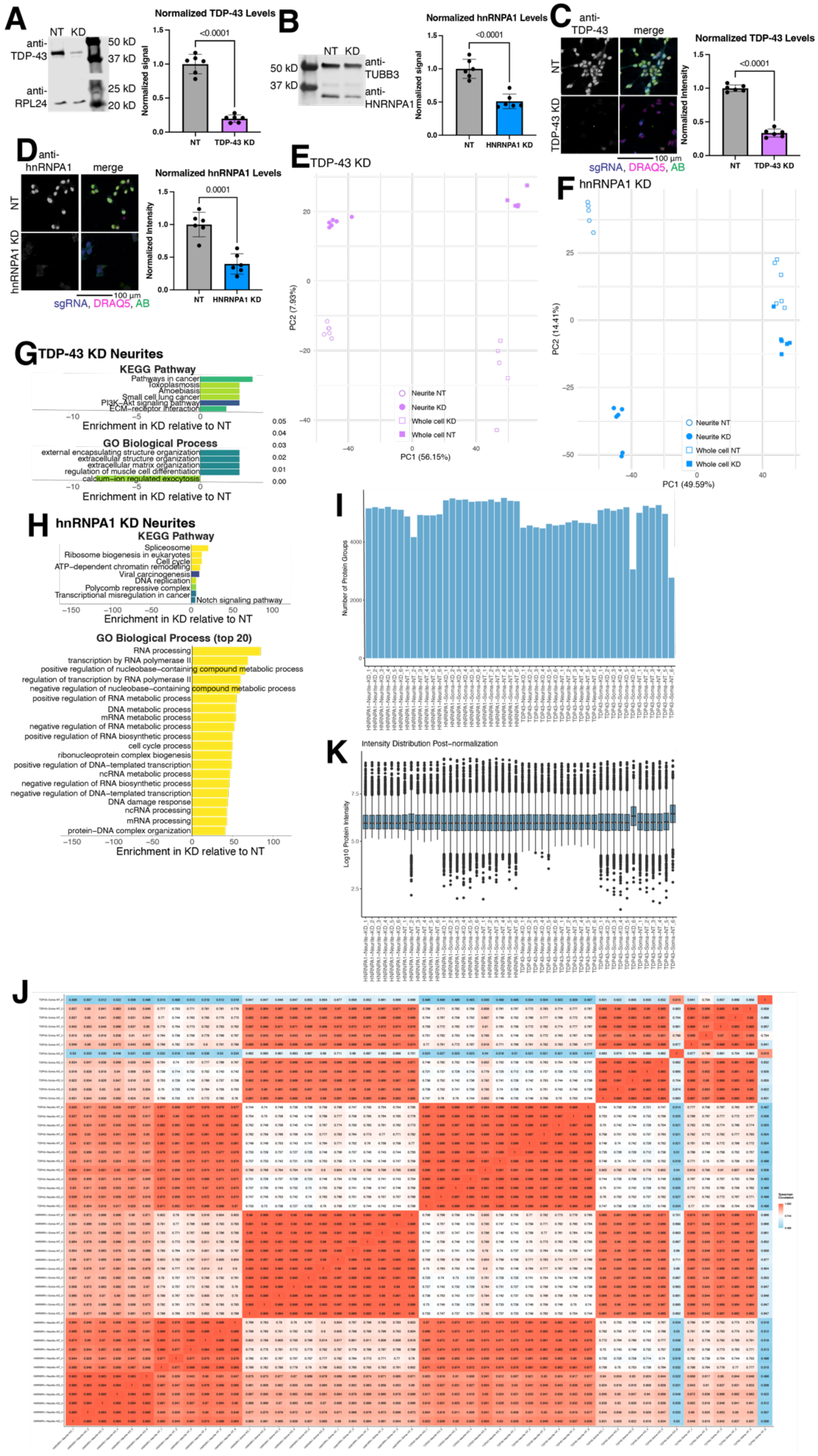
A
NT KD
anti-TDP-43
anti-RPL24
50 kD
37 kD
25 kD
20 kD
Normalized TDP-43 Levels
Normalized signal
<0.0001
NT
TDP-43 KD
B
NT KD
50 kD
37 kD
anti-TUBB3
anti-HNRNPA1
Normalized hnRNPA1 Levels
Normalized signal
<0.0001
NT
HNRNPA1 KD
C
anti-TDP-43
merge
NT
TDP-43 KD
100 µm
sgRNA, DRAQ5, AB
Normalized TDP-43 Levels
Normalized Intensity
<0.0001
NT
TDP-43 KD
D
anti-hnRNPA1
merge
NT
hnRNPA1 KD
100 µm
sgRNA, DRAQ5, AB
Normalized hnRNPA1 Levels
Normalized Intensity
0.0001
NT
HNRNPA1 KD
E
TDP-43 KD
PC2 (7.93%)
PC1 (56.15%)
Neurite NT
Neurite KD
Whole cell KD
Whole cell NT
F
hnRNPA1 KD
PC2 (14.41%)
PC1 (49.59%)
Neurite NT
Neurite KD
Whole cell NT
Whole cell KD
G
TDP-43 KD Neurites
KEGG Pathway
Pathways in cancer
Toxoplasmosis
Amoebiasis
Small cell lung cancer
PI3K–Akt signaling pathway
ECM–receptor interaction
Enrichment in KD relative to NT
GO Biological Process
external encapsulating structure organization
extracellular structure organization
extracellular matrix organization
regulation of muscle cell differentiation
calcium–ion regulated exocytosis
Enrichment in KD relative to NT
H
hnRNPA1 KD Neurites
KEGG Pathway
Spliceosome
Ribosome biogenesis in eukaryotes
Cell cycle
ATP–dependent chromatin remodeling
Viral carcinogenesis
DNA replication
Polycomb repressive complex
Transcriptional misregulation in cancer
Notch signaling pathway
Enrichment in KD relative to NT
GO Biological Process (top 20)
RNA processing
transcription by RNA polymerase II
positive regulation of nucleobase–containing compound metabolic process
regulation of transcription by RNA polymerase II
negative regulation of nucleobase–containing compound metabolic process
positive regulation of RNA metabolic process
DNA metabolic process
mRNA metabolic process
negative regulation of RNA metabolic process
positive regulation of RNA biosynthetic process
cell cycle process
ribonucleoprotein complex biogenesis
positive regulation of DNA–templated transcription
ncRNA metabolic process
negative regulation of RNA biosynthetic process
negative regulation of DNA–templated transcription
DNA damage response
ncRNA processing
mRNA processing
protein–DNA complex organization
Enrichment in KD relative to NT
I
Number of Protein Groups
K
Intensity Distribution Post–normalization
Log10 Protein Intensity
J

Figure S4: sgRNAs effectively knock down target proteins. Related to Figure 5.

A-B. Western blot validation of RBP KD for (A) TDP-43 and (B) hnRNPA1. Representative western blot is shown on the left, with quantification to the right. P-values from two-tailed unpaired t-test.

C-D. Immunofluorescence validation of RBP KD for (C) TDP-43 and (D) hnRNPA1. Representative images are shown on the left, with quantification to the right. IF shows similar levels of KD to western blots in A-B. P-values from two-tailed unpaired t-test.

E-F. PCA plots of proteomics experiments for (E) TDP-43 KD and (F) hnRNPA1 KD show the majority of variance (48-60%, PC1) between samples is due to the neurite to whole cell difference rather than KDs (<20%, PC2).

G. KEGG and GO BP analysis comparing TDP-43 KD to NT neurites shows various enriched pathways, including extracellular matrix organization.

H. KEGG and GO BP analysis comparing hnRNPA1 KD to NT neurites shows various enriched pathways, including many RNA and DNA metabolism related terms.

I. Number of non-zero proteins for each sample, used to identify samples with few proteins detected. Two samples, TDP-43 KD soma 6 and TDP-43 NT soma 6, had low protein counts and were excluded from the analysis.

J. Correlation plot of all samples identifies 3 samples (TDP43-Soma-NT_6, TDP43-Soma-KD_6, HNRNPA1-Neurite-NT_2) that are not well correlated with other samples of the same type and thus were excluded from the analysis.

K. Graph of peptide intensity distribution for each sample after normalization. Only 2 samples (TDP-43 KD soma 6 and TDP-43 NT soma 6) have different intensity distributions and were removed from the analysis.

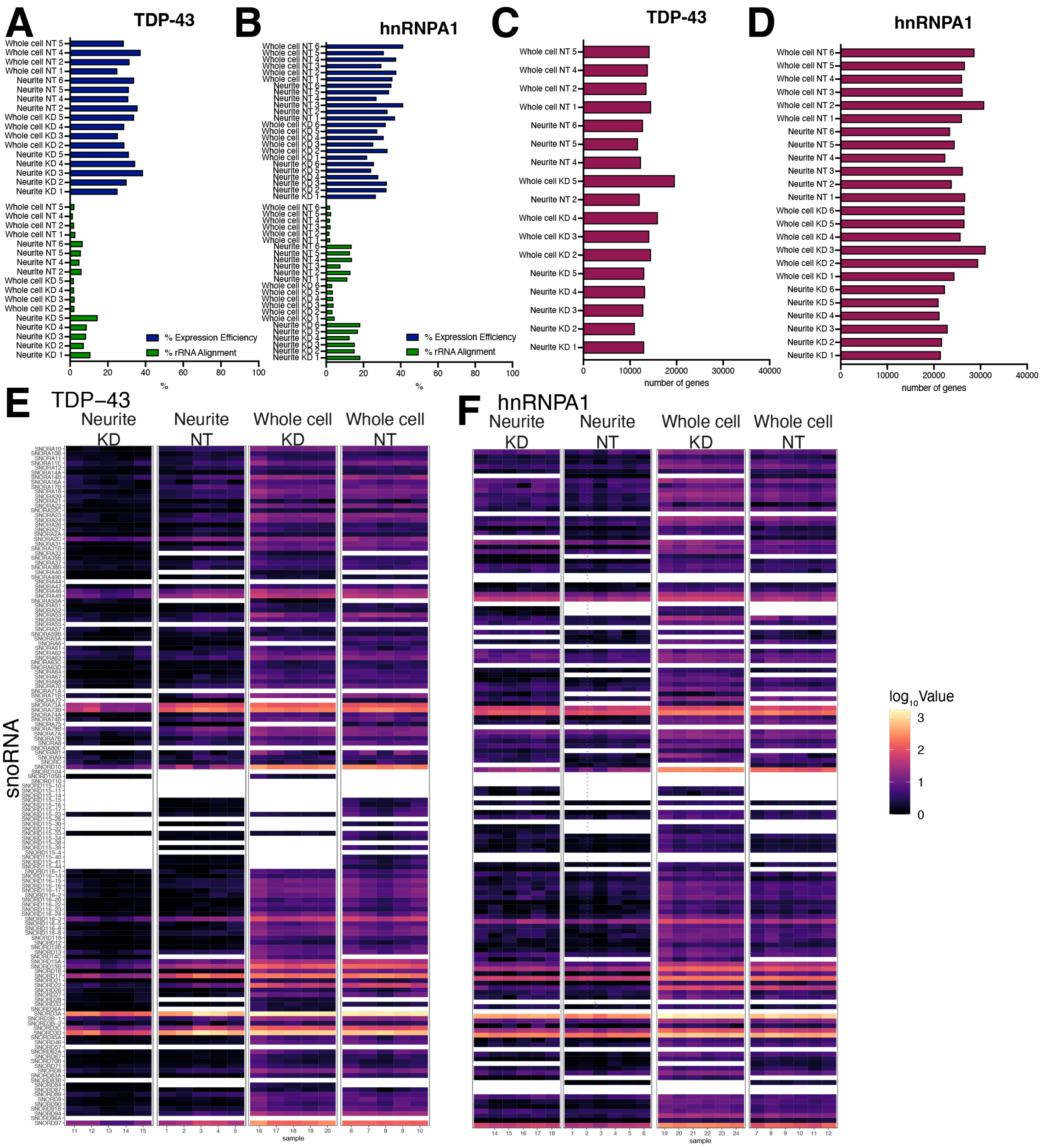

Figure S5: Quality control of RNA sequencing. Related to Figure 6.

A-B: Percent expression efficiency and percent ribosomal RNA alignment for (A) TDP-43 and (B) hnRNPA1 KD samples. rRNA alignment is higher for neurite samples.

C-D: Number of genes aligned to for (C) TDP-43 and (D) hnRNPA1 KD samples. hnRNPA1 samples align to the most genes.

E-F: Heatmap of snoRNA expression values in each sample for (E) TDP-43 and (F) hnRNPA1 KD samples. As expected, whole cell samples have higher amounts of snoRNAs.

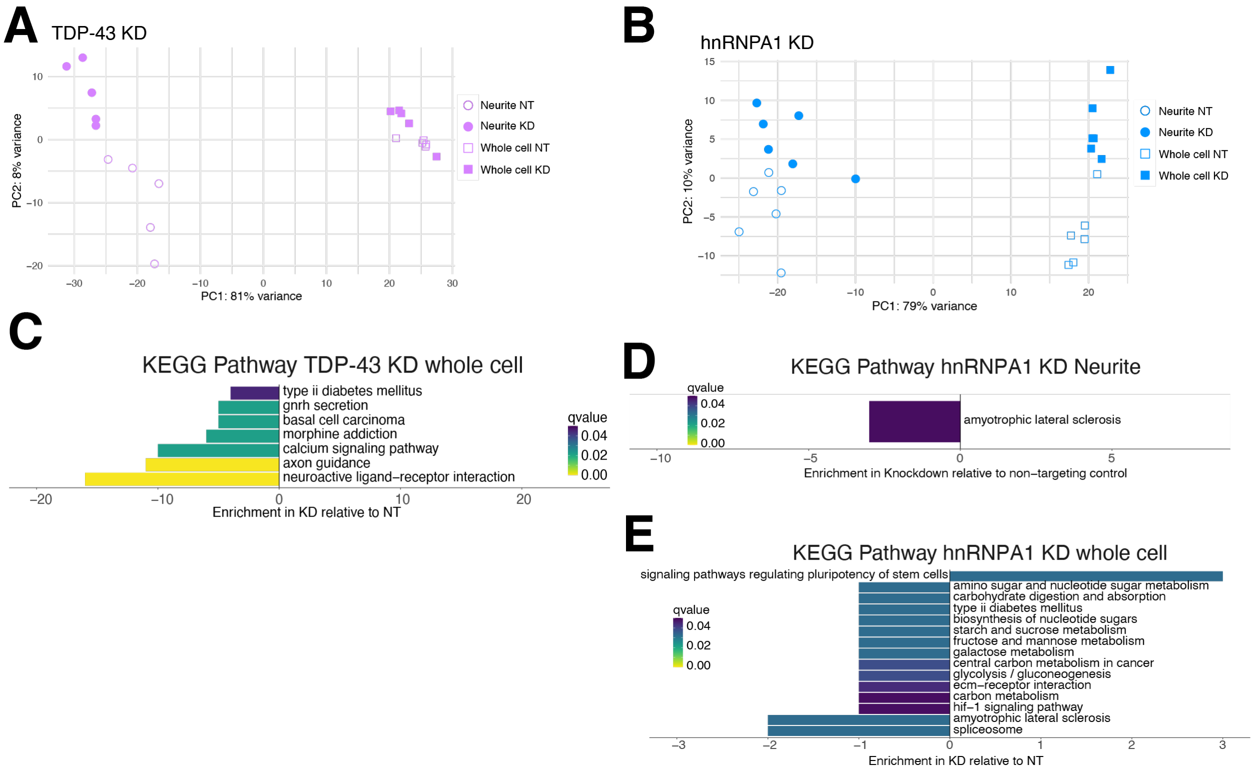

Figure S6: Neurite versus whole cell dominates difference between guides in transcriptomics. Related to Figure 6.

A. PCA plot of TDP-43 KD neurite and whole cell RNA seq shows that most of the difference (81%) between the samples comes from the cell compartment, while a minority (8%) can be explained by the difference between the KD and NT guides.
B. PCA plot of hnRNPA1 KD neurite and whole cell RNA seq shows that most of the difference (79%) between the samples comes from the cell compartment, while a minority (10%) can be explained by the difference between the KD and NT guides.
C. KEGG pathway analysis of transcripts changed in the whole cell in TDP-43 KD versus NT neurons. Axon guidance and neuroactive ligand-receptor interaction are decreased. No KEGG terms were significantly enriched in neurites.
D. KEGG pathway analysis of transcripts changed in hnRNPA1 KD versus NT neurites. The only significant term is a decrease in the ALS term.
E. KEGG pathway analysis of transcripts changed in the whole cell in hnRNPA1 KD versus NT neurons. Sugar metabolism and ALS terms are decreased.

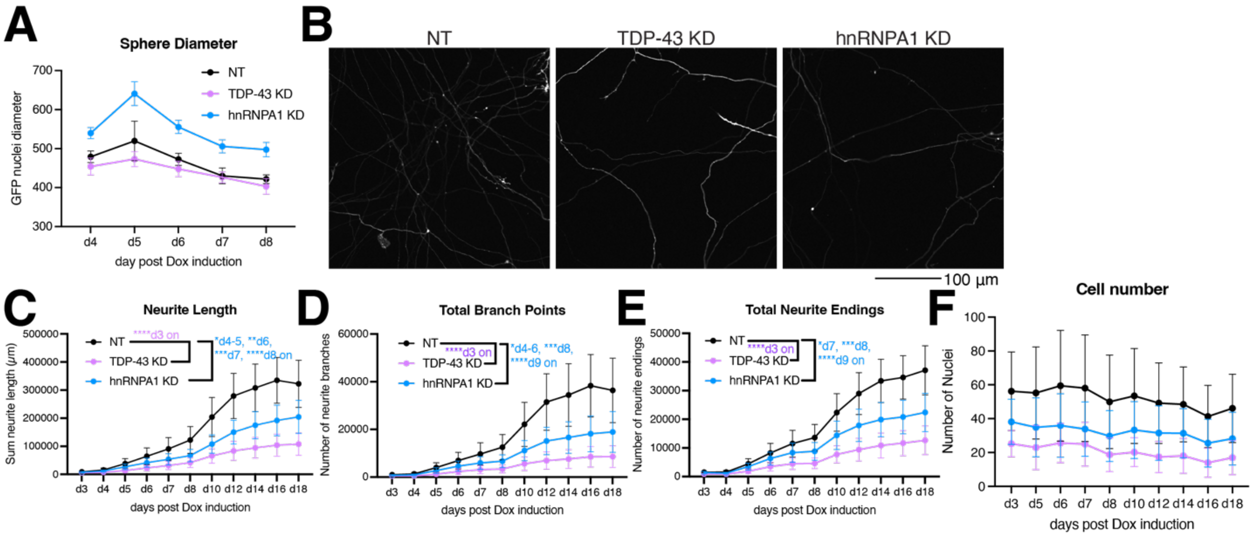

Figure S7: 2D neurite outgrowth shows similar neurite outgrowth defects to neurospheres. Related to Figure 7.

A. Plot of nucleus diameter of spheres in Figure 7D-E. hnRNPA1 KD spheres are slightly larger (have more cells), but NT and TDP-43 KD all have similar sizes. There is no significant change in the area taken up by the cell bodies day to day.
B. Representative images of 2D neurite outgrowth experiment. Image is cytosolic mScarlet channel. n=24 wells per genotype, scale bar 100 µm.
C. C-F: Quantification of 2D neurite outgrowth and neuron survival longitudinal imaging. TDP-43 and hnRNPA1 KD have decreased total neurite length (D), total branch points (E), and number of neurite endings (F). However, there is no significant change in cell counts over the course of the experiment (G). P-values from two-way ANOVA with Dunnett's multiple comparison test.

Supplemental Tables

Table S1: Identified cryptic exons in TDP-43 KD neurons.

Table S2: Identified cryptic exons in hnRNPA1 KD neurons.

Table S1: Identified cryptic exons in TDP-43 KD neurons.

	module_id	gene_id	gene_name	seqid	strand	lsv_id	event_id	complex	module_ever	denovo	junction_nan	junctions_co	event_non_cl	event_chang	junction_cha	refPSI	KDSoma_me	deltaPSI	prob	padjust	event	abs(deltaPSI)
1	ENSG000001	ENSG000001	NUP210	chr3	-		ENSG000001	TRUE	tandem_cass	TRUE	Distal	chr3:133660	FALSE	FALSE	TRUE	0.014	0.304	0.279	1	0	AFE/ALE	0.279
2	ENSG000001	ENSG000001	MICAL1	chr6	-		ENSG000001	TRUE	cassette^6|t	TRUE	Distal	chr6:109447	FALSE	FALSE	TRUE	0.007	0.138	0.111	0.976	0.012	AFE/ALE	0.111
3	ENSG000001	ENSG000001	UBC	chr12	-		ENSG000001	TRUE	putative_alt3	TRUE	Proximal	chr12:12491	FALSE	FALSE	FALSE	0.047	0.154	0.12	0.932	0.03	AFE/ALE	0.12
4	ENSG000001	ENSG000001	UBC	chr12	-		ENSG000001	TRUE	putative_alt3	TRUE	Distal	chr12:12491	FALSE	FALSE	TRUE	0.003	0.182	0.157	1	0	AFE/ALE	0.157
5	ENSG00000	ENSG00000	KLHL13	chrX	-	ENSG00000	ENSG00000	TRUE	cassette^1|a	TRUE	Distal	chrX:117914	FALSE	TRUE	TRUE	0.004	0.221	0.198	0.997	0.001	AFE/ALE	0.198
6	ENSG00000	ENSG00000	SPPL2B	chr19	+	ENSG00000	ENSG00000	TRUE	cassette^1|i	FALSE	C2_A	chr19:23373	FALSE	FALSE	TRUE	0.041	0.244	0.205	1	0	cassette	0.205
7	ENSG00000	ENSG00000	TSPOAP1	chr17	-	ENSG00000	ENSG00000	TRUE	alt5^1|ir^2	TRUE	Proximal	chr17:58305	FALSE	FALSE	TRUE	0.01	0.16	0.135	0.995	0.002	AFE/ALE	0.135
8	ENSG00000	ENSG00000	ITGA3	chr17	+	ENSG00000	ENSG00000	FALSE	cassette^1	FALSE	C1_A	chr17:50080	FALSE	FALSE	TRUE	0.043	0.301	0.258	1	0	cassette	0.258
9	ENSG00000	ENSG00000	IFFO1	chr12	-	ENSG00000	ENSG00000	FALSE	cassette^1	FALSE	C1_A	chr12:65494	FALSE	FALSE	FALSE	0.037	0.165	0.128	0.91	0.028	cassette	0.128
10	ENSG00000	ENSG00000	SYT7	chr11	-	ENSG00000	ENSG00000	TRUE	cassette^4|t	TRUE	C1_A	chr11:61547	FALSE	TRUE	TRUE	0	0.552	0.539	1	0	cassette	0.539
11	ENSG00000	ENSG00000	MNAT1	chr14	+	ENSG00000	ENSG00000	TRUE	cassette^1|i	TRUE	C1_A	chr14:60812	FALSE	TRUE	TRUE	0.047	0.673	0.633	1	0	cassette	0.633
12	ENSG00000	ENSG00000	GRAMD1B	chr11	+	ENSG00000	ENSG00000	TRUE	cassette^2|r	FALSE	C1_A	chr11:12357	FALSE	FALSE	TRUE	0.031	0.149	0.119	0.961	0.018	cassette	0.119
13	ENSG00000	ENSG00000	POU2F2	chr19	-	ENSG00000	ENSG00000	FALSE	putative_afe	TRUE	Proximal	chr19:42122	FALSE	TRUE	TRUE	0.002	0.344	0.32	1	0	AFE/ALE	0.32
14	ENSG00000	ENSG00000	BRD9	chr5	-	ENSG00000	ENSG00000	TRUE	cassette^1|i	FALSE	C1_C2	chr5:879889	FALSE	FALSE	FALSE	0.002	0.143	-0.113	0.938	0.029	skipping	0.113
15	ENSG00000	ENSG00000	SYNE2	chr14	+	ENSG00000	ENSG00000	FALSE	putative_afe	TRUE	Proximal	chr14:63853	FALSE	FALSE	FALSE	0.039	0.164	0.123	0.932	0.027	AFE/ALE	0.123
16	ENSG00000	ENSG00000	USP36	chr17	-	ENSG00000	ENSG00000	TRUE	ir^2|putative	TRUE	Proximal	chr17:78836	FALSE	FALSE	TRUE	0.019	0.195	0.163	0.996	0.001	AFE/ALE	0.163
17	ENSG00000	ENSG00000	USP13	chr3	+	ENSG00000	ENSG00000	FALSE	alt5^1	FALSE	Proximal	chr3:179701	FALSE	TRUE	TRUE	0.05	0.651	0.62	1	0	AFE/ALE	0.62
18	ENSG00000	ENSG00000	CAMK2B	chr7	-	ENSG00000	ENSG00000	TRUE	cassette^4|t	TRUE	C1_A	chr7:442585	FALSE	FALSE	TRUE	0.001	0.311	0.289	1	0	cassette	0.289
19	ENSG00000	ENSG00000	CAMK2B	chr7	-	ENSG00000	ENSG00000	TRUE	cassette^4|t	TRUE	C1_A	chr7:442586	FALSE	FALSE	TRUE	0.002	0.227	0.204	1	0	cassette	0.204
20	ENSG00000	ENSG00000	CAMK2B	chr7	-	ENSG00000	ENSG00000	TRUE	ir^3|ale^1|a	TRUE	Proximal	chr7:442209	FALSE	TRUE	TRUE	0	0.395	0.378	1	0	AFE/ALE	0.378
21	ENSG00000	ENSG00000	CAMK2B	chr7	-	ENSG00000	ENSG00000	TRUE	cassette^4|t	TRUE	Distal	chr7:442546	FALSE	FALSE	TRUE	0.004	0.5	0.48	1	0	AFE/ALE	0.48
22	ENSG00000	ENSG00000	PSD	chr10	-	ENSG00000	ENSG00000	TRUE	cassette^3|t	TRUE	C1_A	chr10:10241	FALSE	FALSE	TRUE	0	0.408	0.394	1	0	cassette	0.394
23	ENSG00000	ENSG00000	PSD	chr10	-	ENSG00000	ENSG00000	TRUE	cassette^3|t	TRUE	C2_A_Last	chr10:10241	FALSE	FALSE	TRUE	0	0.297	0.276	1	0	cassette	0.276
24	ENSG00000	ENSG00000	CDON	chr11	-	ENSG00000	ENSG00000	FALSE	alt3^1	TRUE	Distal	chr11:12598	FALSE	TRUE	TRUE	0.002	0.332	0.314	1	0	AFE/ALE	0.314
25	ENSG00000	ENSG00000	ZFAT	chr8	-	ENSG00000	ENSG00000	FALSE	putative_afe	TRUE	Proximal	chr8:134637	FALSE	TRUE	TRUE	0.006	0.282	0.243	1	0	AFE/ALE	0.243
26	ENSG00000	ENSG00000	PFKP	chr10	+	ENSG00000	ENSG00000	TRUE	cassette^2|a	TRUE	C1_A	chr10:30993	FALSE	TRUE	TRUE	0.012	0.689	0.685	1	0	cassette	0.685
27	ENSG00000	ENSG00000	PFKP	chr10	+	ENSG00000	ENSG00000	TRUE	cassette^2|a	TRUE	C1_A	chr10:30993	FALSE	FALSE	FALSE	0.001	0.142	0.112	0.94	0.029	cassette	0.112
28	ENSG00000	ENSG00000	ADGRL1	chr19	-	ENSG00000	ENSG00000	TRUE	cassette^1|i	TRUE	C1_A	chr19:14176	FALSE	TRUE	TRUE	0	0.256	0.234	1	0	cassette	0.234
29	ENSG00000	ENSG00000	ADGRL1	chr19	-	ENSG00000	ENSG00000	TRUE	cassette^1|i	TRUE	Distal	chr19:14170	FALSE	FALSE	TRUE	0	0.217	0.198	1	0	AFE/ALE	0.198
30	ENSG00000	ENSG00000	SPEG	chr2	+	ENSG00000	ENSG00000	TRUE	ir^3|ale^2|p	TRUE	Proximal	chr2:219465	FALSE	FALSE	TRUE	0.014	0.326	0.309	1	0	AFE/ALE	0.309
31	ENSG00000	ENSG00000	KCNQ2	chr20	-	ENSG00000	ENSG00000	TRUE	cassette^1|i	TRUE	C1_C2	chr20:63439	FALSE	TRUE	TRUE	0.028	0.261	-0.242	1	0	skipping	0.242
32	ENSG00000	ENSG00000	KCNQ2	chr20	-	ENSG00000	ENSG00000	TRUE	cassette^1|i	TRUE	Distal	chr20:63439	FALSE	TRUE	TRUE	0.039	0.256	0.229	1	0	AFE/ALE	0.229
33	ENSG00000	ENSG00000	CTTNBP2	chr7	-	ENSG00000	ENSG00000	TRUE	cassette^1|i	TRUE	Proximal	chr7:117861	FALSE	FALSE	TRUE	0.013	0.168	0.145	0.999	0	AFE/ALE	0.145
34	ENSG00000	ENSG00000	ACTL6B	chr7	-	ENSG00000	ENSG00000	TRUE	cassette^1|i	TRUE	C1_A	chr7:100650	FALSE	TRUE	TRUE	0.021	0.636	0.626	1	0	cassette	0.626
35	ENSG00000	ENSG00000	PIAS2	chr18	-	ENSG00000	ENSG00000	FALSE	cassette^1	FALSE	C2_C1	chr18:46855	FALSE	FALSE	FALSE	0.033	0.159	-0.125	0.911	0.03	skipping	0.125
36	ENSG00000	ENSG00000	UIMC1	chr5	-	ENSG00000	ENSG00000	FALSE	cassette^1	TRUE	C1_A	chr5:176980	FALSE	FALSE	TRUE	0.021	0.123	0.1	0.957	0.022	cassette	0.1
37	ENSG00000	ENSG00000	PHACTR3	chr20	+	ENSG00000	ENSG00000	TRUE	putative_afe	TRUE	Proximal	chr20:59742	FALSE	FALSE	FALSE	0.001	0.131	0.103	0.939	0.031	AFE/ALE	0.103
38	ENSG00000	ENSG00000	CRLS1	chr20	+	ENSG00000	ENSG00000	TRUE	cassette^2|i	TRUE	C1_A	chr20:60154	FALSE	TRUE	TRUE	0.002	0.229	0.202	1	0	cassette	0.202
39	ENSG00000	ENSG00000	CRLS1	chr20	+	ENSG00000	ENSG00000	TRUE	cassette^2|i	TRUE	Distal	chr20:60262	FALSE	FALSE	TRUE	0.008	0.213	0.187	1	0	AFE/ALE	0.187
40	ENSG00000	ENSG00000	DNAAF9	chr20	-	ENSG00000	ENSG00000	TRUE	cassette^1|i	TRUE	C1_A	chr20:33446	FALSE	TRUE	TRUE	0.002	0.441	0.426	1	0	cassette	0.426
41	ENSG00000	ENSG00000	GRAMD1A	chr19	+	ENSG00000	ENSG00000	TRUE	cassette^1|i	TRUE	C1_A	chr19:35000	FALSE	TRUE	TRUE	0.006	0.185	0.161	1	0	cassette	0.161
42	ENSG00000	ENSG00000	GRAMD1A	chr19	+	ENSG00000	ENSG00000	TRUE	cassette^1|i	FALSE	Distal	chr19:35001	FALSE	FALSE	TRUE	0.025	0.314	0.293	1	0	AFE/ALE	0.293
43	ENSG00000	ENSG00000	AARS1	chr16	-	ENSG00000	ENSG00000	TRUE	cassette^1|i	TRUE	C1_A	chr16:70272	FALSE	TRUE	TRUE	0	0.286	0.27	1	0	cassette	0.27
44	ENSG00000	ENSG00000	G2E3	chr14	+	ENSG00000	ENSG00000	TRUE	cassette^1|i	TRUE	Proximal	chr14:30559	FALSE	FALSE	TRUE	0.001	0.136	0.11	0.956	0.021	AFE/ALE	0.11
45	ENSG00000	ENSG00000	NUP188	chr9	+	ENSG00000	ENSG00000	FALSE	cassette^1	TRUE	C1_A	chr9:128952	FALSE	TRUE	TRUE	0.003	0.783	0.781	1	0	cassette	0.781
46	ENSG00000	ENSG00000	RANBP1	chr22	+	ENSG00000	ENSG00000	FALSE	cassette^1	FALSE	C2_A	chr22:20122	FALSE	FALSE	TRUE	0.003	0.123	0.102	0.981	0.01	cassette	0.102
47	ENSG00000	ENSG00000	BCL2L13	chr22	+	ENSG00000	ENSG00000	TRUE	cassette^7|t	TRUE	J3	chr22:17702	FALSE	FALSE	TRUE	0.006	0.134	0.107	0.963	0.019	cassette	0.107
48	ENSG000001	ENSG000001	SNRPD3	chr22	+	ENSG000001	ENSG000001	TRUE	ir^2|putative	TRUE	Proximal	chr22:24572	FALSE	FALSE	TRUE	0.004	0.188	0.162	0.993	0.002	AFE/ALE	0.162
49	ENSG000001	ENSG000001	HDAC10	chr22	-	ENSG000001	ENSG000001	TRUE	cassette^1|t	TRUE	C2_A_Last	chr22:50248	FALSE	FALSE	TRUE	0.043	0.224	0.174	0.991	0.002	cassette	0.174
50	ENSG000001	ENSG000001	GSS	chr20	-	ENSG000001	ENSG000001	FALSE	ale^1	FALSE	Proximal	chr20:34950	FALSE	TRUE	TRUE	0.001	0.231	0.202	1	0	AFE/ALE	0.202
51	ENSG000001	ENSG000001	PRELID3B	chr20	-	ENSG000001	ENSG000001	TRUE	cassette^2|a	FALSE	C1_C2	chr20:59036	FALSE	FALSE	TRUE	0.001	0.157	-0.132	0.998	0.001	skipping	0.132
52	ENSG000001	ENSG000001	ATP11C	chrX	-	ENSG000001	ENSG000001	TRUE	cassette^5|p	FALSE	C1_A	chrX:139737	FALSE	FALSE	TRUE	0.041	0.291	0.241	0.997	0	cassette	0.241
53	ENSG000001	ENSG000001	EEA1	chr12	-	ENSG000001	ENSG000001	FALSE	cassette^1	TRUE	C1_A	chr12:92846	FALSE	FALSE	TRUE	0.014	0.255	0.231	1	0	cassette	0.231
54	ENSG000001	ENSG000001	ZNF423	chr16	-	ENSG000001	ENSG000001	FALSE	cassette^1	TRUE	C1_A	chr16:49640	FALSE	TRUE	TRUE	0.001	0.41	0.391	1	0	cassette	0.391
55	ENSG000001	ENSG000001	NECAB2	chr16	+	ENSG000001	ENSG000001	TRUE	cassette^1|i	TRUE	C1_A	chr16:83990	FALSE	TRUE	TRUE	0.001	0.409	0.389	1	0	cassette	0.389
56	ENSG000001	ENSG000001	USP10	chr16	+	ENSG000001	ENSG000001	FALSE	cassette^1	TRUE	C2_A	chr16:84735	FALSE	FALSE	TRUE	0.001	0.192	0.169	1	0	cassette	0.169
57	ENSG000001	ENSG000001	CORO7-PAM	chr16	-	ENSG000001	ENSG000001	FALSE	putative_ale	TRUE	Proximal	chr16:43674	FALSE	TRUE	TRUE	0.019	0.347	0.323	1	0	AFE/ALE	0.323
58	ENSG000001	ENSG000001	STMN2	chr8	+	ENSG000001	ENSG000001	TRUE	ir^2|ale^2|p	TRUE	Distal	chr8:796112	FALSE	FALSE	TRUE	0.003	0.637	0.624	1	0	AFE/ALE	0.624
59	ENSG000001	ENSG000001	SLC1A6	chr19	-	ENSG000001	ENSG000001	TRUE	cassette^2|a	TRUE	C1_A	chr19:14979	FALSE	FALSE	TRUE	0	0.139	0.113	0.985	0.007	cassette	0.113
60	ENSG000001	ENSG000001	GPI	chr19	+	ENSG000001	ENSG000001	TRUE	alt3^1|alt3_	TRUE	C2_A2	chr19:34392	FALSE	FALSE	TRUE	0	0.127	0.101	0.965	0.018	cassette	0.101
61	ENSG000001	ENSG000001	CCDC9	chr19	+	ENSG000001	ENSG000001	FALSE	alt5^1	TRUE	Proximal	chr19:47271	FALSE	FALSE	TRUE	0.004	0.187	0.16	0.994	0.002	AFE/ALE	0.16
62	ENSG000001	ENSG000001	DENND3	chr8	+	ENSG000001	ENSG000001	FALSE	putative_alt5	FALSE	Proximal	chr8:141173	FALSE	FALSE	FALSE	0.023	0.176	0.132	0.923	0.02	AFE/ALE	0.132
63	ENSG000001	ENSG000001	MAST1	chr19	+	ENSG000001	ENSG000001	TRUE	cassette^2|a	TRUE	C1_A	chr19:12841	FALSE	FALSE	TRUE	0	0.275	0.254	1	0	cassette	0.254
64	ENSG000001	ENSG000001	MAST1	chr19	+	ENSG000001	ENSG000001	TRUE	cassette^2|a	TRUE	Distal	chr19:12842	FALSE	FALSE	TRUE	0	0.151	0.124	1	0	AFE/ALE	0.124
65	ENSG000001	ENSG000001	SIPA1L3	chr19	+	ENSG000001	ENSG000001	TRUE	ale^2|putati	TRUE	Distal	chr19:37907	FALSE	FALSE	TRUE	0.003	0.377	0.353	1	0	AFE/ALE	0.353
66	ENSG000001	ENSG000001	RUNDC3B	chr7	+	ENSG000001	ENSG000001	TRUE	cassette^3|a	TRUE	Distal	chr7:876285	FALSE	TRUE	TRUE	0.001	0.261	0.238	1	0	AFE/ALE	0.238
67	ENSG000001	ENSG000001	IQCE	chr7	+	ENSG000001	ENSG000001	TRUE	cassette^3|i	TRUE	C1_A	chr7:255921	FALSE	TRUE	TRUE	0.001	0.621	0.608	1	0	cassette	0.608
68	ENSG000001	ENSG000001	IQCE	chr7	+	ENSG000001	ENSG000001	TRUE	cassette^3|i	TRUE	Proximal	chr7:255921	FALSE	TRUE	TRUE	0.013	0.202	0.181	1	0	AFE/ALE	0.181
69	ENSG000001	ENSG000001	WASL	chr7	-	ENSG000001	ENSG000001	TRUE	cassette^2|a	TRUE	C1_C2	chr7:123684	FALSE	TRUE	TRUE	0.001	0.554	-0.547	1	0	skipping	0.547
70	ENSG000001	ENSG000001	WASL	chr7	-	ENSG000001	ENSG000001	TRUE	cassette^2|a	TRUE	C2_A	chr7:123684	FALSE	FALSE	FALSE	0	0.119	0.096	0.922	0.042	cassette	0.096
71	ENSG000001	ENSG000001	DNM1	chr9	+	ENSG000001	ENSG000001	TRUE	alt3^1|ir^2	TRUE	Proximal	chr9:128250	FALSE	TRUE	TRUE	0.001	0.204	0.181	1	0	AFE/ALE	0.181
72	ENSG000001	ENSG000001	NSMCE4A	chr10	-	ENSG000001	ENSG000001	TRUE	cassette^1|i	TRUE	C1_A	chr10:12197	FALSE	FALSE	TRUE	0.019	0.261	0.232	1	0	cassette	0.232
73	ENSG000001	ENSG000001	SPOCK2	chr10	-	ENSG000001	ENSG000001	TRUE	alt3_5^1|ir^	TRUE	E1_E2_J2	chr10:72088	FALSE	FALSE	FALSE	0.01	0.131	0.103	0.931	0.034	cassette	0.103
74	ENSG000001	ENSG000001	DNAJC12	chr10	-	ENSG000001	ENSG000001	TRUE	cassette^1|p	TRUE	C2_A	chr10:67823	FALSE	FALSE	FALSE	0.036	0.153	0.116	0.94	0.027	cassette	0.116
75	ENSG000001	ENSG000001	MPP2	chr17	-	ENSG000001	ENSG000001	TRUE	cassette^1|a	FALSE	C1_A	chr17:43884	FALSE	FALSE	FALSE	0.026	0.117	0.094	0.917	0.043	cassette	0.094

76	ENSG000001	ENSG000001	MADD	chr11	+	ENSG000001	ENSG000001	TRUE	cassette^4|	TRUE	C1_A	chr11:47325	FALSE	FALSE	TRUE	0.005	0.195	0.17	0.999	0	cassette	0.17
77	ENSG000001	ENSG000001	MADD	chr11	+	ENSG000001	ENSG000001	TRUE	cassette^4|	FALSE	C1_A	chr11:47324	FALSE	FALSE	TRUE	0.003	0.326	0.3	1	0	cassette	0.3
78	ENSG000001	ENSG000001	MADD	chr11	+	ENSG000001	ENSG000001	TRUE	cassette^4|	TRUE	Proximal	chr11:47325	FALSE	TRUE	TRUE	0.009	0.248	0.222	1	0	AFE/ALE	0.222
79	ENSG000001	ENSG000001	MADD	chr11	+	ENSG000001	ENSG000001	TRUE	cassette^4|	TRUE	Proximal	chr11:47325	FALSE	TRUE	TRUE	0.003	0.281	0.259	1	0	AFE/ALE	0.259
80	ENSG000001	ENSG000001	RNGTT	chr6	-	ENSG000001	ENSG000001	FALSE	alt5^1	TRUE	Proximal	chr6:889049	FALSE	FALSE	TRUE	0.039	0.179	0.141	0.97	0.011	AFE/ALE	0.141
81	ENSG000001	ENSG000001	FRMD4B	chr3	-	ENSG000001	ENSG000001	TRUE	ir^1|ale^1|	TRUE	Proximal	chr3:693024	FALSE	FALSE	TRUE	0.009	0.221	0.186	0.993	0.001	AFE/ALE	0.186
82	ENSG000001	ENSG000001	CCDC88A	chr2	-	ENSG000001	ENSG000001	TRUE	cassette^3|	FALSE	A_C2	chr2:553006	FALSE	FALSE	TRUE	0.003	0.29	0.265	1	0	cassette	0.265
83	ENSG000001	ENSG000001	EHBP1	chr2	+	ENSG000001	ENSG000001	FALSE	cassette^1	FALSE	C1_C2	chr2:628311	FALSE	FALSE	TRUE	0.038	0.209	-0.171	0.997	0.001	skipping	0.171
84	ENSG000001	ENSG000001	ELAPOR1	chr1	+	ENSG000001	ENSG000001	TRUE	cassette^1|i	TRUE	C1_C2	chr1:109197	FALSE	TRUE	TRUE	0.018	0.241	-0.216	1	0	skipping	0.216
85	ENSG000001	ENSG000001	DLGAP3	chr1	-	ENSG000001	ENSG000001	TRUE	cassette^1|	TRUE	C1_A	chr1:349290	FALSE	FALSE	TRUE	0.001	0.232	0.206	1	0	cassette	0.206
86	ENSG000001	ENSG000001	AKT3	chr1	-	ENSG000001	ENSG000001	FALSE	tandem_cass	TRUE	C1_A	chr1:243527	FALSE	TRUE	TRUE	0.002	0.228	0.203	1	0	cassette	0.203
87	ENSG000001	ENSG000001	IGSF21	chr1	+	ENSG000001	ENSG000001	FALSE	cassette^1	TRUE	C1_A	chr1:182280	FALSE	FALSE	TRUE	0.003	0.494	0.481	1	0	cassette	0.481
88	ENSG000001	ENSG000001	ERI3	chr1	-	ENSG000001	ENSG000001	TRUE	cassette^2|	FALSE	C1_A	chr1:442420	FALSE	FALSE	TRUE	0.011	0.171	0.149	1	0	cassette	0.149
89	ENSG000001	ENSG000001	PROSER1	chr13	-	ENSG000001	ENSG000001	TRUE	cassette^2|	TRUE	C1_A	chr13:39018	FALSE	FALSE	TRUE	0.001	0.14	0.118	0.988	0.005	cassette	0.118
90	ENSG000001	ENSG000001	NFYB	chr12	-	ENSG000001	ENSG000001	TRUE	cassette^1|	TRUE	Proximal	chr12:10411	FALSE	FALSE	TRUE	0.001	0.145	0.119	0.968	0.015	AFE/ALE	0.119
91	ENSG000001	ENSG000001	WASHC3	chr12	-	ENSG000001	ENSG000001	TRUE	cassette^2|	FALSE	C2_A	chr12:10204	FALSE	FALSE	FALSE	0.018	0.125	0.1	0.921	0.04	cassette	0.1
92	ENSG000001	ENSG000001	APAF1	chr12	+	ENSG000001	ENSG000001	TRUE	alt5^3	TRUE	Proximal	chr12:98649	FALSE	FALSE	TRUE	0.021	0.179	0.145	0.97	0.01	AFE/ALE	0.145
93	ENSG000001	ENSG000001	ADCY7	chr16	+	ENSG000001	ENSG000001	TRUE	cassette^1|	TRUE	C1_A	chr16:50246	FALSE	TRUE	TRUE	0.002	0.317	0.296	1	0	cassette	0.296
94	ENSG000001	ENSG000001	GLIPR2	chr9	+	ENSG000001	ENSG000001	FALSE	ale^1	FALSE	Distal	chr9:361486	FALSE	FALSE	FALSE	0.026	0.211	0.154	0.918	0.017	AFE/ALE	0.154
95	ENSG000001	ENSG000001	CNTFR	chr9	-	ENSG000001	ENSG000001	TRUE	cassette^2|afe^4|putativ		C1_A	chr9:	FALSE	FALSE	TRUE	0.004	0.165	0.138	0.992	0.003	cassette	0.138
96	ENSG000001	ENSG000001	CYREN	chr7	-	ENSG000001	ENSG000001	TRUE	alt5^1|multi	FALSE	C1_A	chr7:135167	FALSE	FALSE	TRUE	0.006	0.17	0.138	0.978	0.008	cassette	0.138
97	ENSG000001	ENSG000001	MED13L	chr12	-	ENSG000001	ENSG000001	FALSE	cassette^1	TRUE	C1_A	chr12:11605	FALSE	FALSE	TRUE	0.001	0.147	0.124	0.996	0.002	cassette	0.124
98	ENSG000001	ENSG000001	STX16	chr20	+	ENSG000001	ENSG000001	FALSE	cassette^1	FALSE	C2_C1	chr20:58670	FALSE	FALSE	TRUE	0.014	0.126	-0.101	0.951	0.025	skipping	0.101
99	ENSG000001	ENSG000001	ATXN1	chr6	-	ENSG000001	ENSG000001	TRUE	cassette^3|	TRUE	C1_A	chr6:165895	FALSE	FALSE	TRUE	0.001	0.366	0.347	1	0	cassette	0.347
100	ENSG000001	ENSG000001	ATXN1	chr6	-	ENSG000001	ENSG000001	TRUE	cassette^3|alt5^3|ir^2|a		A_C2	chr6:	FALSE	FALSE	TRUE	0.001	0.184	0.161	1	0	cassette	0.161
101	ENSG000001	ENSG000001	TM9SF2	chr13	+	ENSG000001	ENSG000001	TRUE	alt3^1|ir^5|	TRUE	Proximal	chr13:99536	FALSE	FALSE	TRUE	0	0.149	0.127	0.96	0.017	AFE/ALE	0.127
102	ENSG000001	ENSG000001	TM9SF2	chr13	+	ENSG000001	ENSG000001	TRUE	alt3^1|ir^5|	TRUE	Distal	chr13:99536	FALSE	FALSE	TRUE	0.001	0.143	0.119	0.953	0.021	AFE/ALE	0.119
103	ENSG000001	ENSG000001	TM9SF2	chr13	+	ENSG000001	ENSG000001	TRUE	alt3^1|ir^5|	TRUE	Distal	chr13:99536	FALSE	FALSE	FALSE	0.001	0.139	0.115	0.949	0.023	AFE/ALE	0.115
104	ENSG000001	ENSG000001	TMEM175	chr4	+	ENSG000001	ENSG000001	TRUE	cassette^1|i	FALSE	C1_A	chr4:932540	FALSE	FALSE	TRUE	0	0.165	0.145	1	0	cassette	0.145
105	ENSG000001	ENSG000001	MACF1	chr1	+	ENSG000001	ENSG000001	TRUE	cassette^1|i	FALSE	C1_A	chr1:394357	FALSE	FALSE	TRUE	0.036	0.13	0.099	0.957	0.023	cassette	0.099
106	ENSG000001	ENSG000001	LRFN1	chr19	-	ENSG000001	ENSG000001	TRUE	cassette^1|i	TRUE	C1_A	chr19:39316	FALSE	FALSE	TRUE	0.001	0.144	0.119	0.996	0.002	cassette	0.119
107	ENSG000001	ENSG000001	EPB41L4A	chr5	-	ENSG000001	ENSG000001	FALSE	cassette^1	TRUE	C1_A	chr5:112267	FALSE	TRUE	TRUE	0.024	0.895	0.882	1	0	cassette	0.882
108	ENSG000001	ENSG000001	CDO1	chr5	-	ENSG000001	ENSG000001	TRUE	cassette^1|i	FALSE	C1_A	chr5:115813	FALSE	TRUE	TRUE	0.022	0.315	0.295	1	0	cassette	0.295
109	ENSG000001	ENSG000001	UNC13A	chr19	-	ENSG000001	ENSG000001	TRUE	cassette^2|	TRUE	C1_A	chr19:17642	FALSE	TRUE	TRUE	0	0.492	0.479	1	0	cassette	0.479
110	ENSG000001	ENSG000001	UNC13A	chr19	-	ENSG000001	ENSG000001	TRUE	cassette^2|	TRUE	C2_A	chr19:17641	FALSE	TRUE	TRUE	0	0.42	0.403	1	0	cassette	0.403
111	ENSG000001	ENSG000001	PXDN	chr2	-	ENSG000001	ENSG000001	TRUE	cassette^1|i	TRUE	C1_A	chr2:165088	FALSE	TRUE	TRUE	0.003	0.449	0.436	1	0	cassette	0.436
112	ENSG000001	ENSG000001	CAMSAP1	chr9	-	ENSG000001	ENSG000001	TRUE	putative_afe	TRUE	Proximal	chr9:135838	FALSE	FALSE	TRUE	0.003	0.151	0.124	0.972	0.012	AFE/ALE	0.124
113	ENSG000001	ENSG000001	SYNE1	chr6	-	ENSG000001	ENSG000001	FALSE	cassette^1	TRUE	C1_A	chr6:152247	FALSE	TRUE	TRUE	0.001	0.216	0.188	1	0	cassette	0.188
114	ENSG000001	ENSG000001	ZNF141	chr4	+	ENSG000001	ENSG000001	TRUE	ir^1|putative	TRUE	Proximal	chr4:373234	FALSE	TRUE	TRUE	0.003	0.346	0.32	1	0	AFE/ALE	0.32
115	ENSG000001	ENSG000001	GSE1	chr16	+	ENSG000001	ENSG000001	TRUE	cassette^2|	TRUE	Orphan	chr16:85463	FALSE	FALSE	TRUE	0.003	0.167	0.145	0.995	0.002	cassette	0.145
116	ENSG000001	ENSG000001	MGAT1	chr5	-	ENSG000001	ENSG000001	TRUE	alt5^3|afe^1	TRUE	Distal	chr5:180809	FALSE	TRUE	TRUE	0.001	0.3	0.278	1	0	AFE/ALE	0.278
117	ENSG000001	ENSG000001	NUP210	chr3	-	ENSG000001	ENSG000001	TRUE	tandem_cass	FALSE	C1_A	chr3:133738	FALSE	TRUE	TRUE	0.014	0.304	0.279	1	0	cassette	0.279
118	ENSG000001	ENSG000001	RIN2	chr20	+	ENSG000001	ENSG000001	TRUE	cassette^1|i	TRUE	C1_A	chr20:19965	FALSE	TRUE	TRUE	0.001	0.241	0.222	1	0	cassette	0.222
119	ENSG000001	ENSG000001	XPO4	chr13	-	ENSG000001	ENSG000001	FALSE	alt5^1	TRUE	Proximal	chr13:20799	FALSE	FALSE	TRUE	0.013	0.205	0.18	0.994	0.002	AFE/ALE	0.18
120	ENSG000001	ENSG000001	CDK7	chr5	+	ENSG000001	ENSG000001	TRUE	cassette^1|i	TRUE	C2_A	chr5:692614	FALSE	FALSE	TRUE	0.001	0.28	0.258	1	0	cassette	0.258
121	ENSG000001	ENSG000001	KATNBL1	chr15	-	ENSG000001	ENSG000001	TRUE	ale^4|multi_	FALSE	Proximal	chr15:34143	FALSE	FALSE	TRUE	0.013	0.174	0.15	0.988	0.004	AFE/ALE	0.15
122	ENSG000001	ENSG000001	ARHGAP32	chr11	-	ENSG000001	ENSG000001	FALSE	putative_ale	TRUE	Proximal	chr11:12899	FALSE	TRUE	TRUE	0.009	0.709	0.703	1	0	AFE/ALE	0.703
123	ENSG000001	ENSG000001	TAOK3	chr12	-	ENSG000001	ENSG000001	FALSE	cassette^1	TRUE	C1_A	chr12:11815	FALSE	FALSE	TRUE	0.019	0.165	0.144	1	0	cassette	0.144
124	ENSG000001	ENSG000001	MICAL1	chr6	-	ENSG000001	ENSG000001	TRUE	cassette^6|	TRUE	C2_A	chr6:109447	FALSE	FALSE	TRUE	0.007	0.138	0.111	0.976	0.012	cassette	0.111
125	ENSG000001	ENSG000001	TGFBRAP1	chr2	-	ENSG000001	ENSG000001	FALSE	alt5^1	TRUE	Proximal	chr2:105298	FALSE	FALSE	TRUE	0.007	0.137	0.113	0.955	0.021	AFE/ALE	0.113
126	ENSG000001	ENSG000001	ANKRD36	chr2	+	ENSG000001	ENSG000001	TRUE	cassette^1|i	TRUE	C1_C2	chr2:972476	FALSE	FALSE	TRUE	0.007	0.249	-0.221	1	0	skipping	0.221
127	ENSG000001	ENSG000001	LMO7	chr13	+	ENSG000001	ENSG000001	TRUE	cassette^1|	FALSE	Distal	chr13:75804	FALSE	FALSE	FALSE	0.022	0.2	0.152	0.921	0.016	AFE/ALE	0.152
128	ENSG000001	ENSG000001	CBWD2	chr2	+	ENSG000001	ENSG000001	TRUE	ir^2|putative	TRUE	Proximal	chr2:113465	FALSE	FALSE	TRUE	0.02	0.2	0.162	0.988	0.003	AFE/ALE	0.162
129	ENSG000001	ENSG000001	SEMA6D	chr15	+	ENSG000001	ENSG000001	TRUE	cassette^6|	TRUE	C1_A	chr15:47412	FALSE	TRUE	TRUE	0.002	0.217	0.197	1	0	cassette	0.197
130	ENSG000001	ENSG000001	ACTR1A	chr10	-	ENSG000001	ENSG000001	TRUE	ir^1|putative	TRUE	Proximal	chr10:10247	FALSE	FALSE	TRUE	0.011	0.181	0.152	0.984	0.004	AFE/ALE	0.152
131	ENSG000001	ENSG000001	SEPTIN11	chr4	+	ENSG000001	ENSG000001	TRUE	cassette^1|i	TRUE	C1_A	chr4:770309	FALSE	FALSE	TRUE	0.005	0.171	0.152	1	0	cassette	0.152
132	ENSG000001	ENSG000001	KIF21A	chr12	-	ENSG000001	ENSG000001	TRUE	cassette^1|i	FALSE	C1_C2	chr12:39326	FALSE	FALSE	FALSE	0.03	0.119	-0.096	0.932	0.036	skipping	0.096
133	ENSG000001	ENSG000001	KIF21A	chr12	-	ENSG000001	ENSG000001	TRUE	cassette^1|	TRUE	C1_A	chr12:39389	FALSE	FALSE	TRUE	0.001	0.248	0.224	1	0	cassette	0.224
134	ENSG000001	ENSG000001	KIF21A	chr12	-	ENSG000001	ENSG000001	TRUE	cassette^1|	TRUE	Proximal	chr12:39370	FALSE	FALSE	TRUE	0.001	0.135	0.11	1	0	AFE/ALE	0.11
135	ENSG000001	ENSG000001	TMEM117	chr12	+	ENSG000001	ENSG000001	FALSE	ale^1	TRUE	Proximal	chr12:43836	FALSE	TRUE	TRUE	0.006	0.812	0.796	1	0	AFE/ALE	0.796
136	ENSG000001	ENSG000001	SRRM4	chr12	+	ENSG000001	ENSG000001	TRUE	cassette^2|ir^6|multi_ex		A_C2	chr12:	FALSE	FALSE	TRUE	0.013	0.195	0.162	0.997	0.001	cassette	0.162
137	ENSG000001	ENSG000001	POLG	chr15	-	ENSG000001	ENSG000001	FALSE	cassette^1	FALSE	C2_C1	chr15:89322	FALSE	FALSE	TRUE	0.02	0.189	-0.154	0.972	0.007	skipping	0.154
138	ENSG000001	ENSG000001	NTRK3	chr15	-	ENSG000001	ENSG000001	TRUE	alt5^1|ir^2	TRUE	Proximal	chr15:88256	FALSE	FALSE	TRUE	0.001	0.132	0.105	0.955	0.023	AFE/ALE	0.105
139	ENSG000001	ENSG000001	NKD1	chr16	+	ENSG000001	ENSG000001	TRUE	cassette^2|	TRUE	C1_A	chr16:50608	FALSE	FALSE	TRUE	0.001	0.408	0.391	1	0	cassette	0.391
140	ENSG000001	ENSG000001	NKD1	chr16	+	ENSG000001	ENSG000001	TRUE	cassette^2|	TRUE	Proximal	chr16:50620	FALSE	FALSE	TRUE	0.001	0.456	0.443	1	0	AFE/ALE	0.443
141	ENSG000001	ENSG000001	PRELID3A	chr18	+	ENSG000001	ENSG000001	TRUE	cassette^1|	TRUE	C1_A	chr18:12429	FALSE	TRUE	TRUE	0.003	0.529	0.512	1	0	cassette	0.512
142	ENSG000001	ENSG000001	CEP131	chr17	-	ENSG000001	ENSG000001	TRUE	putative_afe	TRUE	Distal	chr17:81207	FALSE	FALSE	TRUE	0.001	0.17	0.144	0.995	0.002	AFE/ALE	0.144
143	ENSG000001	ENSG000001	FBN3	chr19	-	ENSG000001	ENSG000001	TRUE	cassette^1|i	TRUE	C1_A	chr19:80844	FALSE	TRUE	TRUE	0.002	0.74	0.733	1	0	cassette	0.733
144	ENSG000001	ENSG000001	IGLON5	chr19	+	ENSG000001	ENSG000001	FALSE	cassette^1	TRUE	C1_A	chr19:51311	FALSE	TRUE	TRUE	0.001	0.24	0.215	1	0	cassette	0.215
145	ENSG000001	ENSG000001	NVL	chr1	-	ENSG000001	ENSG000001	TRUE	cassette^8|	TRUE	C2_A	chr1:224311	FALSE	FALSE	TRUE	0.036	0.185	0.151	0.974	0.008	cassette	0.151
146	ENSG000001	ENSG000001	CNGA3	chr2	+	ENSG000001	ENSG000001	FALSE	putative_afe	TRUE	Proximal	chr2:983760	FALSE	TRUE	TRUE	0.038	0.301	0.253	0.995	0	AFE/ALE	0.253
147	ENSG000001	ENSG000001	GOLGA4	chr3	+	ENSG000001	ENSG000001	TRUE	cassette^2|	TRUE	C1_A	chr3:372514	FALSE	FALSE	FALSE	0.008	0.133	0.103	0.903	0.049	cassette	0.103
148	ENSG000001	ENSG000001	SHQ1	chr3	-	ENSG000001	ENSG000001	TRUE	cassette^1|	TRUE	Proximal	chr3:727508	FALSE	FALSE	TRUE	0.001	0.306	0.286	1	0	AFE/ALE	0.286
149	ENSG000001	ENSG000001	STXBP5L	chr3	+	ENSG000001	ENSG000001	TRUE	alt5^3|afe^	TRUE	Proximal	chr3:120908	FALSE	TRUE	TRUE	0.001	0.222	0.193	1	0	AFE/ALE	0.193
150	ENSG000001	ENSG000001	STXBP5L	chr3	+	ENSG000001	ENSG000001	TRUE	alt5^3|afe^	TRUE	Proximal	chr3:120908	FALSE	TRUE	TRUE	0.001	0.409	0.382	1	0	AFE/ALE	0.382
151	ENSG000001	ENSG000001	G3BP1	chr5	+	ENSG000001	ENSG000001	FALSE	cassette^1	FALSE	C1_A	chr5:151786	FALSE	TRUE	TRUE	0.033	0.571	0.566	1	0	cassette	0.566

152	ENSG000001	ENSG000001	SCUBE3	chr6	+	ENSG000001	ENSG000001	TRUE	cassette^3|a	TRUE	C2_A	chr6:352181	FALSE	FALSE	TRUE	0.045	0.23	0.191	1	0	cassette	0.191
153	ENSG000001	ENSG000001	WASH2P	chr2	+	ENSG000001	ENSG000001	FALSE	alt5^1	TRUE	Proximal	chr2:113598	FALSE	FALSE	FALSE	0.009	0.167	0.137	0.946	0.017	AFE/ALE	0.137
154	ENSG000001	ENSG000001	UTP23	chr8	+	ENSG000001	ENSG000001	TRUE	ir^2|ale^2|r	FALSE	Distal	chr8:116770	FALSE	FALSE	FALSE	0.045	0.253	0.184	0.932	0.008	AFE/ALE	0.184
155	ENSG000001	ENSG000001	POLR3A	chr10	-	ENSG000001	ENSG000001	FALSE	cassette^1	FALSE	C1_C2	chr10:78007	FALSE	FALSE	TRUE	0.028	0.214	-0.184	0.999	0	skipping	0.184
156	ENSG000001	ENSG000001	INTS4	chr11	-	ENSG000001	ENSG000001	TRUE	putative_alt5	TRUE	C1_A	chr11:77883	FALSE	FALSE	TRUE	0.031	0.742	0.72	1	0	cassette	0.72
157	ENSG000001	ENSG000001	HYOU1	chr11	-	ENSG000001	ENSG000001	FALSE	cassette^1	FALSE	C1_C2	chr11:11905	FALSE	FALSE	TRUE	0.02	0.176	-0.155	1	0	skipping	0.155
158	ENSG000001	ENSG000001	FEZ1	chr11	-	ENSG000001	ENSG000001	TRUE	alt5^1|afe^2	FALSE	Proximal	chr11:12548	FALSE	TRUE	TRUE	0.027	0.278	0.258	1	0	AFE/ALE	0.258
159	ENSG000001	ENSG000001	UBC	chr12	-	ENSG000001	ENSG000001	TRUE	putative_alt3	FALSE	C1_A	chr12:12491	FALSE	FALSE	TRUE	0.003	0.182	0.157	1	0	cassette	0.157
160	ENSG000001	ENSG000001	UBC	chr12	-	ENSG000001	ENSG000001	TRUE	putative_alt3	FALSE	A_C2	chr12:12491	FALSE	FALSE	TRUE	0.024	0.119	0.102	0.991	0.005	cassette	0.102
161	ENSG000001	ENSG000001	DLG5	chr10	-	ENSG000001	ENSG000001	TRUE	cassette^1|i	TRUE	C1_A	chr10:77793	FALSE	FALSE	TRUE	0.001	0.14	0.116	0.999	0	cassette	0.116
162	ENSG000001	ENSG000001	RNF144A	chr2	+	ENSG000001	ENSG000001	TRUE	ale^2|putati	TRUE	Proximal	chr2:703021	FALSE	TRUE	TRUE	0.032	0.289	0.261	1	0	AFE/ALE	0.261
163	ENSG000001	ENSG000001	RNF144A	chr2	+	ENSG000001	ENSG000001	TRUE	ale^2|putati	TRUE	Distal	chr2:703021	FALSE	FALSE	FALSE	0.036	0.152	0.112	0.902	0.039	AFE/ALE	0.112
164	ENSG000001	ENSG000001	GUF1	chr4	+	ENSG000001	ENSG000001	TRUE	ir^2|putative	TRUE	Proximal	chr4:446927	FALSE	FALSE	TRUE	0.034	0.19	0.157	0.983	0.005	AFE/ALE	0.157
165	ENSG000001	ENSG000001	PTPRD	chr9	-	ENSG000001	ENSG000001	TRUE	cassette^4|t	TRUE	C1_A	chr9:912720	FALSE	FALSE	TRUE	0.006	0.612	0.602	1	0	cassette	0.602
166	ENSG000001	ENSG000001	PTPRD	chr9	-	ENSG000001	ENSG000001	TRUE	cassette^4|t	TRUE	C1_A	chr9:912720	FALSE	TRUE	TRUE	0.019	0.645	0.634	1	0	cassette	0.634
167	ENSG000001	ENSG000001	PTPRD	chr9	-	ENSG000001	ENSG000001	TRUE	cassette^4|t	TRUE	C2_A_Last	chr9:901873	FALSE	FALSE	TRUE	0.026	0.575	0.566	1	0	cassette	0.566
168	ENSG000001	ENSG000001	PTPRD	chr9	-	ENSG000001	ENSG000001	TRUE	cassette^3|a	TRUE	E2_E1_J1	chr9:939658	FALSE	FALSE	TRUE	0	0.173	0.144	0.958	0.008	cassette	0.144
169	ENSG000001	ENSG000001	PTPRD	chr9	-	ENSG000001	ENSG000001	TRUE	cassette^3|a	TRUE	Proximal	chr9:944222	FALSE	TRUE	TRUE	0	0.314	0.29	1	0	AFE/ALE	0.29
170	ENSG000001	ENSG000001	UBASH3B	chr11	+	ENSG000001	ENSG000001	FALSE	cassette^1	TRUE	C1_A	chr11:12265	FALSE	TRUE	TRUE	0.002	0.573	0.56	1	0	cassette	0.56
171	ENSG000001	ENSG000001	PTPRN2	chr7	-	ENSG000001	ENSG000001	TRUE	putative_ale	TRUE	Proximal	chr7:157875	FALSE	TRUE	TRUE	0.003	0.245	0.208	0.996	0.001	AFE/ALE	0.208
172	ENSG000001	ENSG000001	PTPRN2	chr7	-	ENSG000001	ENSG000001	TRUE	ale^3|afe^1|multi_exon_s		C1_A	chr7:	FALSE	FALSE	TRUE	0.001	0.328	0.304	1	0	cassette	0.304
173	ENSG000001	ENSG000001	PTPRN2	chr7	-	ENSG000001	ENSG000001	TRUE	cassette^3|p	TRUE	C1_A	chr7:158249	FALSE	FALSE	TRUE	0.02	0.19	0.165	1	0	cassette	0.165
174	ENSG000001	ENSG000001	GOLGA7B	chr10	+	ENSG000001	ENSG000001	TRUE	cassette^1|i	TRUE	C1_A	chr10:97866	FALSE	FALSE	TRUE	0.003	0.349	0.325	1	0	cassette	0.325
175	ENSG000001	ENSG000001	GOLGA7B	chr10	+	ENSG000001	ENSG000001	TRUE	cassette^1|i	TRUE	Proximal	chr10:97866	FALSE	FALSE	TRUE	0.015	0.401	0.382	1	0	AFE/ALE	0.382
176	ENSG000001	ENSG000001	ADCY8	chr8	-	ENSG000001	ENSG000001	FALSE	putative_ale	TRUE	Proximal	chr8:130909	FALSE	TRUE	TRUE	0.011	0.358	0.325	1	0	AFE/ALE	0.325
177	ENSG000001	ENSG000001	KCNMA1	chr10	-	ENSG000001	ENSG000001	TRUE	cassette^1|i	FALSE	C1_C2	chr10:77019	FALSE	FALSE	TRUE	0.003	0.187	-0.163	0.988	0.003	skipping	0.163
178	ENSG000001	ENSG000001	ETS2	chr21	+	ENSG000001	ENSG000001	FALSE	cassette^1	FALSE	C2_A	chr21:38806	FALSE	FALSE	TRUE	0.002	0.2	0.175	1	0	cassette	0.175
179	ENSG000001	ENSG000001	TSPAN18	chr11	+	ENSG000001	ENSG000001	FALSE	putative_alt5	TRUE	Intron	chr11:44920	FALSE	FALSE	TRUE	0.048	0.239	0.181	0.985	0.003	cassette	0.181
180	ENSG000001	ENSG000001	WIPI2	chr7	+	ENSG000001	ENSG000001	FALSE	cassette^1	TRUE	C2_C1	chr7:519049	FALSE	FALSE	FALSE	0.03	0.143	-0.111	0.935	0.029	skipping	0.111
181	ENSG000001	ENSG000001	UBXN11	chr1	-	ENSG000001	ENSG000001	TRUE	cassette^5|t	FALSE	A_C2	chr1:262943	FALSE	FALSE	FALSE	0.046	0.188	0.13	0.905	0.03	cassette	0.13
182	ENSG000001	ENSG000001	EYA3	chr1	-	ENSG000001	ENSG000001	FALSE	cassette^1	TRUE	C1_A	chr1:280566	FALSE	TRUE	TRUE	0.002	0.217	0.195	1	0	cassette	0.195
183	ENSG000001	ENSG000001	ISL2	chr15	+	ENSG000001	ENSG000001	TRUE	afe^4|multi_	FALSE	A_C2	chr15:76338	FALSE	FALSE	TRUE	0.004	0.278	0.258	1	0	cassette	0.258
184	ENSG000001	ENSG000001	KALRN	chr3	+	ENSG000001	ENSG000001	TRUE	cassette^2|a	TRUE	C1_A	chr3:124700	FALSE	FALSE	TRUE	0.006	0.141	0.116	0.994	0.003	cassette	0.116
185	ENSG000001	ENSG000001	KALRN	chr3	+	ENSG000001	ENSG000001	TRUE	cassette^2|a	TRUE	C1_A	chr3:124700	FALSE	TRUE	TRUE	0.014	0.308	0.289	1	0	cassette	0.289
186	ENSG000001	ENSG000001	WDR4	chr21	-	ENSG000001	ENSG000001	TRUE	ir^6|putative	FALSE	Distal	chr21:42843	FALSE	FALSE	TRUE	0.012	0.174	0.149	0.993	0.002	AFE/ALE	0.149
187	ENSG000001	ENSG000001	LSS	chr21	-	ENSG000001	ENSG000001	TRUE	putative_alt3	TRUE	Distal	chr21:46193	FALSE	FALSE	TRUE	0.028	0.288	0.264	1	0	AFE/ALE	0.264
188	ENSG000001	ENSG000001	LSS	chr21	-	ENSG000001	ENSG000001	TRUE	cassette^1|ir^2|multi_ex		C1_A	chr21:	FALSE	FALSE	TRUE	0.031	0.145	0.12	0.985	0.007	cassette	0.12
189	ENSG000001	ENSG000001	TAOK1	chr17	+	ENSG000001	ENSG000001	FALSE	cassette^1	TRUE	C1_A	chr17:29534	FALSE	FALSE	TRUE	0	0.166	0.149	1	0	cassette	0.149
190	ENSG000001	ENSG000001	CELF5	chr19	+	ENSG000001	ENSG000001	TRUE	cassette^5|a	TRUE	C1_A	chr19:32249	FALSE	FALSE	TRUE	0.003	0.146	0.121	0.999	0.001	cassette	0.121
191	ENSG000001	ENSG000001	CELF5	chr19	+	ENSG000001	ENSG000001	TRUE	cassette^5|a	TRUE	C1_A	chr19:32249	FALSE	FALSE	TRUE	0.001	0.173	0.149	1	0	cassette	0.149
192	ENSG000001	ENSG000001	CELF5	chr19	+	ENSG000001	ENSG000001	TRUE	cassette^5|a	TRUE	C1_A	chr19:32256	FALSE	FALSE	TRUE	0.001	0.231	0.201	1	0	cassette	0.201
193	ENSG000001	ENSG000001	CELF5	chr19	+	ENSG000001	ENSG000001	TRUE	cassette^5|a	TRUE	C1_A	chr19:32256	FALSE	FALSE	TRUE	0.009	0.347	0.329	1	0	cassette	0.329
194	ENSG000001	ENSG000001	CELF5	chr19	+	ENSG000001	ENSG000001	TRUE	cassette^2|a	TRUE	E1_E2_J1	chr19:32781	FALSE	FALSE	TRUE	0	0.149	0.123	0.999	0	cassette	0.123
195	ENSG000001	ENSG000001	CELF5	chr19	+	ENSG000001	ENSG000001	TRUE	cassette^2|a	TRUE	C1_A	chr19:32781	FALSE	FALSE	TRUE	0	0.16	0.14	1	0	cassette	0.14
196	ENSG000001	ENSG000001	CELF5	chr19	+	ENSG000001	ENSG000001	TRUE	cassette^2|a	TRUE	C1_A	chr19:32781	FALSE	FALSE	TRUE	0	0.163	0.141	1	0	cassette	0.141
197	ENSG000001	ENSG000001	TAL1	chr1	-	ENSG000001	ENSG000001	FALSE	afe^1	TRUE	Distal	chr1:472240	FALSE	TRUE	TRUE	0.021	0.27	0.247	1	0	AFE/ALE	0.247
198	ENSG000001	ENSG000001	USP24	chr1	-	ENSG000001	ENSG000001	FALSE	cassette^1	TRUE	C1_C2	chr1:550786	FALSE	TRUE	TRUE	0.017	0.238	-0.214	1	0	skipping	0.214
199	ENSG000001	ENSG000001	HFM1	chr1	-	ENSG000001	ENSG000001	TRUE	cassette^1|a	TRUE	E1_E2_J1	chr1:913874	FALSE	FALSE	FALSE	0.001	0.232	0.185	0.93	0.011	cassette	0.185
200	ENSG000001	ENSG000001	HFM1	chr1	-	ENSG000001	ENSG000001	TRUE	cassette^1|a	TRUE	E1_E2_J1	chr1:913875	FALSE	FALSE	TRUE	0.001	0.196	0.172	1	0	cassette	0.172
201	ENSG000001	ENSG000001	HFM1	chr1	-	ENSG000001	ENSG000001	TRUE	cassette^1|alt3^20|alt5^		C1_A	chr1:	FALSE	FALSE	TRUE	0.001	0.237	0.211	1	0	cassette	0.211
202	ENSG000001	ENSG000001	HFM1	chr1	-	ENSG000001	ENSG000001	TRUE	cassette^1|a	TRUE	Distal	chr1:913873	FALSE	FALSE	TRUE	0.001	0.187	0.163	0.994	0.002	AFE/ALE	0.163
203	ENSG000001	ENSG000001	KCNT2	chr1	-	ENSG000001	ENSG000001	TRUE	cassette^1|p	TRUE	Proximal	chr1:196305	FALSE	FALSE	TRUE	0.004	0.152	0.123	0.966	0.015	AFE/ALE	0.123
204	ENSG000001	ENSG000001	KCNT2	chr1	-	ENSG000001	ENSG000001	TRUE	ir^2|afe^4|r	TRUE	A_C2	chr1:196316	FALSE	FALSE	TRUE	0.011	0.209	0.181	0.999	0	cassette	0.181
205	ENSG000001	ENSG000001	KIF26B	chr1	+	ENSG000001	ENSG000001	TRUE	putative_ale	TRUE	Proximal	chr1:245419	FALSE	TRUE	TRUE	0.002	0.274	0.251	1	0	AFE/ALE	0.251
206	ENSG000001	ENSG000001	SANBR	chr2	+	ENSG000001	ENSG000001	TRUE	cassette^1|i	TRUE	C1_A	chr2:610884	FALSE	FALSE	TRUE	0.006	0.14	0.116	0.965	0.016	cassette	0.116
207	ENSG000001	ENSG000001	ANKRD30BL	chr2	-	ENSG000001	ENSG000001	TRUE	alt5^3|orpha	TRUE	Distal	chr2:132255	FALSE	FALSE	FALSE	0	0.137	0.109	0.938	0.03	AFE/ALE	0.109
208	ENSG000001	ENSG000001	CLASP2	chr3	-	ENSG000001	ENSG000001	TRUE	cassette^2|t	TRUE	C1_A	chr3:335887	FALSE	FALSE	TRUE	0.043	0.203	0.172	1	0	cassette	0.172
209	ENSG000001	ENSG000001	IFT122	chr3	+	ENSG000001	ENSG000001	TRUE	ir^2|ale^2|p	TRUE	Proximal	chr3:129483	FALSE	FALSE	TRUE	0.003	0.366	0.335	1	0	AFE/ALE	0.335
210	ENSG000001	ENSG000001	IFT122	chr3	+	ENSG000001	ENSG000001	TRUE	ir^2|ale^2|p	TRUE	Proximal	chr3:129483	FALSE	FALSE	TRUE	0.003	0.178	0.157	0.994	0.002	AFE/ALE	0.157
211	ENSG000001	ENSG000001	SLC9B2	chr4	-	ENSG000001	ENSG000001	TRUE	cassette^1|a	FALSE	Proximal	chr4:103065	FALSE	FALSE	TRUE	0.007	0.221	0.197	1	0	AFE/ALE	0.197
212	ENSG000001	ENSG000001	MAP9	chr4	-	ENSG000001	ENSG000001	TRUE	cassette^2|a	FALSE	C2_C1	chr4:155375	FALSE	FALSE	TRUE	0.019	0.163	-0.132	0.957	0.015	skipping	0.132
213	ENSG000001	ENSG000001	STARD4	chr5	-	ENSG000001	ENSG000001	FALSE	cassette^1	FALSE	C1_C2	chr5:111501	FALSE	FALSE	TRUE	0.028	0.22	-0.182	0.986	0.003	skipping	0.182
214	ENSG000001	ENSG000001	ADCY1	chr7	+	ENSG000001	ENSG000001	FALSE	cassette^1	TRUE	C1_A	chr7:456227	FALSE	TRUE	TRUE	0.004	0.56	0.548	1	0	cassette	0.548
215	ENSG000001	ENSG000001	INTS8	chr8	+	ENSG000001	ENSG000001	TRUE	cassette^1|i	TRUE	C1_A	chr8:948321	FALSE	FALSE	FALSE	0.001	0.121	0.097	0.912	0.048	cassette	0.097
216	ENSG000001	ENSG000001	SLITRK5	chr13	+	ENSG000001	ENSG000001	TRUE	cassette^1|i	TRUE	C1_A	chr13:87672	FALSE	FALSE	TRUE	0.002	0.136	0.107	0.951	0.024	cassette	0.107
217	ENSG000001	ENSG000001	SLC39A13	chr11	+	ENSG000001	ENSG000001	TRUE	alt5^1|ir^2	TRUE	Proximal	chr11:47414	FALSE	FALSE	FALSE	0.025	0.141	0.107	0.924	0.032	AFE/ALE	0.107
218	ENSG000001	ENSG000001	RIMKLB	chr12	+	ENSG000001	ENSG000001	TRUE	alt5^3	TRUE	Proximal	chr12:87770	FALSE	FALSE	FALSE	0.031	0.192	0.151	0.936	0.014	AFE/ALE	0.151
219	ENSG000001	ENSG000001	ISLR2	chr15	+	ENSG000001	ENSG000001	TRUE	cassette^4|a	TRUE	C2_A	chr15:74130	FALSE	FALSE	TRUE	0.03	0.161	0.143	1	0	cassette	0.143
220	ENSG000001	ENSG000001	LENG8	chr19	+	ENSG000001	ENSG000001	TRUE	cassette^2|p	TRUE	Distal	chr19:54459	FALSE	FALSE	TRUE	0.03	0.138	0.108	0.973	0.013	AFE/ALE	0.108
221	ENSG000001	ENSG000001	HDGFL2	chr19	+	ENSG000001	ENSG000001	TRUE	cassette^1|a	TRUE	C1_A	chr19:44918	FALSE	TRUE	TRUE	0.001	0.744	0.736	1	0	cassette	0.736
222	ENSG000001	ENSG000001	HDGFL2	chr19	+	ENSG000001	ENSG000001	TRUE	cassette^1|a	TRUE	Proximal	chr19:44921	FALSE	FALSE	TRUE	0.001	0.522	0.505	1	0	AFE/ALE	0.505
223	ENSG000001	ENSG000001	SLC43A2	chr17	-	ENSG000001	ENSG000001	TRUE	ir^1|ale^2|a	TRUE	Distal	chr17:15840	FALSE	FALSE	TRUE	0.001	0.391	0.369	1	0	AFE/ALE	0.369
224	ENSG000001	ENSG000001	MGAT5B	chr17	+	ENSG000001	ENSG000001	TRUE	cassette^1|i	TRUE	C1_A	chr17:76933	FALSE	TRUE	TRUE	0.001	0.273	0.241	1	0	cassette	0.241
225	ENSG000001	ENSG000001	ANKS3	chr16	-	ENSG000001	ENSG000001	TRUE	cassette^5|a	FALSE	C1_A	chr16:47141	FALSE	FALSE	FALSE	0.02	0.145	0.109	0.905	0.04	cassette	0.109
226	ENSG000001	ENSG000001	ANKS3	chr16	-	ENSG000001	ENSG000001	TRUE	cassette^5|a	TRUE	C2_A1	chr16:47052	FALSE	FALSE	TRUE	0.024	0.185	0.152	0.977	0.007	cassette	0.152
227	ENSG000001	ENSG000001	SETD5	chr3	+	ENSG000001	ENSG000001	TRUE	cassette^2|a	TRUE	C2_A	chr3:946861	FALSE	FALSE	TRUE	0.007	0.332	0.315	1	0	cassette	0.315

228	ENSG000001	ENSG000001	ATG4B	chr2	+		ENSG000001	ENSG000001	TRUE	cassette^1|i	TRUE	C1_A	chr2:241668	FALSE	TRUE	TRUE	0.001	0.283	0.263	1	0	cassette	0.263
229	ENSG000001	ENSG000001	LINGO1	chr15	-		ENSG000001	ENSG000001	TRUE	afe^1|putati	TRUE	Proximal	chr15:77641	FALSE	TRUE	TRUE	0.002	0.222	0.203	1	0	AFE/ALE	0.203
230	ENSG000001	ENSG000001	LINGO1	chr15	-		ENSG000001	ENSG000001	TRUE	cassette^3|	TRUE	C1_A	chr15:77756	FALSE	FALSE	TRUE	0.002	0.136	0.112	0.994	0.003	cassette	0.112
231	ENSG000001	ENSG000001	ONECUT1	chr15	-		ENSG000001	ENSG000001	TRUE	putative_alt5	TRUE	Distal	chr15:52777	FALSE	FALSE	TRUE	0	0.227	0.207	1	0	AFE/ALE	0.207
232	ENSG000001	ENSG000001	ONECUT1	chr15	-		ENSG000001	ENSG000001	TRUE	putative_alt5^4|ir^7|ale^		A_C2	chr15:	FALSE	FALSE	TRUE	0.006	0.244	0.225	1	0	cassette	0.225
233	ENSG000001	ENSG000001	DLGAP1	chr18	-		ENSG000001	ENSG000001	FALSE	putative_ale	TRUE	Proximal	chr18:41476	FALSE	TRUE	TRUE	0.004	0.214	0.185	0.998	0	AFE/ALE	0.185
234	ENSG000001	ENSG000001	INSR	chr19	-		ENSG000001	ENSG000001	TRUE	ir^2|putative	TRUE	Proximal	chr19:71698	FALSE	TRUE	TRUE	0.004	0.432	0.407	1	0	AFE/ALE	0.407
235	ENSG000001	ENSG000001	KNDC1	chr10	+		ENSG000001	ENSG000001	TRUE	ir^3|putative	TRUE	Proximal	chr10:13320	FALSE	TRUE	TRUE	0.002	0.254	0.226	1	0	AFE/ALE	0.226
236	ENSG000001	ENSG000001	TRAPPC12	chr2	+		ENSG000001	ENSG000001	TRUE	cassette^1|i	TRUE	C1_A	chr2:345769	FALSE	FALSE	TRUE	0.001	0.201	0.174	0.998	0.001	cassette	0.174
237	ENSG000001	ENSG000001	TRAPPC12	chr2	+		ENSG000001	ENSG000001	TRUE	cassette^1|i	FALSE	Proximal	chr2:345769	FALSE	FALSE	TRUE	0.028	0.369	0.339	1	0	AFE/ALE	0.339
238	ENSG000001	ENSG000001	B3GALT1	chr2	+		ENSG000001	ENSG000001	TRUE	alt5^1|putat	TRUE	Proximal	chr2:167868	FALSE	FALSE	TRUE	0.005	0.146	0.123	0.991	0.004	AFE/ALE	0.123
239	ENSG000001	ENSG000001	CRLF3	chr17	-		ENSG000001	ENSG000001	TRUE	ir^5|putative	TRUE	Proximal	chr17:30786	FALSE	FALSE	FALSE	0.022	0.153	0.117	0.929	0.026	AFE/ALE	0.117
240	ENSG000001	ENSG000001	RIMS2	chr8	+		ENSG000001	ENSG000001	TRUE	cassette^3|	FALSE	C2_A	chr8:104094	FALSE	FALSE	TRUE	0.035	0.18	0.144	0.98	0.007	cassette	0.144
241	ENSG000001	ENSG000001	CHID1	chr11	-		ENSG000001	ENSG000001	FALSE	alt5^1	FALSE	Proximal	chr11:90098	FALSE	TRUE	TRUE	0.014	0.458	0.449	1	0	AFE/ALE	0.449
242	ENSG000001	ENSG000001	CDH4	chr20	+		ENSG000001	ENSG000001	TRUE	putative_ale	TRUE	Proximal	chr20:61254	FALSE	TRUE	TRUE	0.003	0.253	0.226	1	0	AFE/ALE	0.226
243	ENSG000001	ENSG000001	EHMT1	chr9	+		ENSG000001	ENSG000001	TRUE	cassette^2|i	FALSE	C1_A	chr9:137778	FALSE	FALSE	TRUE	0.004	0.153	0.127	0.993	0.003	cassette	0.127
244	ENSG000001	ENSG000001	ADGRB1	chr8	+		ENSG000001	ENSG000001	TRUE	cassette^1|	TRUE	Proximal	chr8:142529	FALSE	FALSE	FALSE	0.005	0.122	0.096	0.93	0.037	AFE/ALE	0.096
245	ENSG000001	ENSG000001	ADGRB1	chr8	+		ENSG000001	ENSG000001	TRUE	cassette^1|	TRUE	Proximal	chr8:142529	FALSE	TRUE	TRUE	0.005	0.259	0.237	1	0	AFE/ALE	0.237
246	ENSG000001	ENSG000001	KCNIP1	chr5	+		ENSG000001	ENSG000001	TRUE	cassette^1|	FALSE	C2_A	chr5:170669	FALSE	FALSE	TRUE	0.003	0.204	0.172	0.997	0.001	cassette	0.172
247	ENSG000001	ENSG000001	PLCXD1	chrY	+		ENSG000001	ENSG000001	TRUE	ale^1|afe^1	TRUE	Proximal	chrY:281684	FALSE	TRUE	TRUE	0.005	0.262	0.238	1	0	AFE/ALE	0.238
248	ENSG000001	ENSG000001	EP400	chr12	+		ENSG000001	ENSG000001	TRUE	cassette^1|i	TRUE	C1_C2	chr12:13199	FALSE	TRUE	TRUE	0.006	0.216	-0.195	1	0	skipping	0.195
249	ENSG000001	ENSG000001	PCBP3	chr21	+		ENSG000001	ENSG000001	TRUE	cassette^2|i	TRUE	Proximal	chr21:45755	FALSE	TRUE	TRUE	0.027	0.609	0.581	1	0	AFE/ALE	0.581
250	ENSG000001	ENSG000001	WDR27	chr6	-		ENSG000001	ENSG000001	TRUE	cassette^2	FALSE	C2_C1	chr6:169658	FALSE	FALSE	TRUE	0.045	0.22	-0.17	0.99	0.003	skipping	0.17
251	ENSG000001	ENSG000001	SLC24A3	chr20	+		ENSG000001	ENSG000001	TRUE	cassette^2|	TRUE	C1_A	chr20:19681	FALSE	TRUE	TRUE	0.029	0.356	0.309	1	0	cassette	0.309
252	ENSG000001	ENSG000001	ARL15	chr5	-		ENSG000001	ENSG000001	TRUE	ale^2|putati	TRUE	Distal	chr5:542853	FALSE	FALSE	TRUE	0.025	0.183	0.156	0.965	0.008	AFE/ALE	0.156
253	ENSG000001	ENSG000001	ARL15	chr5	-		ENSG000001	ENSG000001	TRUE	ale^2|putati	TRUE	Proximal	chr5:541719	FALSE	FALSE	FALSE	0.002	0.142	0.113	0.926	0.034	AFE/ALE	0.113
254	ENSG000001	ENSG000001	ADARB2	chr10	-		ENSG000001	ENSG000001	TRUE	cassette^1|	TRUE	C1_A	chr10:13762	FALSE	TRUE	TRUE	0.002	0.61	0.603	1	0	cassette	0.603
255	ENSG000001	ENSG000001	ADARB2	chr10	-		ENSG000001	ENSG000001	TRUE	alt3^7|putat	TRUE	Proximal	chr10:16459	FALSE	FALSE	TRUE	0.015	0.208	0.186	1	0	AFE/ALE	0.186
256	ENSG000001	ENSG000001	GNB1L	chr22	-		ENSG000001	ENSG000001	TRUE	cassette^1|	TRUE	Proximal	chr22:19824	FALSE	FALSE	TRUE	0.003	0.538	0.523	1	0	AFE/ALE	0.523
257	ENSG000001	ENSG000001	RASA3	chr13	-		ENSG000001	ENSG000001	TRUE	putative_ale	TRUE	Proximal	chr13:11399	FALSE	TRUE	TRUE	0.001	0.261	0.237	1	0	AFE/ALE	0.237
258	ENSG000001	ENSG000001	ZNF529	chr19	-		ENSG000001	ENSG000001	TRUE	cassette^1|	TRUE	Proximal	chr19:36572	FALSE	TRUE	TRUE	0.011	0.39	0.362	1	0	AFE/ALE	0.362
259	ENSG000001	ENSG000001	ZFP91	chr11	+		ENSG000001	ENSG000001	FALSE	cassette^1	TRUE	C1_A	chr11:58616	FALSE	FALSE	TRUE	0.001	0.21	0.19	1	0	cassette	0.19
260	ENSG000001	ENSG000001	ZSCAN30	chr18	-		ENSG000001	ENSG000001	TRUE	alt3^1|ale^2	TRUE	Distal	chr18:35254	FALSE	FALSE	FALSE	0.045	0.202	0.145	0.929	0.017	AFE/ALE	0.145
261	ENSG000001	ENSG000001	MAPK12	chr22	-		ENSG000001	ENSG000001	FALSE	alt5^1	TRUE	Distal	chr22:50248	FALSE	FALSE	TRUE	0.046	0.235	0.184	0.994	0.001	AFE/ALE	0.184
262	ENSG000001	ENSG000001	AGRN	chr1	+		ENSG000001	ENSG000001	TRUE	ale^1|afe^1	TRUE	Proximal	chr1:102246	FALSE	TRUE	TRUE	0.009	0.186	0.165	1	0	AFE/ALE	0.165
263	ENSG000001	ENSG000001	RALGAPA2	chr20	-		ENSG000001	ENSG000001	FALSE	cassette^1	TRUE	C1_A	chr20:20491	FALSE	TRUE	TRUE	0.004	0.416	0.398	1	0	cassette	0.398
264	ENSG000001	ENSG000001	GREB1	chr2	+		ENSG000001	ENSG000001	TRUE	cassette^2|	TRUE	C1_A	chr2:115808	FALSE	FALSE	TRUE	0.011	0.18	0.159	1	0	cassette	0.159
265	ENSG000001	ENSG000001	GREB1	chr2	+		ENSG000001	ENSG000001	TRUE	cassette^2|	TRUE	Proximal	chr2:114833	FALSE	FALSE	FALSE	0.012	0.117	0.096	0.927	0.04	AFE/ALE	0.096
266	ENSG000001	ENSG000001	ELAVL3	chr19	-		ENSG000001	ENSG000001	FALSE	cassette^1	TRUE	C1_A	chr19:11463	FALSE	FALSE	TRUE	0	0.141	0.115	0.993	0.003	cassette	0.115
267	ENSG000001	ENSG000001	TRRAP	chr7	+		ENSG000001	ENSG000001	TRUE	cassette^1|i	TRUE	C1_A	chr7:988812	FALSE	FALSE	TRUE	0	0.149	0.126	1	0	cassette	0.126
268	ENSG000001	ENSG000001	ARMCX4	chrX	+		ENSG000001	ENSG000001	FALSE	ale^1	FALSE	Distal	chrX:101488	FALSE	FALSE	FALSE	0.026	0.183	0.14	0.911	0.019	AFE/ALE	0.14
269	ENSG000001	ENSG000001	NCOR2	chr12	-		ENSG000001	ENSG000001	TRUE	cassette^1|	TRUE	C1_A	chr12:12447	FALSE	FALSE	TRUE	0.001	0.129	0.106	0.966	0.017	cassette	0.106
270	ENSG000001	ENSG000001	NCOR2	chr12	-		ENSG000001	ENSG000001	TRUE	cassette^1|	TRUE	Distal	chr12:12447	FALSE	FALSE	TRUE	0.005	0.169	0.147	1	0	AFE/ALE	0.147
271	ENSG000001	ENSG000001	MYO18A	chr17	-		ENSG000001	ENSG000001	TRUE	cassette^4|	TRUE	C1_A	chr17:29131	FALSE	FALSE	TRUE	0.001	0.371	0.361	1	0	cassette	0.361
272	ENSG000001	ENSG000001	MYO18A	chr17	-		ENSG000001	ENSG000001	TRUE	cassette^4|	TRUE	Distal	chr17:29124	FALSE	FALSE	TRUE	0.004	0.536	0.528	1	0	AFE/ALE	0.528
273	ENSG000001	ENSG000001	NF1	chr17	+		ENSG000001	ENSG000001	FALSE	cassette^1	FALSE	C1_A	chr17:31249	FALSE	FALSE	FALSE	0.026	0.124	0.096	0.925	0.04	cassette	0.096
274	ENSG000001	ENSG000001	DAPK1	chr9	+		ENSG000001	ENSG000001	TRUE	cassette^1|	TRUE	C1_A	chr9:874991	FALSE	FALSE	TRUE	0.001	0.147	0.121	0.998	0.001	cassette	0.121
275	ENSG000001	ENSG000001	DAPK1	chr9	+		ENSG000001	ENSG000001	TRUE	cassette^1|	FALSE	Proximal	chr9:874991	FALSE	FALSE	TRUE	0.046	0.222	0.176	1	0	AFE/ALE	0.176
276	ENSG000001	ENSG000001	RGPD4	chr2	+		ENSG000001	ENSG000001	FALSE	putative_afe	TRUE	Proximal	chr2:107878	FALSE	TRUE	TRUE	0.007	0.274	0.254	1	0	AFE/ALE	0.254
277	ENSG000001	ENSG000001	ZGPAT	chr20	+		ENSG000001	ENSG000001	FALSE	cassette^1	TRUE	C1_A	chr20:63709	FALSE	TRUE	TRUE	0.005	0.433	0.414	1	0	cassette	0.414
278	ENSG000001	ENSG000001	PHF2	chr9	+		ENSG000001	ENSG000001	TRUE	cassette^2|t	TRUE	Proximal	chr9:936605	FALSE	TRUE	TRUE	0	0.495	0.481	1	0	AFE/ALE	0.481
279	ENSG000001	ENSG000001	MYO1C	chr17	-		ENSG000001	ENSG000001	FALSE	cassette^1	TRUE	C1_C2	chr17:14722	FALSE	TRUE	TRUE	0.006	0.234	-0.204	1	0	skipping	0.204
280	ENSG000001	ENSG000001	SDAD1	chr4	-		ENSG000001	ENSG000001	TRUE	cassette^1|	TRUE	C1_A	chr4:759848	FALSE	FALSE	TRUE	0.001	0.15	0.123	0.99	0.004	cassette	0.123
281	ENSG000001	ENSG000001	UVRAG	chr11	+		ENSG000001	ENSG000001	TRUE	cassette^1|i	FALSE	C1_A	chr11:76008	FALSE	FALSE	TRUE	0.031	0.181	0.139	0.951	0.014	cassette	0.139
282	ENSG000001	ENSG000001	RYR2	chr1	+		ENSG000001	ENSG000001	TRUE	alt3^1|ale^2	TRUE	Distal	chr1:237585	FALSE	FALSE	TRUE	0.012	0.294	0.246	0.986	0.002	AFE/ALE	0.246
283	ENSG000001	ENSG000001	CEP290	chr12	-		ENSG000001	ENSG000001	FALSE	alt3^1	TRUE	Proximal	chr12:88086	FALSE	TRUE	TRUE	0.004	0.469	0.455	1	0	AFE/ALE	0.455
284	ENSG000001	ENSG000001	CEP290	chr12	-		ENSG000001	ENSG000001	FALSE	cassette^1	FALSE	C2_C1	chr12:88111	FALSE	FALSE	FALSE	0.046	0.185	-0.128	0.922	0.027	skipping	0.128
285	ENSG000001	ENSG000001	UNC13B	chr9	+		ENSG000001	ENSG000001	FALSE	cassette^1	FALSE	C1_A	chr9:353139	FALSE	TRUE	TRUE	0.044	0.419	0.377	1	0	cassette	0.377
286	ENSG000001	ENSG000001	COLGALT2	chr1	-		ENSG000001	ENSG000001	FALSE	alt5^1	TRUE	Distal	chr1:183978	FALSE	FALSE	FALSE	0.02	0.152	0.12	0.939	0.024	AFE/ALE	0.12
287	ENSG000001	ENSG000001	SCAMP5	chr15	+		ENSG000001	ENSG000001	TRUE	cassette^7|	TRUE	C1_A	chr15:75007	FALSE	FALSE	TRUE	0.036	0.354	0.323	1	0	cassette	0.323
288	ENSG000001	ENSG000001	SCAMP5	chr15	+		ENSG000001	ENSG000001	TRUE	cassette^7|	FALSE	C2_A	chr15:75009	FALSE	FALSE	TRUE	0.047	0.203	0.167	1	0	cassette	0.167
289	ENSG000001	ENSG000001	ZNF521	chr18	-		ENSG000001	ENSG000001	TRUE	putative_ale	TRUE	Proximal	chr18:25160	FALSE	FALSE	TRUE	0.001	0.162	0.138	0.996	0.002	AFE/ALE	0.138
290	ENSG000001	ENSG000001	ZNF521	chr18	-		ENSG000001	ENSG000001	TRUE	putative_ale	TRUE	Proximal	chr18:25162	FALSE	FALSE	TRUE	0.008	0.2	0.177	1	0	AFE/ALE	0.177
291	ENSG000001	ENSG000001	KIAA0753	chr17	-		ENSG000001	ENSG000001	FALSE	cassette^1	TRUE	C1_A	chr17:65809	FALSE	TRUE	TRUE	0.004	0.483	0.461	1	0	cassette	0.461
292	ENSG000002	ENSG000002	LINC00963	chr9	+		ENSG000002	ENSG000002	TRUE	cassette^2|	FALSE	A_C2	chr9:129503	FALSE	FALSE	TRUE	0.014	0.144	0.117	0.98	0.009	cassette	0.117
293	ENSG000002	ENSG000002	STARD7-AS1	chr2	+		ENSG000002	ENSG000002	TRUE	cassette^1|	FALSE	Distal	chr2:962089	FALSE	FALSE	TRUE	0.042	0.253	0.193	0.958	0.006	AFE/ALE	0.193
294	ENSG000002	ENSG000002	PCDHA2	chr5	+		ENSG000002	ENSG000002	TRUE	alt3^1|alt3_	TRUE	E1_E2_J2	chr5:140796	FALSE	FALSE	TRUE	0.003	0.156	0.134	1	0	cassette	0.134
295	ENSG000002	ENSG000002	LIN52	chr14	+		ENSG000002	ENSG000002	FALSE	ale^1	FALSE	Proximal	chr14:74101	FALSE	FALSE	TRUE	0.018	0.181	0.152	0.988	0.004	AFE/ALE	0.152
296	ENSG000002	ENSG000002	NYNRIN	chr14	+		ENSG000002	ENSG000002	TRUE	cassette^1|i	TRUE	C1_A	chr14:24411	FALSE	FALSE	FALSE	0.009	0.151	0.122	0.946	0.021	cassette	0.122
297	ENSG000002	ENSG000002	ZDHHC11B	chr5	-		ENSG000002	ENSG000002	TRUE	cassette^1|i	TRUE	Proximal	chr5:733156	FALSE	FALSE	TRUE	0.002	0.32	0.299	1	0	AFE/ALE	0.299
298	ENSG000002	ENSG000002	ANKRD28	chr3	-		ENSG000002	ENSG000002	TRUE	cassette^3|	TRUE	E2_E1_J2	chr3:156673	FALSE	FALSE	TRUE	0	0.296	0.27	1	0	cassette	0.27
299	ENSG000002	ENSG000002	ANKRD28	chr3	-		ENSG000002	ENSG000002	TRUE	cassette^3|	TRUE	E2_E1_J1	chr3:156715	FALSE	FALSE	TRUE	0	0.174	0.152	0.997	0.001	cassette	0.152
300	ENSG000002	ENSG000002	ANKRD28	chr3	-		ENSG000002	ENSG000002	TRUE	cassette^3|	TRUE	E2_E1_J1	chr3:156764	FALSE	FALSE	TRUE	0.009	0.266	0.24	0.975	0.004	cassette	0.24
301	ENSG000002	ENSG000002	ANKRD28	chr3	-		ENSG000002	ENSG000002	TRUE	cassette^3|	FALSE	A_C2	chr3:156770	FALSE	FALSE	TRUE	0.001	0.219	0.192	0.991	0.002	cassette	0.192
302	ENSG000002	ENSG000002	ANKRD28	chr3	-		ENSG000002	ENSG000002	TRUE	cassette^3|	TRUE	E2_E1_J1	chr3:157131	FALSE	FALSE	TRUE	0	0.304	0.284	1	0	cassette	0.284
303	ENSG000002	ENSG000002	ANKRD28	chr3	-		ENSG000002	ENSG000002	TRUE	cassette^3|	TRUE	Distal	chr3:157062	FALSE	TRUE	TRUE	0.005	0.438	0.393	1	0	AFE/ALE	0.393

304	ENSG000002	ENSG000002	DENND1B	chr1	-	ENSG000002	ENSG000002	FALSE	putative_ale	TRUE	Proximal	chr1:197560	FALSE	FALSE	FALSE	0.023	0.159	0.129	0.944	0.019	AFE/ALE	0.129
305	ENSG000002	ENSG000002	RPS29	chr14	-	ENSG000002	ENSG000002	TRUE	alt3^162|ale	TRUE	Proximal	chr14:49583	FALSE	FALSE	FALSE	0	0.151	0.122	0.923	0.026	AFE/ALE	0.122
306	ENSG000002	ENSG000002	RPS29	chr14	-	ENSG000002	ENSG000002	TRUE	alt3^162|ale	TRUE	Proximal	chr14:49585	FALSE	FALSE	FALSE	0	0.136	0.11	0.944	0.027	AFE/ALE	0.11
307	ENSG000002	ENSG000002	RPS29	chr14	-	ENSG000002	ENSG000002	TRUE	alt3^162|ale	TRUE	Distal	chr14:49573	FALSE	FALSE	TRUE	0	0.657	0.646	1	0	AFE/ALE	0.646
308	ENSG000002	ENSG000002	RPS29	chr14	-	ENSG000002	ENSG000002	TRUE	alt3^162|ale	TRUE	Distal	chr14:49585	FALSE	FALSE	TRUE	0	0.212	0.189	1	0	AFE/ALE	0.189
309	ENSG000002	ENSG000002	SEPTIN7P2	chr7	-	ENSG000002	ENSG000002	TRUE	cassette^1|	TRUE	C1_A	chr7:457356	FALSE	FALSE	TRUE	0.029	0.356	0.328	1	0	cassette	0.328
310	ENSG000002	ENSG000002	SEPTIN7P2	chr7	-	ENSG000002	ENSG000002	TRUE	cassette^1|	TRUE	Distal	chr7:457283	FALSE	FALSE	TRUE	0.006	0.281	0.262	1	0	AFE/ALE	0.262
311	ENSG000002	ENSG000002	NPEPL1	chr20	+	ENSG000002	ENSG000002	TRUE	ir^2|putative	TRUE	Proximal	chr20:58694	FALSE	TRUE	TRUE	0.001	0.247	0.22	1	0	AFE/ALE	0.22
312	ENSG000002	ENSG000002	LINC00863	chr10	+	ENSG000002	ENSG000002	TRUE	cassette^1|i	FALSE	A_C2	chr10:87354	FALSE	FALSE	TRUE	0.018	0.212	0.173	0.963	0.006	cassette	0.173
313	ENSG000002	ENSG000002	FAM66C	chr12	+	ENSG000002	ENSG000002	TRUE	cassette^3|	TRUE	C1_A	chr12:81885	FALSE	TRUE	TRUE	0.015	0.447	0.436	1	0	cassette	0.436
314	ENSG000002	ENSG000002	FAM66C	chr12	+	ENSG000002	ENSG000002	TRUE	cassette^3|	FALSE	C1_A	chr12:81886	FALSE	TRUE	TRUE	0.034	0.383	0.357	1	0	cassette	0.357
315	ENSG000002	ENSG000002	CROCCP4	chr1	+	ENSG000002	ENSG000002	TRUE	alt3^1|putat	TRUE	Distal	chr1:167406	FALSE	FALSE	TRUE	0.001	0.21	0.186	1	0	AFE/ALE	0.186
316	ENSG000002	ENSG000002	ZNF826P	chr19	-	ENSG000002	ENSG000002	TRUE	cassette^2|	FALSE	C1_A	chr19:20409	FALSE	FALSE	TRUE	0.045	0.642	0.611	1	0	cassette	0.611
317	ENSG000002	ENSG000002	ZNF826P	chr19	-	ENSG000002	ENSG000002	TRUE	cassette^2|	FALSE	Proximal	chr19:20409	FALSE	FALSE	TRUE	0.023	0.522	0.508	1	0	AFE/ALE	0.508
318	ENSG000002	ENSG000002	LINC01122	chr2	+	ENSG000002	ENSG000002	TRUE	cassette^2|	FALSE	Distal	chr2:586567	FALSE	FALSE	FALSE	0.028	0.184	0.147	0.947	0.014	AFE/ALE	0.147
319	ENSG000002	ENSG000002	RPL23AP7	chr2	-	ENSG000002	ENSG000002	TRUE	alt5^3|ir^3	TRUE	Proximal	chr2:113625	FALSE	FALSE	TRUE	0.038	0.241	0.197	0.999	0	AFE/ALE	0.197
320	ENSG000002	ENSG000002	PEDS1	chr20	-	ENSG000002	ENSG000002	FALSE	cassette^1	FALSE	C1_C2	chr20:50129	FALSE	FALSE	TRUE	0.033	0.262	-0.23	1	0	skipping	0.23
321	ENSG000002	ENSG000002	ZNF286B	chr17	-	ENSG000002	ENSG000002	TRUE	cassette^1|i	TRUE	C1_C2	chr17:18680	FALSE	FALSE	FALSE	0.027	0.14	-0.11	0.921	0.034	skipping	0.11
322	ENSG000002	ENSG000002	LINC02506	chr4	+	ENSG000002	ENSG000002	TRUE	cassette^6|i	TRUE	C2_A	chr4:321571	FALSE	FALSE	TRUE	0.031	0.146	0.121	0.987	0.006	cassette	0.121
323	ENSG000002	ENSG000002	ENSG000002	chr9	+	ENSG000002	ENSG000002	FALSE	cassette^1	TRUE	C1_A	chr9:128952	FALSE	TRUE	TRUE	0.003	0.783	0.781	1	0	cassette	0.781
324	ENSG000002	ENSG000002	STX16-NPEPL	chr20	+	ENSG000002	ENSG000002	TRUE	ir^2|putative	TRUE	Proximal	chr20:58694	FALSE	TRUE	TRUE	0.001	0.248	0.224	1	0	AFE/ALE	0.224
325	ENSG000002	ENSG000002	FAM66D	chr8	+	ENSG000002	ENSG000002	TRUE	cassette^1|	TRUE	C1_A	chr8:121220	FALSE	FALSE	TRUE	0.021	0.594	0.584	1	0	cassette	0.584
326	ENSG000002	ENSG000002	ZFP91-CNTF	chr11	+	ENSG000002	ENSG000002	FALSE	cassette^1	TRUE	C1_A	chr11:58616	FALSE	FALSE	TRUE	0.001	0.21	0.19	1	0	cassette	0.19
327	ENSG000002	ENSG000002	ENSG000002	chr15	+	ENSG000002	ENSG000002	TRUE	orphan_junc	TRUE	Orphan	chr15:67840	FALSE	FALSE	TRUE	0.001	0.209	0.181	0.994	0.002	cassette	0.181
328	ENSG000002	ENSG000002	ENSG000002	chr15	+	ENSG000002	ENSG000002	TRUE	orphan_junc	TRUE	Orphan	chr15:67840	FALSE	FALSE	TRUE	0.02	0.198	0.173	0.987	0.003	cassette	0.173
329	ENSG000002	ENSG000002	CORO7	chr16	-	ENSG000002	ENSG000002	FALSE	putative_ale	TRUE	Proximal	chr16:43674	FALSE	TRUE	TRUE	0.019	0.347	0.323	1	0	AFE/ALE	0.323
330	ENSG000002	ENSG000002	SUZ12P1	chr17	+	ENSG000002	ENSG000002	TRUE	putative_alt3	TRUE	Proximal	chr17:30786	FALSE	FALSE	FALSE	0.021	0.146	0.112	0.922	0.031	AFE/ALE	0.112
331	ENSG000002	ENSG000002	ENSG000002	chr19	-	ENSG000002	ENSG000002	TRUE	alt5^3	TRUE	Distal	chr19:43900	FALSE	FALSE	TRUE	0.047	0.219	0.167	0.962	0.008	AFE/ALE	0.167
332	ENSG000002	ENSG000002	ENSG000002	chr20	+	ENSG000002	ENSG000002	FALSE	cassette^1	TRUE	C1_A	chr20:63709	FALSE	TRUE	TRUE	0.005	0.433	0.414	1	0	cassette	0.414
333	ENSG000002	ENSG000002	SEC22B4P	chr1	-	ENSG000002	ENSG000002	TRUE	alt5^2|alt3_	TRUE	E1_E2_J1	chr1:146376	FALSE	FALSE	TRUE	0.002	0.165	0.145	0.981	0.007	cassette	0.145
334	ENSG000002	ENSG000002	SEC22B4P	chr1	-	ENSG000002	ENSG000002	TRUE	alt5^2|alt3_	TRUE	E1_E2_J2	chr1:146376	FALSE	FALSE	FALSE	0.002	0.136	0.114	0.94	0.028	cassette	0.114
335	ENSG000002	ENSG000002	ENSG000002	chr21	+	ENSG000002	ENSG000002	TRUE	cassette^127|alt3^84|alt		C1_A	chr21:	FALSE	FALSE	TRUE	0.048	0.295	0.248	1	0	cassette	0.248
336	ENSG000002	ENSG000002	ENSG000002	chr21	+	ENSG000002	ENSG000002	TRUE	cassette^127|alt3^84|alt		C1_A	chr21:	FALSE	FALSE	FALSE	0	0.125	0.101	0.928	0.037	cassette	0.101
337	ENSG000002	ENSG000002	ENSG000002	chr21	+	ENSG000002	ENSG000002	TRUE	cassette^127|alt3^84|alt		C1_A	chr21:	FALSE	FALSE	TRUE	0	0.161	0.134	0.983	0.007	cassette	0.134
338	ENSG000002	ENSG000002	ENSG000002	chr21	+	ENSG000002	ENSG000002	TRUE	cassette^127|alt3^84|alt		C1_A	chr21:	FALSE	TRUE	TRUE	0.004	0.676	0.667	1	0	cassette	0.667
339	ENSG000002	ENSG000002	ENSG000002	chr21	+	ENSG000002	ENSG000002	TRUE	cassette^127|alt3^84|alt		C1_A	chr21:	FALSE	FALSE	TRUE	0.001	0.174	0.149	0.976	0.008	cassette	0.149
340	ENSG000002	ENSG000002	ENSG000002	chr21	+	ENSG000002	ENSG000002	TRUE	cassette^127|alt3^84|alt		C1_A	chr21:	FALSE	FALSE	TRUE	0.004	0.166	0.144	0.975	0.009	cassette	0.144
341	ENSG000002	ENSG000002	ENSG000002	chr21	+	ENSG000002	ENSG000002	TRUE	cassette^127|alt3^84|alt		C1_A	chr21:	FALSE	FALSE	TRUE	0.006	0.336	0.317	1	0	cassette	0.317
342	ENSG000002	ENSG000002	ENSG000002	chr21	+	ENSG000002	ENSG000002	TRUE	cassette^127|alt3^84|alt		C1_A	chr21:	FALSE	FALSE	TRUE	0	0.462	0.464	1	0	cassette	0.464
343	ENSG000002	ENSG000002	ENSG000002	chr21	+	ENSG000002	ENSG000002	TRUE	cassette^12	TRUE	E1_E2_J1	chr21:82168	FALSE	FALSE	TRUE	0.03	0.207	0.184	1	0	cassette	0.184
344	ENSG000002	ENSG000002	ENSG000002	chr21	+	ENSG000002	ENSG000002	TRUE	cassette^127|alt3^84|alt		C1_A	chr21:	FALSE	FALSE	TRUE	0.001	0.196	0.173	0.981	0.005	cassette	0.173
345	ENSG000002	ENSG000002	ENSG000002	chr21	+	ENSG000002	ENSG000002	TRUE	cassette^12	TRUE	Distal	chr21:82058	FALSE	FALSE	TRUE	0.001	0.204	0.178	0.989	0.003	AFE/ALE	0.178
346	ENSG000002	ENSG000002	ENSG000002	chr21	+	ENSG000002	ENSG000002	TRUE	cassette^12	TRUE	C1_A1	chr21:82072	FALSE	FALSE	TRUE	0.015	0.255	0.23	1	0	cassette	0.23
347	ENSG000002	ENSG000002	ENSG000002	chr21	+	ENSG000002	ENSG000002	TRUE	cassette^12	TRUE	Distal	chr21:82078	FALSE	FALSE	FALSE	0	0.157	0.13	0.94	0.024	AFE/ALE	0.13
348	ENSG000002	ENSG000002	ENSG000002	chr21	+	ENSG000002	ENSG000002	TRUE	cassette^12	TRUE	Proximal	chr21:82086	FALSE	FALSE	TRUE	0.003	0.324	0.301	1	0	AFE/ALE	0.301
349	ENSG000002	ENSG000002	ENSG000002	chr21	+	ENSG000002	ENSG000002	TRUE	cassette^12	TRUE	Distal	chr21:82086	FALSE	FALSE	TRUE	0.003	0.304	0.283	1	0	AFE/ALE	0.283
350	ENSG000002	ENSG000002	ENSG000002	chr21	+	ENSG000002	ENSG000002	TRUE	cassette^12	TRUE	C1_A1	chr21:82090	FALSE	FALSE	TRUE	0.003	0.24	0.207	0.977	0.004	cassette	0.207
351	ENSG000002	ENSG000002	ENSG000002	chr21	+	ENSG000002	ENSG000002	TRUE	cassette^12	TRUE	Distal	chr21:82090	FALSE	FALSE	TRUE	0.003	0.201	0.173	0.967	0.008	AFE/ALE	0.173
352	ENSG000002	ENSG000002	ENSG000002	chr21	+	ENSG000002	ENSG000002	TRUE	cassette^12	TRUE	Proximal	chr21:82086	FALSE	FALSE	TRUE	0.001	0.15	0.126	0.956	0.018	AFE/ALE	0.126
353	ENSG000002	ENSG000002	ENSG000002	chr21	+	ENSG000002	ENSG000002	TRUE	cassette^12	TRUE	A_C2	chr21:82005	FALSE	FALSE	FALSE	0.011	0.147	0.124	0.933	0.027	cassette	0.124
354	ENSG000002	ENSG000002	ENSG000002	chr21	+	ENSG000002	ENSG000002	TRUE	cassette^12	TRUE	Proximal	chr21:82011	FALSE	FALSE	TRUE	0.039	0.201	0.158	0.955	0.014	AFE/ALE	0.158
355	ENSG000002	ENSG000002	ENSG000002	chr21	+	ENSG000002	ENSG000002	TRUE	cassette^12	TRUE	Proximal	chr21:82072	FALSE	FALSE	TRUE	0.019	0.206	0.185	1	0	AFE/ALE	0.185
356	ENSG000002	ENSG000002	ENSG000002	chr21	+	ENSG000002	ENSG000002	TRUE	cassette^12	TRUE	Distal	chr21:82101	FALSE	FALSE	TRUE	0.002	0.432	0.419	1	0	AFE/ALE	0.419
357	ENSG000002	ENSG000002	ENSG000002	chr21	+	ENSG000002	ENSG000002	TRUE	cassette^127|alt3^84|alt		A_C2	chr21:	FALSE	FALSE	FALSE	0.021	0.152	0.121	0.915	0.031	cassette	0.121
358	ENSG000002	ENSG000002	ENSG000002	chr21	+	ENSG000002	ENSG000002	TRUE	cassette^12	TRUE	E2_E1_J1	chr21:82120	FALSE	FALSE	TRUE	0	0.165	0.15	0.961	0.013	cassette	0.15
359	ENSG000002	ENSG000002	ENSG000002	chr21	+	ENSG000002	ENSG000002	TRUE	cassette^12	TRUE	Distal	chr21:82039	FALSE	FALSE	FALSE	0.029	0.169	0.149	0.908	0.021	AFE/ALE	0.149
360	ENSG000002	ENSG000002	ENSG000002	chr21	+	ENSG000002	ENSG000002	TRUE	cassette^127|alt3^84|alt		A_C2	chr21:	FALSE	FALSE	TRUE	0.01	0.183	0.148	0.972	0.008	cassette	0.148
361	ENSG000002	ENSG000002	ENSG000002	chr21	+	ENSG000002	ENSG000002	TRUE	cassette^12	TRUE	Distal	chr21:82069	FALSE	FALSE	TRUE	0	0.186	0.162	0.998	0.001	AFE/ALE	0.162
362	ENSG000002	ENSG000002	ENSG000002	chr21	+	ENSG000002	ENSG000002	TRUE	cassette^12	TRUE	C2_A	chr21:82126	FALSE	FALSE	TRUE	0.005	0.46	0.441	1	0	cassette	0.441
363	ENSG000002	ENSG000002	ENSG000002	chr21	+	ENSG000002	ENSG000002	TRUE	cassette^12	TRUE	C2_A2	chr21:82108	FALSE	FALSE	TRUE	0	0.502	0.515	1	0	cassette	0.515
364	ENSG000002	ENSG000002	ENSG000002	chr21	+	ENSG000002	ENSG000002	TRUE	cassette^127|alt3^84|alt		A_C2	chr21:	FALSE	FALSE	TRUE	0.005	0.46	0.441	1	0	cassette	0.441
365	ENSG000002	ENSG000002	ENSG000002	chr21	+	ENSG000002	ENSG000002	TRUE	cassette^12	TRUE	Proximal	chr21:82058	FALSE	FALSE	TRUE	0.003	0.422	0.383	1	0	AFE/ALE	0.383
366	ENSG000002	ENSG000002	ENSG000002	chr21	+	ENSG000002	ENSG000002	TRUE	cassette^127|alt3^84|alt		A_C2	chr21:	FALSE	FALSE	FALSE	0.012	0.14	0.115	0.91	0.04	cassette	0.115
367	ENSG000002	ENSG000002	ENSG000002	chr21	+	ENSG000002	ENSG000002	TRUE	cassette^12	TRUE	C2_A1	chr21:82154	FALSE	FALSE	FALSE	0.002	0.124	0.106	0.908	0.046	cassette	0.106
368	ENSG000002	ENSG000002	ENSG000002	chr21	+	ENSG000002	ENSG000002	TRUE	cassette^127|alt3^84|alt		A_C2	chr21:	FALSE	FALSE	TRUE	0	0.258	0.228	1	0	cassette	0.228
369	ENSG000002	ENSG000002	ENSG000002	chr21	+	ENSG000002	ENSG000002	TRUE	cassette^127|alt3^84|alt		A_C2	chr21:	FALSE	FALSE	TRUE	0	0.223	0.206	1	0	cassette	0.206
370	ENSG000002	ENSG000002	ENSG000002	chr21	+	ENSG000002	ENSG000002	TRUE	cassette^12	TRUE	C2_A	chr21:82146	FALSE	FALSE	TRUE	0.021	0.417	0.383	1	0	cassette	0.383
371	ENSG000002	ENSG000002	ENSG000002	chr21	+	ENSG000002	ENSG000002	TRUE	cassette^127|alt3^84|alt		A_C2	chr21:	FALSE	FALSE	TRUE	0	0.249	0.223	1	0	cassette	0.223
372	ENSG000002	ENSG000002	ENSG000002	chr21	+	ENSG000002	ENSG000002	TRUE	cassette^127|alt3^84|alt		A_C2	chr21:	FALSE	FALSE	FALSE	0.04	0.156	0.119	0.912	0.032	cassette	0.119
373	ENSG000002	ENSG000002	ENSG000002	chr21	+	ENSG000002	ENSG000002	TRUE	cassette^12	TRUE	E2_E1_J2	chr21:82127	FALSE	FALSE	TRUE	0	0.22	0.193	1	0	cassette	0.193
374	ENSG000002	ENSG000002	ENSG000002	chr21	+	ENSG000002	ENSG000002	TRUE	cassette^12	TRUE	Distal	chr21:82072	FALSE	FALSE	TRUE	0.001	0.428	0.421	1	0	AFE/ALE	0.421
375	ENSG000002	ENSG000002	ENSG000002	chr21	+	ENSG000002	ENSG000002	TRUE	cassette^127|alt3^84|alt		A_C2	chr21:	FALSE	FALSE	TRUE	0.029	0.255	0.227	1	0	cassette	0.227
376	ENSG000002	ENSG000002	ENSG000002	chr21	+	ENSG000002	ENSG000002	TRUE	cassette^127|alt3^84|alt		A_C2	chr21:	FALSE	FALSE	TRUE	0.001	0.141	0.117	0.993	0.003	cassette	0.117
377	ENSG000002	ENSG000002	ENSG000002	chr21	-	ENSG000002	ENSG000002	TRUE	cassette^1|i	TRUE	A_C2	chr21:51188	FALSE	FALSE	TRUE	0.002	0.245	0.221	1	0	cassette	0.221
378	ENSG000002	ENSG000002	ENSG000002	chr21	+	ENSG000002	ENSG000002	TRUE	cassette^474|alt3^206|a		C1_A	chr21:	FALSE	FALSE	TRUE	0.001	0.216	0.191	0.999	0	cassette	0.191
379	ENSG000002	ENSG000002	ENSG000002	chr21	+	ENSG000002	ENSG000002	TRUE	cassette^474|alt3^206|a		C1_A	chr21:	FALSE	FALSE	TRUE	0.003	0.22	0.193	1	0	cassette	0.193

380	ENSG000002	ENSG000002	ENSG000002	chr21	+	ENSG000002	ENSG000002	TRUE	cassette^474|alt3^206|a		C1_A	chr21:	FALSE	FALSE	TRUE	0.015	0.162	0.135	0.993	0.003	cassette	0.135
381	ENSG000002	ENSG000002	ENSG000002	chr21	+	ENSG000002	ENSG000002	TRUE	cassette^474|alt3^206|a		C1_A	chr21:	FALSE	FALSE	TRUE	0.001	0.296	0.284	1	0	cassette	0.284
382	ENSG000002	ENSG000002	ENSG000002	chr21	+	ENSG000002	ENSG000002	TRUE	cassette^474|alt3^206|a		C1_A	chr21:	FALSE	FALSE	TRUE	0.006	0.223	0.204	1	0	cassette	0.204
383	ENSG000002	ENSG000002	ENSG000002	chr21	+	ENSG000002	ENSG000002	TRUE	cassette^474|alt3^206|a		C1_A	chr21:	FALSE	TRUE	TRUE	0.019	0.204	0.181	1	0	cassette	0.181
384	ENSG000002	ENSG000002	ENSG000002	chr21	+	ENSG000002	ENSG000002	TRUE	cassette^474|alt3^206|a		C1_A	chr21:	FALSE	FALSE	FALSE	0.012	0.141	0.107	0.911	0.044	cassette	0.107
385	ENSG000002	ENSG000002	ENSG000002	chr21	+	ENSG000002	ENSG000002	TRUE	cassette^474|alt3^206|a		C1_A	chr21:	FALSE	FALSE	TRUE	0.004	0.139	0.109	1	0	cassette	0.109
386	ENSG000002	ENSG000002	ENSG000002	chr21	+	ENSG000002	ENSG000002	TRUE	cassette^474|alt3^206|a		C1_A	chr21:	FALSE	FALSE	TRUE	0.011	0.214	0.184	0.985	0.004	cassette	0.184
387	ENSG000002	ENSG000002	ENSG000002	chr21	+	ENSG000002	ENSG000002	TRUE	cassette^474|alt3^206|a		C1_A	chr21:	FALSE	FALSE	TRUE	0.001	0.146	0.127	0.974	0.011	cassette	0.127
388	ENSG000002	ENSG000002	ENSG000002	chr21	+	ENSG000002	ENSG000002	TRUE	cassette^474	TRUE	Distal	chr21:84004	FALSE	FALSE	TRUE	0.003	0.212	0.183	1	0	AFE/ALE	0.183
389	ENSG000002	ENSG000002	ENSG000002	chr21	+	ENSG000002	ENSG000002	TRUE	cassette^474	TRUE	C1_A	chr21:84005	FALSE	FALSE	TRUE	0.001	0.249	0.232	1	0	cassette	0.232
390	ENSG000002	ENSG000002	ENSG000002	chr21	+	ENSG000002	ENSG000002	TRUE	cassette^474|alt3^206|a		C1_A	chr21:	FALSE	FALSE	TRUE	0.035	0.271	0.233	0.992	0.001	cassette	0.233
391	ENSG000002	ENSG000002	ENSG000002	chr21	+	ENSG000002	ENSG000002	TRUE	cassette^474	TRUE	C1_A	chr21:84011	FALSE	FALSE	TRUE	0.003	0.198	0.173	1	0	cassette	0.173
392	ENSG000002	ENSG000002	ENSG000002	chr21	+	ENSG000002	ENSG000002	TRUE	cassette^474|alt3^206|a		C1_A	chr21:	FALSE	FALSE	TRUE	0.005	0.128	0.104	0.953	0.024	cassette	0.104
393	ENSG000002	ENSG000002	ENSG000002	chr21	+	ENSG000002	ENSG000002	TRUE	cassette^474|alt3^206|a		C1_A	chr21:	FALSE	FALSE	TRUE	0.022	0.276	0.246	1	0	cassette	0.246
394	ENSG000002	ENSG000002	ENSG000002	chr21	+	ENSG000002	ENSG000002	TRUE	cassette^474|alt3^206|a		C1_A	chr21:	FALSE	FALSE	FALSE	0.036	0.253	0.194	0.907	0.009	cassette	0.194
395	ENSG000002	ENSG000002	ENSG000002	chr21	+	ENSG000002	ENSG000002	TRUE	cassette^474|alt3^206|a		C1_A	chr21:	FALSE	FALSE	FALSE	0.016	0.13	0.104	0.922	0.04	cassette	0.104
396	ENSG000002	ENSG000002	ENSG000002	chr21	+	ENSG000002	ENSG000002	TRUE	cassette^474|alt3^206|a		C1_A	chr21:	FALSE	FALSE	TRUE	0	0.156	0.131	0.993	0.003	cassette	0.131
397	ENSG000002	ENSG000002	ENSG000002	chr21	+	ENSG000002	ENSG000002	TRUE	cassette^474|alt3^206|a		C1_A	chr21:	FALSE	FALSE	TRUE	0.001	0.139	0.117	0.986	0.006	cassette	0.117
398	ENSG000002	ENSG000002	ENSG000002	chr21	+	ENSG000002	ENSG000002	TRUE	cassette^474|alt3^206|a		C1_A	chr21:	FALSE	FALSE	TRUE	0.002	0.206	0.189	0.988	0.003	cassette	0.189
399	ENSG000002	ENSG000002	ENSG000002	chr21	+	ENSG000002	ENSG000002	TRUE	cassette^474|alt3^206|a		C1_A	chr21:	FALSE	FALSE	TRUE	0.002	0.222	0.209	0.994	0.001	cassette	0.209
400	ENSG000002	ENSG000002	ENSG000002	chr21	+	ENSG000002	ENSG000002	TRUE	cassette^474|alt3^206|a		C1_A	chr21:	FALSE	FALSE	TRUE	0	0.234	0.215	1	0	cassette	0.215
401	ENSG000002	ENSG000002	ENSG000002	chr21	+	ENSG000002	ENSG000002	TRUE	cassette^474|alt3^206|a		C1_A	chr21:	FALSE	FALSE	TRUE	0	0.295	0.27	1	0	cassette	0.27
402	ENSG000002	ENSG000002	ENSG000002	chr21	+	ENSG000002	ENSG000002	TRUE	cassette^474|alt3^206|a		C1_A	chr21:	FALSE	FALSE	FALSE	0.029	0.156	0.135	0.939	0.015	cassette	0.135
403	ENSG000002	ENSG000002	ENSG000002	chr21	+	ENSG000002	ENSG000002	TRUE	cassette^474|alt3^206|a		C1_A	chr21:	FALSE	FALSE	TRUE	0.026	0.212	0.193	0.982	0.004	cassette	0.193
404	ENSG000002	ENSG000002	ENSG000002	chr21	+	ENSG000002	ENSG000002	TRUE	cassette^474|alt3^206|a		C1_A	chr21:	FALSE	FALSE	TRUE	0.045	0.236	0.211	0.992	0.002	cassette	0.211
405	ENSG000002	ENSG000002	ENSG000002	chr21	+	ENSG000002	ENSG000002	TRUE	cassette^474|alt3^206|a		C1_A	chr21:	FALSE	FALSE	TRUE	0.048	0.224	0.18	0.982	0.005	cassette	0.18
406	ENSG000002	ENSG000002	ENSG000002	chr21	+	ENSG000002	ENSG000002	TRUE	cassette^474|alt3^206|a		C1_A	chr21:	FALSE	FALSE	TRUE	0	0.15	0.133	0.976	0.01	cassette	0.133
407	ENSG000002	ENSG000002	ENSG000002	chr21	+	ENSG000002	ENSG000002	TRUE	cassette^474|alt3^206|a		C1_A	chr21:	FALSE	FALSE	TRUE	0	0.145	0.134	0.952	0.018	cassette	0.134
408	ENSG000002	ENSG000002	ENSG000002	chr21	+	ENSG000002	ENSG000002	TRUE	cassette^474|alt3^206|a		C1_A	chr21:	FALSE	FALSE	TRUE	0	0.422	0.401	1	0	cassette	0.401
409	ENSG000002	ENSG000002	ENSG000002	chr21	+	ENSG000002	ENSG000002	TRUE	cassette^474|alt3^206|a		C1_A	chr21:	FALSE	FALSE	TRUE	0	0.395	0.383	1	0	cassette	0.383
410	ENSG000002	ENSG000002	ENSG000002	chr21	+	ENSG000002	ENSG000002	TRUE	cassette^474|alt3^206|a		C1_A	chr21:	FALSE	FALSE	TRUE	0	0.232	0.211	1	0	cassette	0.211
411	ENSG000002	ENSG000002	ENSG000002	chr21	+	ENSG000002	ENSG000002	TRUE	cassette^474|alt3^206|a		C1_A	chr21:	FALSE	FALSE	TRUE	0	0.261	0.231	0.997	0	cassette	0.231
412	ENSG000002	ENSG000002	ENSG000002	chr21	+	ENSG000002	ENSG000002	TRUE	cassette^474|alt3^206|a		C1_A	chr21:	FALSE	FALSE	TRUE	0.001	0.193	0.168	0.981	0.005	cassette	0.168
413	ENSG000002	ENSG000002	ENSG000002	chr21	+	ENSG000002	ENSG000002	TRUE	cassette^474|alt3^206|a		C1_A	chr21:	FALSE	FALSE	TRUE	0.011	0.286	0.264	1	0	cassette	0.264
414	ENSG000002	ENSG000002	ENSG000002	chr21	+	ENSG000002	ENSG000002	TRUE	cassette^474|alt3^206|a		C1_A	chr21:	FALSE	FALSE	FALSE	0.024	0.137	0.111	0.927	0.03	cassette	0.111
415	ENSG000002	ENSG000002	ENSG000002	chr21	+	ENSG000002	ENSG000002	TRUE	cassette^474|alt3^206|a		C1_A	chr21:	FALSE	FALSE	TRUE	0.007	0.228	0.191	0.955	0.006	cassette	0.191
416	ENSG000002	ENSG000002	ENSG000002	chr21	+	ENSG000002	ENSG000002	TRUE	cassette^474|alt3^206|a		C1_A	chr21:	FALSE	FALSE	TRUE	0.007	0.385	0.33	0.995	0	cassette	0.33
417	ENSG000002	ENSG000002	ENSG000002	chr21	+	ENSG000002	ENSG000002	TRUE	cassette^474|alt3^206|a		C1_A	chr21:	FALSE	FALSE	TRUE	0.001	0.588	0.576	1	0	cassette	0.576
418	ENSG000002	ENSG000002	ENSG000002	chr21	+	ENSG000002	ENSG000002	TRUE	cassette^474|alt3^206|a		C1_A	chr21:	FALSE	FALSE	TRUE	0.001	0.175	0.147	0.991	0.003	cassette	0.147
419	ENSG000002	ENSG000002	ENSG000002	chr21	+	ENSG000002	ENSG000002	TRUE	cassette^474|alt3^206|a		C1_A	chr21:	FALSE	FALSE	TRUE	0.024	0.274	0.248	0.977	0.002	cassette	0.248
420	ENSG000002	ENSG000002	ENSG000002	chr21	+	ENSG000002	ENSG000002	TRUE	cassette^474|alt3^206|a		C1_A	chr21:	FALSE	FALSE	FALSE	0.003	0.208	0.173	0.945	0.011	cassette	0.173
421	ENSG000002	ENSG000002	ENSG000002	chr21	+	ENSG000002	ENSG000002	TRUE	cassette^474|alt3^206|a		C1_A	chr21:	FALSE	FALSE	TRUE	0.003	0.293	0.268	1	0	cassette	0.268
422	ENSG000002	ENSG000002	ENSG000002	chr21	+	ENSG000002	ENSG000002	TRUE	cassette^474|alt3^206|a		C1_A	chr21:	FALSE	FALSE	TRUE	0	0.141	0.116	0.954	0.021	cassette	0.116
423	ENSG000002	ENSG000002	ENSG000002	chr21	+	ENSG000002	ENSG000002	TRUE	cassette^474|alt3^206|a		C1_A	chr21:	FALSE	FALSE	TRUE	0.006	0.425	0.394	1	0	cassette	0.394
424	ENSG000002	ENSG000002	ENSG000002	chr21	+	ENSG000002	ENSG000002	TRUE	cassette^474|alt3^206|a		C1_A	chr21:	FALSE	FALSE	TRUE	0.007	0.251	0.224	0.998	0	cassette	0.224
425	ENSG000002	ENSG000002	ENSG000002	chr21	+	ENSG000002	ENSG000002	TRUE	cassette^474|alt3^206|a		C1_A	chr21:	FALSE	FALSE	TRUE	0.001	0.338	0.3	0.996	0	cassette	0.3
426	ENSG000002	ENSG000002	ENSG000002	chr21	+	ENSG000002	ENSG000002	TRUE	cassette^474|alt3^206|a		C1_A	chr21:	FALSE	FALSE	TRUE	0.004	0.251	0.233	0.999	0	cassette	0.233
427	ENSG000002	ENSG000002	ENSG000002	chr21	+	ENSG000002	ENSG000002	TRUE	cassette^474	TRUE	Distal	chr21:84404	FALSE	FALSE	TRUE	0.001	0.338	0.3	0.996	0	AFE/ALE	0.3
428	ENSG000002	ENSG000002	ENSG000002	chr21	+	ENSG000002	ENSG000002	TRUE	cassette^474|alt3^206|a		C1_A	chr21:	FALSE	FALSE	TRUE	0.015	0.438	0.416	1	0	cassette	0.416
429	ENSG000002	ENSG000002	ENSG000002	chr21	+	ENSG000002	ENSG000002	TRUE	cassette^474|alt3^206|a		C1_A	chr21:	FALSE	FALSE	TRUE	0.008	0.993	0.973	1	0	cassette	0.973
430	ENSG000002	ENSG000002	ENSG000002	chr21	+	ENSG000002	ENSG000002	TRUE	cassette^474|alt3^206|a		C1_A	chr21:	FALSE	FALSE	TRUE	0.005	0.207	0.183	0.988	0.003	cassette	0.183
431	ENSG000002	ENSG000002	ENSG000002	chr21	+	ENSG000002	ENSG000002	TRUE	cassette^474	TRUE	Distal	chr21:84448	FALSE	FALSE	TRUE	0.001	0.176	0.149	0.958	0.009	AFE/ALE	0.149
432	ENSG000002	ENSG000002	ENSG000002	chr21	+	ENSG000002	ENSG000002	TRUE	cassette^474|alt3^206|a		C1_A	chr21:	FALSE	FALSE	TRUE	0.003	0.176	0.149	0.966	0.012	cassette	0.149
433	ENSG000002	ENSG000002	ENSG000002	chr21	+	ENSG000002	ENSG000002	TRUE	cassette^474	TRUE	Proximal	chr21:83819	FALSE	TRUE	TRUE	0.001	0.824	0.815	1	0	AFE/ALE	0.815
434	ENSG000002	ENSG000002	ENSG000002	chr21	+	ENSG000002	ENSG000002	TRUE	cassette^474	TRUE	C1_A2	chr21:83825	FALSE	FALSE	TRUE	0	0.313	0.296	1	0	cassette	0.296
435	ENSG000002	ENSG000002	ENSG000002	chr21	+	ENSG000002	ENSG000002	TRUE	cassette^474	TRUE	Distal	chr21:83889	FALSE	FALSE	TRUE	0	0.586	0.579	1	0	AFE/ALE	0.579
436	ENSG000002	ENSG000002	ENSG000002	chr21	+	ENSG000002	ENSG000002	TRUE	cassette^474	TRUE	Distal	chr21:83903	FALSE	FALSE	FALSE	0.035	0.214	0.168	0.937	0.011	AFE/ALE	0.168
437	ENSG000002	ENSG000002	ENSG000002	chr21	+	ENSG000002	ENSG000002	TRUE	cassette^474	TRUE	Proximal	chr21:83908	FALSE	TRUE	TRUE	0.001	0.259	0.234	1	0	AFE/ALE	0.234
438	ENSG000002	ENSG000002	ENSG000002	chr21	+	ENSG000002	ENSG000002	TRUE	cassette^474	TRUE	Proximal	chr21:83908	FALSE	FALSE	TRUE	0.007	0.214	0.192	0.991	0.002	AFE/ALE	0.192
439	ENSG000002	ENSG000002	ENSG000002	chr21	+	ENSG000002	ENSG000002	TRUE	cassette^474	TRUE	Distal	chr21:83909	FALSE	FALSE	TRUE	0.006	0.21	0.176	0.974	0.007	AFE/ALE	0.176
440	ENSG000002	ENSG000002	ENSG000002	chr21	+	ENSG000002	ENSG000002	TRUE	cassette^474	TRUE	Distal	chr21:83914	FALSE	TRUE	TRUE	0.002	0.364	0.339	1	0	AFE/ALE	0.339
441	ENSG000002	ENSG000002	ENSG000002	chr21	+	ENSG000002	ENSG000002	TRUE	cassette^474	TRUE	C1_A2	chr21:83919	FALSE	FALSE	TRUE	0.006	0.484	0.468	1	0	cassette	0.468
442	ENSG000002	ENSG000002	ENSG000002	chr21	+	ENSG000002	ENSG000002	TRUE	cassette^474	TRUE	Proximal	chr21:84208	FALSE	TRUE	TRUE	0.005	0.72	0.69	1	0	AFE/ALE	0.69
443	ENSG000002	ENSG000002	ENSG000002	chr21	+	ENSG000002	ENSG000002	TRUE	cassette^474	TRUE	C1_C2	chr21:84299	FALSE	FALSE	TRUE	0.001	0.242	-0.21	0.991	0.002	skipping	0.21
444	ENSG000002	ENSG000002	ENSG000002	chr21	+	ENSG000002	ENSG000002	TRUE	cassette^474	TRUE	Proximal	chr21:83852	FALSE	FALSE	TRUE	0.007	0.264	0.239	1	0	AFE/ALE	0.239
445	ENSG000002	ENSG000002	ENSG000002	chr21	+	ENSG000002	ENSG000002	TRUE	cassette^474	TRUE	Proximal	chr21:83902	FALSE	FALSE	TRUE	0.003	0.186	0.16	0.995	0.002	AFE/ALE	0.16
446	ENSG000002	ENSG000002	ENSG000002	chr21	+	ENSG000002	ENSG000002	TRUE	cassette^474	TRUE	E2_E1_J1	chr21:83810	FALSE	FALSE	TRUE	0.007	0.429	0.39	1	0	cassette	0.39
447	ENSG000002	ENSG000002	ENSG000002	chr21	+	ENSG000002	ENSG000002	TRUE	cassette^474|alt3^206|a		A_C2	chr21:	FALSE	FALSE	TRUE	0.001	0.242	0.22	0.989	0.002	cassette	0.22
448	ENSG000002	ENSG000002	ENSG000002	chr21	+	ENSG000002	ENSG000002	TRUE	cassette^474	TRUE	Proximal	chr21:83896	FALSE	FALSE	TRUE	0.026	0.589	0.546	1	0	AFE/ALE	0.546
449	ENSG000002	ENSG000002	ENSG000002	chr21	+	ENSG000002	ENSG000002	TRUE	cassette^474	TRUE	Proximal	chr21:83900	FALSE	FALSE	FALSE	0.001	0.176	0.142	0.926	0.025	AFE/ALE	0.142
450	ENSG000002	ENSG000002	ENSG000002	chr21	+	ENSG000002	ENSG000002	TRUE	cassette^474	TRUE	Proximal	chr21:83819	FALSE	FALSE	TRUE	0	0.158	0.135	1	0	AFE/ALE	0.135
451	ENSG000002	ENSG000002	ENSG000002	chr21	+	ENSG000002	ENSG000002	TRUE	cassette^474	TRUE	E2_E1_J1	chr21:83843	FALSE	FALSE	FALSE	0.043	0.36	0.279	0.933	0.003	cassette	0.279
452	ENSG000002	ENSG000002	ENSG000002	chr21	+	ENSG000002	ENSG000002	TRUE	cassette^474|alt3^206|a		A_C2	chr21:	FALSE	FALSE	TRUE	0.001	0.204	0.187	0.987	0.003	cassette	0.187
453	ENSG000002	ENSG000002	ENSG000002	chr21	+	ENSG000002	ENSG000002	TRUE	cassette^474	TRUE	Proximal	chr21:83896	FALSE	FALSE	TRUE	0.031	0.209	0.176	0.994	0.002	AFE/ALE	0.176
454	ENSG000002	ENSG000002	ENSG000002	chr21	+	ENSG000002	ENSG000002	TRUE	cassette^474	TRUE	Proximal	chr21:83881	FALSE	FALSE	TRUE	0	0.209	0.187	0.977	0.005	AFE/ALE	0.187
455	ENSG000002	ENSG000002	ENSG000002	chr21	+	ENSG000002	ENSG000002	TRUE	cassette^474	TRUE	Proximal	chr21:83823	FALSE	FALSE	FALSE	0.005	0.156	0.142	0.902	0.026	AFE/ALE	0.142

456	ENSG000002	ENSG000002	ENSG000002	chr21	+	ENSG000002	ENSG000002	TRUE	cassette^47	TRUE	Distal	chr21:83899	FALSE	FALSE	TRUE	0.001	0.217	0.197	0.977	0.005	AFE/ALE	0.197
457	ENSG000002	ENSG000002	ENSG000002	chr21	+	ENSG000002	ENSG000002	TRUE	cassette^474|alt3^206|a		A_C2	chr21:	FALSE	FALSE	FALSE	0	0.146	0.144	0.907	0.025	cassette	0.144
458	ENSG000002	ENSG000002	ENSG000002	chr21	+	ENSG000002	ENSG000002	TRUE	cassette^47	TRUE	Distal	chr21:83893	FALSE	FALSE	TRUE	0	0.446	0.439	1	0	AFE/ALE	0.439
459	ENSG000002	ENSG000002	ENSG000002	chr21	+	ENSG000002	ENSG000002	TRUE	cassette^47	TRUE	E2_E1_J2	chr21:83958	FALSE	FALSE	TRUE	0.045	0.176	0.136	0.972	0.011	cassette	0.136
460	ENSG000002	ENSG000002	ENSG000002	chr21	+	ENSG000002	ENSG000002	TRUE	cassette^474|alt3^206|a		A_C2	chr21:	FALSE	FALSE	TRUE	0	0.145	0.119	0.955	0.021	cassette	0.119
461	ENSG000002	ENSG000002	ENSG000002	chr21	+	ENSG000002	ENSG000002	TRUE	cassette^474|alt3^206|a		A_C2	chr21:	FALSE	FALSE	FALSE	0.005	0.154	0.12	0.925	0.03	cassette	0.12
462	ENSG000002	ENSG000002	ENSG000002	chr21	+	ENSG000002	ENSG000002	TRUE	cassette^474|alt3^206|a		A_C2	chr21:	FALSE	FALSE	TRUE	0.001	0.292	0.261	1	0	cassette	0.261
463	ENSG000002	ENSG000002	ENSG000002	chr21	+	ENSG000002	ENSG000002	TRUE	cassette^474|alt3^206|a		A_C2	chr21:	FALSE	FALSE	TRUE	0.003	0.162	0.137	0.983	0.007	cassette	0.137
464	ENSG000002	ENSG000002	ENSG000002	chr21	+	ENSG000002	ENSG000002	TRUE	cassette^47	TRUE	Proximal	chr21:83925	FALSE	FALSE	TRUE	0.004	0.285	0.255	0.984	0.001	AFE/ALE	0.255
465	ENSG000002	ENSG000002	ENSG000002	chr21	+	ENSG000002	ENSG000002	TRUE	cassette^47	TRUE	A_C2	chr21:83951	FALSE	FALSE	FALSE	0.001	0.164	0.147	0.946	0.016	cassette	0.147
466	ENSG000002	ENSG000002	ENSG000002	chr21	+	ENSG000002	ENSG000002	TRUE	cassette^474|alt3^206|a		A_C2	chr21:	FALSE	FALSE	TRUE	0.017	0.284	0.249	0.982	0.002	cassette	0.249
467	ENSG000002	ENSG000002	ENSG000002	chr21	+	ENSG000002	ENSG000002	TRUE	cassette^47	TRUE	Proximal	chr21:83886	FALSE	FALSE	TRUE	0.005	0.397	0.38	1	0	AFE/ALE	0.38
468	ENSG000002	ENSG000002	ENSG000002	chr21	+	ENSG000002	ENSG000002	TRUE	cassette^47	TRUE	Distal	chr21:83825	FALSE	FALSE	TRUE	0.001	0.498	0.503	1	0	AFE/ALE	0.503
469	ENSG000002	ENSG000002	ENSG000002	chr21	+	ENSG000002	ENSG000002	TRUE	cassette^474|alt3^206|a		A_C2	chr21:	FALSE	FALSE	FALSE	0	0.122	0.099	0.949	0.027	cassette	0.099
470	ENSG000002	ENSG000002	ENSG000002	chr21	+	ENSG000002	ENSG000002	TRUE	cassette^474|alt3^206|a		A_C2	chr21:	FALSE	FALSE	TRUE	0.046	0.255	0.208	1	0	cassette	0.208
471	ENSG000002	ENSG000002	ENSG000002	chr21	+	ENSG000002	ENSG000002	TRUE	cassette^474|alt3^206|a		A_C2	chr21:	FALSE	FALSE	TRUE	0.011	0.724	0.706	1	0	cassette	0.706
472	ENSG000002	ENSG000002	ENSG000002	chr21	+	ENSG000002	ENSG000002	TRUE	cassette^47	TRUE	C2_A	chr21:83988	FALSE	FALSE	TRUE	0.042	0.232	0.195	1	0	cassette	0.195
473	ENSG000002	ENSG000002	ENSG000002	chr21	+	ENSG000002	ENSG000002	TRUE	cassette^474|alt3^206|a		A_C2	chr21:	FALSE	FALSE	TRUE	0.034	0.27	0.229	0.999	0	cassette	0.229
474	ENSG000002	ENSG000002	ENSG000002	chr21	+	ENSG000002	ENSG000002	TRUE	cassette^474|alt3^206|a		A_C2	chr21:	FALSE	FALSE	TRUE	0.001	0.383	0.357	1	0	cassette	0.357
475	ENSG000002	ENSG000002	ENSG000002	chr21	+	ENSG000002	ENSG000002	TRUE	cassette^47	TRUE	Distal	chr21:83824	FALSE	FALSE	TRUE	0	0.128	0.105	0.954	0.023	AFE/ALE	0.105
476	ENSG000002	ENSG000002	ENSG000002	chr21	+	ENSG000002	ENSG000002	TRUE	cassette^47	TRUE	Distal	chr21:83889	FALSE	FALSE	TRUE	0	0.386	0.358	1	0	AFE/ALE	0.358
477	ENSG000002	ENSG000002	ENSG000002	chr21	+	ENSG000002	ENSG000002	TRUE	cassette^474|alt3^206|a		A_C2	chr21:	FALSE	FALSE	TRUE	0.02	0.241	0.207	0.999	0	cassette	0.207
478	ENSG000002	ENSG000002	ENSG000002	chr21	+	ENSG000002	ENSG000002	TRUE	cassette^47	TRUE	C2_A1	chr21:84011	FALSE	FALSE	TRUE	0.011	0.272	0.25	1	0	cassette	0.25
479	ENSG000002	ENSG000002	ENSG000002	chr21	+	ENSG000002	ENSG000002	TRUE	cassette^47	TRUE	Distal	chr21:83939	FALSE	FALSE	TRUE	0.019	0.498	0.492	1	0	AFE/ALE	0.492
480	ENSG000002	ENSG000002	ENSG000002	chr21	+	ENSG000002	ENSG000002	TRUE	cassette^47	TRUE	Proximal	chr21:84299	FALSE	FALSE	TRUE	0.001	0.409	0.37	0.998	0	AFE/ALE	0.37
481	ENSG000002	ENSG000002	ENSG000002	chr21	+	ENSG000002	ENSG000002	TRUE	cassette^474|alt3^206|a		A_C2	chr21:	FALSE	FALSE	TRUE	0.001	0.336	0.309	0.999	0	cassette	0.309
482	ENSG000002	ENSG000002	ENSG000002	chr21	+	ENSG000002	ENSG000002	TRUE	cassette^47	TRUE	A_C2	chr21:84078	FALSE	FALSE	FALSE	0.011	0.118	0.099	0.916	0.045	cassette	0.099
483	ENSG000002	ENSG000002	ENSG000002	chr21	+	ENSG000002	ENSG000002	TRUE	cassette^47	TRUE	A_C2	chr21:84088	FALSE	FALSE	FALSE	0.04	0.165	0.128	0.92	0.03	cassette	0.128
484	ENSG000002	ENSG000002	ENSG000002	chr21	+	ENSG000002	ENSG000002	TRUE	cassette^474|alt3^206|a		A_C2	chr21:	FALSE	FALSE	TRUE	0.004	0.296	0.268	1	0	cassette	0.268
485	ENSG000002	ENSG000002	ENSG000002	chr21	+	ENSG000002	ENSG000002	TRUE	cassette^47	TRUE	A_C2	chr21:84107	FALSE	FALSE	TRUE	0.009	0.466	0.456	1	0	cassette	0.456
486	ENSG000002	ENSG000002	ENSG000002	chr21	+	ENSG000002	ENSG000002	TRUE	cassette^474|alt3^206|a		A_C2	chr21:	FALSE	FALSE	TRUE	0.012	0.254	0.225	1	0	cassette	0.225
487	ENSG000002	ENSG000002	ENSG000002	chr21	+	ENSG000002	ENSG000002	TRUE	cassette^474|alt3^206|a		A_C2	chr21:	FALSE	FALSE	FALSE	0.026	0.148	0.126	0.913	0.022	cassette	0.126
488	ENSG000002	ENSG000002	ENSG000002	chr21	+	ENSG000002	ENSG000002	TRUE	cassette^474|alt3^206|a		A_C2	chr21:	FALSE	FALSE	TRUE	0.003	0.184	0.156	0.957	0.013	cassette	0.156
489	ENSG000002	ENSG000002	ENSG000002	chr21	+	ENSG000002	ENSG000002	TRUE	cassette^474|alt3^206|a		A_C2	chr21:	FALSE	FALSE	TRUE	0.006	0.3	0.283	1	0	cassette	0.283
490	ENSG000002	ENSG000002	ENSG000002	chr21	+	ENSG000002	ENSG000002	TRUE	cassette^474|alt3^206|a		A_C2	chr21:	FALSE	FALSE	FALSE	0	0.146	0.125	0.93	0.029	cassette	0.125
491	ENSG000002	ENSG000002	ENSG000002	chr21	+	ENSG000002	ENSG000002	TRUE	cassette^474|alt3^206|a		A_C2	chr21:	FALSE	FALSE	TRUE	0.01	0.126	0.103	0.985	0.008	cassette	0.103
492	ENSG000002	ENSG000002	ENSG000002	chr21	+	ENSG000002	ENSG000002	TRUE	cassette^47	TRUE	Distal	chr21:83987	FALSE	FALSE	FALSE	0.001	0.171	0.143	0.929	0.022	AFE/ALE	0.143
493	ENSG000002	ENSG000002	ENSG000002	chr21	+	ENSG000002	ENSG000002	TRUE	cassette^474|alt3^206|a		A_C2	chr21:	FALSE	FALSE	FALSE	0.001	0.171	0.143	0.929	0.022	cassette	0.143
494	ENSG000002	ENSG000002	ENSG000002	chr21	+	ENSG000002	ENSG000002	TRUE	cassette^474|alt3^206|a		A_C2	chr21:	FALSE	FALSE	TRUE	0	0.169	0.142	0.985	0.006	cassette	0.142
495	ENSG000002	ENSG000002	ENSG000002	chr21	+	ENSG000002	ENSG000002	TRUE	cassette^474|alt3^206|a		A_C2	chr21:	FALSE	FALSE	FALSE	0.034	0.229	0.185	0.944	0.008	cassette	0.185
496	ENSG000002	ENSG000002	ENSG000002	chr21	+	ENSG000002	ENSG000002	TRUE	cassette^474|alt3^206|a		A_C2	chr21:	FALSE	FALSE	TRUE	0	0.182	0.152	0.99	0.004	cassette	0.152
497	ENSG000002	ENSG000002	ENSG000002	chr21	+	ENSG000002	ENSG000002	TRUE	cassette^47	TRUE	Proximal	chr21:84386	FALSE	FALSE	FALSE	0.023	0.181	0.153	0.936	0.016	AFE/ALE	0.153
498	ENSG000002	ENSG000002	ENSG000002	chr21	+	ENSG000002	ENSG000002	TRUE	cassette^474|alt3^206|a		A_C2	chr21:	FALSE	FALSE	FALSE	0.023	0.181	0.153	0.936	0.016	cassette	0.153
499	ENSG000002	ENSG000002	ENSG000002	chr21	+	ENSG000002	ENSG000002	TRUE	cassette^474|alt3^206|a		A_C2	chr21:	FALSE	FALSE	TRUE	0.013	0.283	0.263	1	0	cassette	0.263
500	ENSG000002	ENSG000002	ENSG000002	chr21	+	ENSG000002	ENSG000002	TRUE	cassette^474|alt3^206|a		A_C2	chr21:	FALSE	FALSE	TRUE	0	0.244	0.22	1	0	cassette	0.22
501	ENSG000002	ENSG000002	ENSG000002	chr21	+	ENSG000002	ENSG000002	TRUE	cassette^47	TRUE	C2_A1	chr21:84448	FALSE	FALSE	TRUE	0.001	0.207	0.174	0.971	0.004	cassette	0.174
502	ENSG000002	ENSG000002	ENSG000002	chr1	-	ENSG000002	ENSG000002	TRUE	putative_afe	TRUE	Distal	chr1:167406	FALSE	TRUE	TRUE	0.001	0.227	0.21	1	0	AFE/ALE	0.21
503	ENSG000002	ENSG000002	ENSG000002	chr14	-	ENSG000002	ENSG000002	TRUE	alt3^4|alt5^	TRUE	E1_E2_J2	chr14:49862	FALSE	FALSE	FALSE	0	0.121	0.098	0.918	0.044	cassette	0.098
504	ENSG000002	ENSG000002	ENSG000002	chr19	+	ENSG000002	ENSG000002	TRUE	alt3^1|putat	TRUE	Proximal	chr19:35558	FALSE	FALSE	TRUE	0.001	0.165	0.137	0.959	0.016	AFE/ALE	0.137
505	ENSG000002	ENSG000002	ENSG000002	chr19	+	ENSG000002	ENSG000002	TRUE	alt3^1|putat	TRUE	Proximal	chr19:35559	FALSE	FALSE	TRUE	0.001	0.162	0.133	0.966	0.014	AFE/ALE	0.133
506	ENSG000002	ENSG000002	ENSG000002	chr19	+	ENSG000002	ENSG000002	TRUE	alt3^1|putat	TRUE	Proximal	chr19:35563	FALSE	FALSE	TRUE	0.001	0.229	0.198	1	0	AFE/ALE	0.198
507	ENSG000002	ENSG000002	ENSG000002	chr19	+	ENSG000002	ENSG000002	TRUE	cassette^2|	TRUE	C1_A	chr19:58583	FALSE	FALSE	TRUE	0.011	0.25	0.223	1	0	cassette	0.223

Table S2: Identified cryptic exons in hnRNPA1 KD neurons.

	module_id	gene_id	gene_name	seqid	strand	lsv_id	event_id	complex	module_even	denovo	junction_nan	junctions_co	event_non_cl	event_chang	junction_cha	refPSI	KDSoma_me	deltaPSI	prob	padjust	event	abs(deltaPSI)
1	ENSG000001	ENSG000001	BRD8	chr5	-		ENSG000001	FALSE	putative_alt5	FALSE	Proximal	chr5:138157	FALSE	FALSE	TRUE	0.017	0.147	0.119	0.961	0.016	AFE/ALE	0.119
2	ENSG000000	ENSG000000	ZNF839	chr14	+	ENSG000000	ENSG000000	TRUE	ir^2|afe^2	FALSE	Distal	chr14:10231	FALSE	FALSE	TRUE	0.029	0.267	0.215	0.971	0.003	AFE/ALE	0.215
3	ENSG000000	ENSG000000	ANK1	chr8	-	ENSG000000	ENSG000000	FALSE	putative_ale	TRUE	Proximal	chr8:418187	FALSE	TRUE	TRUE	0.005	0.369	0.346	1	0	AFE/ALE	0.346
4	ENSG000000	ENSG000000	CROCC	chr1	+	ENSG000000	ENSG000000	TRUE	ir^1|ale^13|afe^5|putati		C1_A	chr1:	FALSE	TRUE	TRUE	0	0.517	0.505	1	0	cassette	0.505
5	ENSG000000	ENSG000000	CROCC	chr1	+	ENSG000000	ENSG000000	TRUE	ir^1|ale^13|afe^5|putati		A_C2	chr1:	FALSE	FALSE	TRUE	0.019	0.488	0.475	1	0	cassette	0.475
6	ENSG000000	ENSG000000	CROCC	chr1	+	ENSG000000	ENSG000000	TRUE	ir^1|ale^13|	TRUE	Distal	chr1:168961	FALSE	TRUE	TRUE	0.001	0.804	0.797	1	0	AFE/ALE	0.797
7	ENSG000000	ENSG000000	TNRC6A	chr16	+	ENSG000000	ENSG000000	TRUE	cassette^2|t	FALSE	A_C2	chr16:24758	FALSE	FALSE	FALSE	0.035	0.14	0.105	0.905	0.04	cassette	0.105
8	ENSG000001	ENSG000001	BBC3	chr19	-	ENSG000001	ENSG000001	FALSE	afe^1	TRUE	Distal	chr19:47226	FALSE	FALSE	FALSE	0.047	0.203	0.149	0.924	0.016	AFE/ALE	0.149
9	ENSG000001	ENSG000001	CBL	chr11	+	ENSG000001	ENSG000001	FALSE	cassette^1	TRUE	C2_C1	chr11:11920	FALSE	FALSE	FALSE	0.028	0.17	-0.132	0.943	0.018	skipping	0.132
10	ENSG000001	ENSG000001	ERI3	chr1	-	ENSG000001	ENSG000001	TRUE	cassette^3|t	FALSE	C1_A	chr1:443529	FALSE	FALSE	FALSE	0.032	0.138	0.104	0.926	0.033	cassette	0.104
11	ENSG000001	ENSG000001	GRIA3	chrX	+	ENSG000001	ENSG000001	FALSE	ale^1	FALSE	Proximal	chrX:123185	FALSE	FALSE	FALSE	0.016	0.163	0.126	0.928	0.024	AFE/ALE	0.126
12	ENSG000001	ENSG000001	RFX1	chr19	-	ENSG000001	ENSG000001	FALSE	cassette^1	TRUE	C1_A	chr19:13979	FALSE	FALSE	TRUE	0.041	0.261	0.215	0.996	0.001	cassette	0.215
13	ENSG000001	ENSG000001	DAGLA	chr11	+	ENSG000001	ENSG000001	FALSE	putative_ale	TRUE	Proximal	chr11:61726	FALSE	FALSE	FALSE	0.041	0.178	0.135	0.913	0.023	AFE/ALE	0.135
14	ENSG000001	ENSG000001	RHBDL3	chr17	+	ENSG000001	ENSG000001	TRUE	cassette^1|i	FALSE	C2_C1	chr17:32266	FALSE	FALSE	FALSE	0.045	0.229	-0.16	0.913	0.013	skipping	0.16
15	ENSG000001	ENSG000001	FAM171B	chr2	+	ENSG000001	ENSG000001	FALSE	putative_ale	TRUE	Proximal	chr2:186694	FALSE	FALSE	FALSE	0.026	0.121	0.094	0.901	0.048	AFE/ALE	0.094
16	ENSG000001	ENSG000001	RAPGEF6	chr5	-	ENSG000001	ENSG000001	TRUE	putative_alt3	FALSE	Proximal	chr5:131436	FALSE	FALSE	TRUE	0.008	0.28	0.255	1	0	AFE/ALE	0.255
17	ENSG000001	ENSG000001	SPON2	chr4	-	ENSG000001	ENSG000001	TRUE	afe^1|putati	TRUE	Distal	chr4:117230	FALSE	FALSE	TRUE	0.028	0.219	0.174	0.961	0.006	AFE/ALE	0.174
18	ENSG000001	ENSG000001	TEX264	chr3	+	ENSG000001	ENSG000001	TRUE	cassette^3|	FALSE	C1_C2	chr3:516745	FALSE	FALSE	TRUE	0.039	0.21	-0.164	0.967	0.008	skipping	0.164
19	ENSG000001	ENSG000001	PPP2R3B	chrX	-	ENSG000001	ENSG000001	TRUE	cassette^1|	FALSE	C2_A	chrX:361590	FALSE	FALSE	TRUE	0.031	0.179	0.147	0.974	0.008	cassette	0.147
20	ENSG000001	ENSG000001	SPNS1	chr16	+	ENSG000001	ENSG000001	TRUE	cassette^2|	TRUE	Proximal	chr16:28978	FALSE	TRUE	TRUE	0.05	0.383	0.329	1	0	AFE/ALE	0.329
21	ENSG000001	ENSG000001	ZNF417	chr19	-	ENSG000001	ENSG000001	TRUE	cassette^2|	FALSE	C1_A	chr19:57915	FALSE	FALSE	TRUE	0.012	0.192	0.156	0.988	0.003	cassette	0.156
22	ENSG000001	ENSG000001	PITPNA	chr17	-	ENSG000001	ENSG000001	FALSE	alt5^1	TRUE	Proximal	chr17:15342	FALSE	FALSE	TRUE	0.035	0.144	0.119	0.991	0.004	AFE/ALE	0.119
23	ENSG000001	ENSG000001	ZNF25	chr10	-	ENSG000001	ENSG000001	TRUE	putative_afe	TRUE	Proximal	chr10:37971	FALSE	FALSE	FALSE	0.042	0.208	0.15	0.913	0.017	AFE/ALE	0.15
24	ENSG000001	ENSG000001	GUSBP1	chr5	+	ENSG000001	ENSG000001	TRUE	cassette^1|i	FALSE	A_C2	chr5:214915	FALSE	FALSE	FALSE	0.017	0.156	0.121	0.916	0.031	cassette	0.121
25	ENSG000001	ENSG000001	WASH3P	chr15	+	ENSG000001	ENSG000001	TRUE	cassette^3|	FALSE	Proximal	chr15:10197	FALSE	FALSE	TRUE	0.022	0.194	0.168	1	0	AFE/ALE	0.168
26	ENSG000001	ENSG000001	ZNF136	chr19	+	ENSG000001	ENSG000001	FALSE	cassette^1	FALSE	C1_C2	chr19:12163	FALSE	FALSE	TRUE	0.033	0.19	-0.149	0.967	0.009	skipping	0.149
27	ENSG000001	ENSG000001	SGTB	chr5	-	ENSG000001	ENSG000001	FALSE	cassette^1	TRUE	C1_A	chr5:657047	FALSE	FALSE	TRUE	0.027	0.161	0.133	0.954	0.015	cassette	0.133
28	ENSG000002	ENSG000002	PCDHA2	chr5	+	ENSG000002	ENSG000002	TRUE	ale^2|putati	TRUE	Proximal	chr5:140795	FALSE	FALSE	TRUE	0.001	0.311	0.286	1	0	AFE/ALE	0.286
29	ENSG000002	ENSG000002	PCDHA2	chr5	+	ENSG000002	ENSG000002	TRUE	ale^2|putative_ale^8|orp		C1_A	chr5:	FALSE	FALSE	TRUE	0.019	0.163	0.13	0.969	0.011	cassette	0.13
30	ENSG000002	ENSG000002	PCDHA1	chr5	+	ENSG000002	ENSG000002	TRUE	ale^2|putative_ale^2|orp		C1_A	chr5:	FALSE	FALSE	TRUE	0.019	0.163	0.13	0.969	0.011	cassette	0.13
31	ENSG000002	ENSG000002	ANKRD28	chr3	-	ENSG000002	ENSG000002	TRUE	cassette^2|	TRUE	C1_A	chr3:157125	FALSE	FALSE	TRUE	0	0.212	0.179	1	0	cassette	0.179
32	ENSG000002	ENSG000002	ANKRD28	chr3	-	ENSG000002	ENSG000002	TRUE	cassette^2|	TRUE	C1_A	chr3:157125	FALSE	FALSE	TRUE	0	0.28	0.257	0.993	0.001	cassette	0.257
33	ENSG000002	ENSG000002	ANKRD28	chr3	-	ENSG000002	ENSG000002	TRUE	cassette^2|	FALSE	A_C2	chr3:156783	FALSE	FALSE	TRUE	0.04	0.339	0.313	0.981	0.001	cassette	0.313
34	ENSG000002	ENSG000002	ANKRD28	chr3	-	ENSG000002	ENSG000002	TRUE	cassette^2|alt3^38|alt5^		A_C2	chr3:	FALSE	FALSE	FALSE	0.001	0.188	0.15	0.921	0.021	cassette	0.15
35	ENSG000002	ENSG000002	ANKRD28	chr3	-	ENSG000002	ENSG000002	TRUE	cassette^2|	TRUE	E2_E1_J2	chr3:156957	FALSE	FALSE	TRUE	0	0.186	0.152	0.962	0.011	cassette	0.152
36	ENSG000002	ENSG000002	ANKRD28	chr3	-	ENSG000002	ENSG000002	TRUE	cassette^2|	TRUE	Distal	chr3:156962	FALSE	FALSE	TRUE	0	0.401	0.38	1	0	AFE/ALE	0.38
37	ENSG000002	ENSG000002	ANKRD28	chr3	-	ENSG000002	ENSG000002	TRUE	cassette^2|	TRUE	E2_E1_J2	chr3:157113	FALSE	FALSE	TRUE	0.013	0.167	0.135	0.951	0.015	cassette	0.135
38	ENSG000002	ENSG000002	ANKRD28	chr3	-	ENSG000002	ENSG000002	TRUE	cassette^2|alt3^38|alt5^		A_C2	chr3:	FALSE	FALSE	TRUE	0	0.158	0.133	0.979	0.008	cassette	0.133
39	ENSG000002	ENSG000002	ANKRD28	chr3	-	ENSG000002	ENSG000002	TRUE	cassette^2|	TRUE	E2_E1_J1	chr3:157161	FALSE	FALSE	TRUE	0.007	0.154	0.128	0.964	0.016	cassette	0.128
40	ENSG000002	ENSG000002	ANKRD28	chr3	-	ENSG000002	ENSG000002	TRUE	cassette^2|	TRUE	Distal	chr3:157069	FALSE	TRUE	TRUE	0.001	0.506	0.497	1	0	AFE/ALE	0.497
41	ENSG000002	ENSG000002	ANKRD28	chr3	-	ENSG000002	ENSG000002	TRUE	cassette^2|	TRUE	Distal	chr3:157120	FALSE	FALSE	TRUE	0.002	0.441	0.429	0.998	0	AFE/ALE	0.429
42	ENSG000002	ENSG000002	RPS29	chr14	-	ENSG000002	ENSG000002	TRUE	alt3^26|alt5	TRUE	E2_E1_J2	chr14:49575	FALSE	FALSE	TRUE	0.031	0.127	0.101	0.978	0.013	cassette	0.101
43	ENSG000002	ENSG000002	RPS29	chr14	-	ENSG000002	ENSG000002	TRUE	alt3^26|alt5	TRUE	E2_E1_J2	chr14:49586	FALSE	FALSE	FALSE	0	0.13	0.104	0.911	0.043	cassette	0.104
44	ENSG000002	ENSG000002	SNHG14	chr15	+	ENSG000002	ENSG000002	TRUE	cassette^13|alt3^3|alt5^		C1_A	chr15:	FALSE	FALSE	TRUE	0.007	0.529	0.522	1	0	cassette	0.522
45	ENSG000002	ENSG000002	SNHG14	chr15	+	ENSG000002	ENSG000002	TRUE	cassette^3|	TRUE	E1_E2_J2	chr15:25193	FALSE	FALSE	TRUE	0.002	0.484	0.466	1	0	cassette	0.466
46	ENSG000002	ENSG000002	SNHG14	chr15	+	ENSG000002	ENSG000002	TRUE	cassette^1|	FALSE	C1_A	chr15:25230	FALSE	TRUE	TRUE	0.021	0.713	0.693	1	0	cassette	0.693
47	ENSG000002	ENSG000002	SNHG14	chr15	+	ENSG000002	ENSG000002	TRUE	cassette^13	TRUE	E2_E1_J1	chr15:25056	FALSE	FALSE	TRUE	0.009	0.224	0.197	0.998	0	cassette	0.197
48	ENSG000002	ENSG000002	SNHG14	chr15	+	ENSG000002	ENSG000002	TRUE	cassette^13|alt3^3|alt5^		A_C2	chr15:	FALSE	FALSE	TRUE	0.006	0.215	0.201	0.968	0.004	cassette	0.201
49	ENSG000002	ENSG000002	SNHG14	chr15	+	ENSG000002	ENSG000002	TRUE	cassette^3|	TRUE	Distal	chr15:25193	FALSE	TRUE	TRUE	0.002	0.527	0.506	1	0	AFE/ALE	0.506
50	ENSG000002	ENSG000002	SNHG14	chr15	+	ENSG000002	ENSG000002	TRUE	cassette^1|	TRUE	Distal	chr15:25230	FALSE	TRUE	TRUE	0.021	0.715	0.705	1	0	AFE/ALE	0.705
51	ENSG000002	ENSG000002	PARG	chr10	-	ENSG000002	ENSG000002	FALSE	cassette^1	FALSE	C2_C1	chr10:49922	FALSE	FALSE	FALSE	0.033	0.2	-0.145	0.913	0.017	skipping	0.145
52	ENSG000002	ENSG000002	ENSG000002	chr21	+	ENSG000002	ENSG000002	TRUE	cassette^42|alt3^33|alt5		C1_A	chr21:	FALSE	TRUE	TRUE	0.003	0.273	0.251	1	0	cassette	0.251
53	ENSG000002	ENSG000002	ENSG000002	chr21	+	ENSG000002	ENSG000002	TRUE	cassette^42|alt3^33|alt5		C1_A	chr21:	FALSE	FALSE	FALSE	0.016	0.154	0.123	0.925	0.027	cassette	0.123
54	ENSG000002	ENSG000002	ENSG000002	chr21	+	ENSG000002	ENSG000002	TRUE	cassette^42|alt3^33|alt5		C1_A	chr21:	FALSE	FALSE	TRUE	0.001	0.226	0.196	0.996	0.001	cassette	0.196
55	ENSG000002	ENSG000002	ENSG000002	chr21	+	ENSG000002	ENSG000002	TRUE	cassette^42|alt3^33|alt5		C1_A	chr21:	FALSE	TRUE	TRUE	0.03	0.199	0.171	1	0	cassette	0.171
56	ENSG000002	ENSG000002	ENSG000002	chr21	+	ENSG000002	ENSG000002	TRUE	cassette^42|alt3^33|alt5		C1_A	chr21:	FALSE	FALSE	TRUE	0	0.203	0.184	0.998	0	cassette	0.184
57	ENSG000002	ENSG000002	ENSG000002	chr21	+	ENSG000002	ENSG000002	TRUE	cassette^42	TRUE	Distal	chr21:82151	FALSE	FALSE	TRUE	0	0.397	0.371	1	0	AFE/ALE	0.371
58	ENSG000002	ENSG000002	ENSG000002	chr21	+	ENSG000002	ENSG000002	TRUE	cassette^42	TRUE	E1_E2_J1	chr21:82171	FALSE	FALSE	TRUE	0.033	0.174	0.137	0.979	0.007	cassette	0.137
59	ENSG000002	ENSG000002	ENSG000002	chr21	+	ENSG000002	ENSG000002	TRUE	cassette^42	TRUE	C1_A	chr21:81990	FALSE	FALSE	TRUE	0.001	0.208	0.175	0.998	0.001	cassette	0.175
60	ENSG000002	ENSG000002	ENSG000002	chr21	+	ENSG000002	ENSG000002	TRUE	cassette^42	TRUE	Distal	chr21:82059	FALSE	FALSE	TRUE	0.001	0.335	0.294	1	0	AFE/ALE	0.294
61	ENSG000002	ENSG000002	ENSG000002	chr21	+	ENSG000002	ENSG000002	TRUE	cassette^42	TRUE	Distal	chr21:82005	FALSE	FALSE	TRUE	0.039	0.229	0.184	0.953	0.006	AFE/ALE	0.184
62	ENSG000002	ENSG000002	ENSG000002	chr21	+	ENSG000002	ENSG000002	TRUE	cassette^42	TRUE	C2_C1	chr21:82059	FALSE	FALSE	TRUE	0.002	0.241	-0.21	0.99	0.002	skipping	0.21
63	ENSG000002	ENSG000002	ENSG000002	chr21	+	ENSG000002	ENSG000002	TRUE	cassette^42	TRUE	Proximal	chr21:82109	FALSE	FALSE	TRUE	0.004	0.324	0.303	1	0	AFE/ALE	0.303
64	ENSG000002	ENSG000002	ENSG000002	chr21	+	ENSG000002	ENSG000002	TRUE	cassette^42|alt3^33|alt5		A_C2	chr21:	FALSE	FALSE	TRUE	0.012	0.351	0.317	1	0	cassette	0.317
65	ENSG000002	ENSG000002	ENSG000002	chr21	+	ENSG000002	ENSG000002	TRUE	cassette^42	TRUE	C2_C1	chr21:82072	FALSE	FALSE	FALSE	0.04	0.249	-0.17	0.927	0.011	skipping	0.17
66	ENSG000002	ENSG000002	ENSG000002	chr21	+	ENSG000002	ENSG000002	TRUE	cassette^42	TRUE	J12	chr21:82181	FALSE	FALSE	TRUE	0.027	0.303	0.276	0.999	0	cassette	0.276
67	ENSG000002	ENSG000002	ENSG000002	chr21	+	ENSG000002	ENSG000002	TRUE	cassette^42	TRUE	C2_A	chr21:82120	FALSE	FALSE	TRUE	0.002	0.188	0.17	0.971	0.007	cassette	0.17
68	ENSG000002	ENSG000002	ENSG000002	chr21	+	ENSG000002	ENSG000002	TRUE	cassette^42	TRUE	Proximal	chr21:82151	FALSE	TRUE	TRUE	0.002	0.685	0.683	1	0	AFE/ALE	0.683
69	ENSG000002	ENSG000002	ENSG000002	chr21	+	ENSG000002	ENSG000002	TRUE	cassette^42	TRUE	Distal	chr21:82063	FALSE	FALSE	TRUE	0.007	0.923	0.913	1	0	AFE/ALE	0.913
70	ENSG000002	ENSG000002	ENSG000002	chr21	+	ENSG000002	ENSG000002	TRUE	cassette^126|alt3^121|a		C1_A	chr21:	FALSE	FALSE	TRUE	0.009	0.294	0.265	1	0	cassette	0.265
71	ENSG000002	ENSG000002	ENSG000002	chr21	+	ENSG000002	ENSG000002	TRUE	cassette^126|alt3^121|a		C1_A	chr21:	FALSE	FALSE	TRUE	0	0.175	0.151	0.989	0.003	cassette	0.151

72	ENSG000002	ENSG000002	ENSG000002	chr21	+	ENSG000002	ENSG000002	TRUE	cassette^126|alt3^121|a		C1_A	chr21:	FALSE	FALSE	TRUE	0.004	0.341	0.307	0.996	0	cassette	0.307
73	ENSG000002	ENSG000002	ENSG000002	chr21	+	ENSG000002	ENSG000002	TRUE	cassette^126	TRUE	C1_A	chr21:83933	FALSE	FALSE	TRUE	0.006	0.268	0.252	1	0	cassette	0.252
74	ENSG000002	ENSG000002	ENSG000002	chr21	+	ENSG000002	ENSG000002	TRUE	cassette^126|alt3^121|a		C1_A	chr21:	FALSE	FALSE	TRUE	0.019	0.146	0.124	0.95	0.018	cassette	0.124
75	ENSG000002	ENSG000002	ENSG000002	chr21	+	ENSG000002	ENSG000002	TRUE	cassette^126|alt3^121|a		C1_A	chr21:	FALSE	TRUE	TRUE	0.032	0.253	0.214	1	0	cassette	0.214
76	ENSG000002	ENSG000002	ENSG000002	chr21	+	ENSG000002	ENSG000002	TRUE	cassette^126|alt3^121|a		C1_A	chr21:	FALSE	FALSE	TRUE	0.02	0.173	0.147	0.974	0.008	cassette	0.147
77	ENSG000002	ENSG000002	ENSG000002	chr21	+	ENSG000002	ENSG000002	TRUE	cassette^126|alt3^121|a		C1_A	chr21:	FALSE	FALSE	TRUE	0.028	0.255	0.219	1	0	cassette	0.219
78	ENSG000002	ENSG000002	ENSG000002	chr21	+	ENSG000002	ENSG000002	TRUE	cassette^126	TRUE	E1_E2_J1	chr21:83986	FALSE	FALSE	TRUE	0	0.186	0.16	1	0	cassette	0.16
79	ENSG000002	ENSG000002	ENSG000002	chr21	+	ENSG000002	ENSG000002	TRUE	cassette^126|alt3^121|a		C1_A	chr21:	FALSE	FALSE	TRUE	0.013	0.157	0.132	0.955	0.016	cassette	0.132
80	ENSG000002	ENSG000002	ENSG000002	chr21	+	ENSG000002	ENSG000002	TRUE	cassette^126|alt3^121|a		C1_A	chr21:	FALSE	FALSE	TRUE	0	0.147	0.123	0.951	0.02	cassette	0.123
81	ENSG000002	ENSG000002	ENSG000002	chr21	+	ENSG000002	ENSG000002	TRUE	cassette^126|alt3^121|a		C1_A	chr21:	FALSE	FALSE	TRUE	0	0.265	0.244	1	0	cassette	0.244
82	ENSG000002	ENSG000002	ENSG000002	chr21	+	ENSG000002	ENSG000002	TRUE	cassette^126|alt3^121|a		C1_A	chr21:	FALSE	FALSE	FALSE	0	0.119	0.096	0.913	0.046	cassette	0.096
83	ENSG000002	ENSG000002	ENSG000002	chr21	+	ENSG000002	ENSG000002	TRUE	cassette^126	TRUE	E1_E2_J2	chr21:83998	FALSE	FALSE	FALSE	0.009	0.148	0.121	0.932	0.024	cassette	0.121
84	ENSG000002	ENSG000002	ENSG000002	chr21	+	ENSG000002	ENSG000002	TRUE	cassette^126|alt3^121|a		C1_A	chr21:	FALSE	FALSE	TRUE	0.025	0.325	0.304	1	0	cassette	0.304
85	ENSG000002	ENSG000002	ENSG000002	chr21	+	ENSG000002	ENSG000002	TRUE	cassette^126|alt3^121|a		C1_A	chr21:	FALSE	FALSE	FALSE	0.016	0.227	0.192	0.932	0.01	cassette	0.192
86	ENSG000002	ENSG000002	ENSG000002	chr21	+	ENSG000002	ENSG000002	TRUE	cassette^126|alt3^121|a		C1_A	chr21:	FALSE	FALSE	FALSE	0.015	0.131	0.114	0.927	0.032	cassette	0.114
87	ENSG000002	ENSG000002	ENSG000002	chr21	+	ENSG000002	ENSG000002	TRUE	cassette^126|alt3^121|a		C1_A	chr21:	FALSE	FALSE	TRUE	0.034	0.19	0.163	0.982	0.004	cassette	0.163
88	ENSG000002	ENSG000002	ENSG000002	chr21	+	ENSG000002	ENSG000002	TRUE	cassette^126|alt3^121|a		C1_A	chr21:	FALSE	FALSE	TRUE	0.004	0.412	0.39	1	0	cassette	0.39
89	ENSG000002	ENSG000002	ENSG000002	chr21	+	ENSG000002	ENSG000002	TRUE	cassette^126|alt3^121|a		C1_A	chr21:	FALSE	FALSE	TRUE	0	0.212	0.186	0.997	0.001	cassette	0.186
90	ENSG000002	ENSG000002	ENSG000002	chr21	+	ENSG000002	ENSG000002	TRUE	cassette^126|alt3^121|a		C1_A	chr21:	FALSE	FALSE	TRUE	0.004	0.379	0.346	1	0	cassette	0.346
91	ENSG000002	ENSG000002	ENSG000002	chr21	+	ENSG000002	ENSG000002	TRUE	cassette^126|alt3^121|a		C1_A	chr21:	FALSE	FALSE	TRUE	0.001	0.182	0.173	0.964	0.008	cassette	0.173
92	ENSG000002	ENSG000002	ENSG000002	chr21	+	ENSG000002	ENSG000002	TRUE	cassette^126|alt3^121|a		C1_A	chr21:	FALSE	FALSE	TRUE	0.002	0.248	0.216	1	0	cassette	0.216
93	ENSG000002	ENSG000002	ENSG000002	chr21	+	ENSG000002	ENSG000002	TRUE	cassette^126|alt3^121|a		C1_A	chr21:	FALSE	FALSE	TRUE	0	0.167	0.147	0.974	0.008	cassette	0.147
94	ENSG000002	ENSG000002	ENSG000002	chr21	+	ENSG000002	ENSG000002	TRUE	cassette^126|alt3^121|a		C1_A	chr21:	FALSE	FALSE	TRUE	0	0.199	0.171	1	0	cassette	0.171
95	ENSG000002	ENSG000002	ENSG000002	chr21	+	ENSG000002	ENSG000002	TRUE	cassette^126|alt3^121|a		C1_A	chr21:	FALSE	TRUE	TRUE	0	0.422	0.399	1	0	cassette	0.399
96	ENSG000002	ENSG000002	ENSG000002	chr21	+	ENSG000002	ENSG000002	TRUE	cassette^126|alt3^121|a		C1_A	chr21:	FALSE	FALSE	TRUE	0.001	0.21	0.172	0.981	0.005	cassette	0.172
97	ENSG000002	ENSG000002	ENSG000002	chr21	+	ENSG000002	ENSG000002	TRUE	cassette^126|alt3^121|a		C1_A	chr21:	FALSE	FALSE	TRUE	0.001	0.196	0.164	0.978	0.005	cassette	0.164
98	ENSG000002	ENSG000002	ENSG000002	chr21	+	ENSG000002	ENSG000002	TRUE	cassette^126|alt3^121|a		C1_A	chr21:	FALSE	FALSE	TRUE	0	0.214	0.185	0.981	0.004	cassette	0.185
99	ENSG000002	ENSG000002	ENSG000002	chr21	+	ENSG000002	ENSG000002	TRUE	cassette^126|alt3^121|a		C1_A	chr21:	FALSE	FALSE	TRUE	0.001	0.22	0.192	0.999	0	cassette	0.192
100	ENSG000002	ENSG000002	ENSG000002	chr21	+	ENSG000002	ENSG000002	TRUE	cassette^126|alt3^121|a		C1_A	chr21:	FALSE	FALSE	TRUE	0.017	0.23	0.198	0.992	0.001	cassette	0.198
101	ENSG000002	ENSG000002	ENSG000002	chr21	+	ENSG000002	ENSG000002	TRUE	cassette^126|alt3^121|a		C1_A	chr21:	FALSE	FALSE	FALSE	0.001	0.137	0.116	0.913	0.037	cassette	0.116
102	ENSG000002	ENSG000002	ENSG000002	chr21	+	ENSG000002	ENSG000002	TRUE	cassette^126|alt3^121|a		C1_A	chr21:	FALSE	FALSE	TRUE	0	0.539	0.517	1	0	cassette	0.517
103	ENSG000002	ENSG000002	ENSG000002	chr21	+	ENSG000002	ENSG000002	TRUE	cassette^126	TRUE	Distal	chr21:84387	FALSE	FALSE	TRUE	0.024	0.172	0.139	0.955	0.014	AFE/ALE	0.139
104	ENSG000002	ENSG000002	ENSG000002	chr21	+	ENSG000002	ENSG000002	TRUE	cassette^126|alt3^121|a		C1_A	chr21:	FALSE	FALSE	TRUE	0.001	0.221	0.196	1	0	cassette	0.196
105	ENSG000002	ENSG000002	ENSG000002	chr21	+	ENSG000002	ENSG000002	TRUE	cassette^126|alt3^121|a		C1_A	chr21:	FALSE	FALSE	TRUE	0.026	0.18	0.147	0.974	0.007	cassette	0.147
106	ENSG000002	ENSG000002	ENSG000002	chr21	+	ENSG000002	ENSG000002	TRUE	cassette^126|alt3^121|a		C1_A	chr21:	FALSE	FALSE	TRUE	0.005	0.677	0.664	1	0	cassette	0.664
107	ENSG000002	ENSG000002	ENSG000002	chr21	+	ENSG000002	ENSG000002	TRUE	cassette^126	TRUE	Proximal	chr21:83900	FALSE	FALSE	FALSE	0.016	0.181	0.148	0.935	0.013	AFE/ALE	0.148
108	ENSG000002	ENSG000002	ENSG000002	chr21	+	ENSG000002	ENSG000002	TRUE	cassette^126	TRUE	Distal	chr21:84088	FALSE	FALSE	TRUE	0	0.146	0.121	0.978	0.009	AFE/ALE	0.121
109	ENSG000002	ENSG000002	ENSG000002	chr21	+	ENSG000002	ENSG000002	TRUE	cassette^126	TRUE	Distal	chr21:84244	FALSE	FALSE	TRUE	0.005	0.228	0.211	0.994	0.001	AFE/ALE	0.211
110	ENSG000002	ENSG000002	ENSG000002	chr21	+	ENSG000002	ENSG000002	TRUE	cassette^126	TRUE	Distal	chr21:83818	FALSE	FALSE	TRUE	0.001	0.173	0.153	0.966	0.01	AFE/ALE	0.153
111	ENSG000002	ENSG000002	ENSG000002	chr21	+	ENSG000002	ENSG000002	TRUE	cassette^126|alt3^121|a		A_C2	chr21:	FALSE	FALSE	TRUE	0.001	0.299	0.284	0.951	0.005	cassette	0.284
112	ENSG000002	ENSG000002	ENSG000002	chr21	+	ENSG000002	ENSG000002	TRUE	cassette^126	TRUE	E2_E1_J1	chr21:83807	FALSE	FALSE	TRUE	0	0.24	0.208	0.969	0.005	cassette	0.208
113	ENSG000002	ENSG000002	ENSG000002	chr21	+	ENSG000002	ENSG000002	TRUE	cassette^126	TRUE	Proximal	chr21:83876	FALSE	FALSE	FALSE	0.034	0.208	0.168	0.939	0.009	AFE/ALE	0.168
114	ENSG000002	ENSG000002	ENSG000002	chr21	+	ENSG000002	ENSG000002	TRUE	cassette^126	TRUE	C2_C1	chr21:83876	FALSE	FALSE	FALSE	0	0.126	-0.111	0.921	0.036	skipping	0.111
115	ENSG000002	ENSG000002	ENSG000002	chr21	+	ENSG000002	ENSG000002	TRUE	cassette^126|alt3^121|a		A_C2	chr21:	FALSE	FALSE	TRUE	0.021	0.21	0.171	0.975	0.005	cassette	0.171
116	ENSG000002	ENSG000002	ENSG000002	chr21	+	ENSG000002	ENSG000002	TRUE	cassette^126|alt3^121|a		A_C2	chr21:	FALSE	FALSE	TRUE	0.001	0.273	0.256	1	0	cassette	0.256
117	ENSG000002	ENSG000002	ENSG000002	chr21	+	ENSG000002	ENSG000002	TRUE	cassette^126|alt3^121|a		A_C2	chr21:	FALSE	FALSE	TRUE	0.001	0.203	0.171	0.995	0.001	cassette	0.171
118	ENSG000002	ENSG000002	ENSG000002	chr21	+	ENSG000002	ENSG000002	TRUE	cassette^126	TRUE	Distal	chr21:83929	FALSE	FALSE	FALSE	0	0.134	0.114	0.944	0.025	AFE/ALE	0.114
119	ENSG000002	ENSG000002	ENSG000002	chr21	+	ENSG000002	ENSG000002	TRUE	cassette^126	TRUE	Distal	chr21:83876	FALSE	FALSE	FALSE	0.001	0.148	0.125	0.939	0.022	AFE/ALE	0.125
120	ENSG000002	ENSG000002	ENSG000002	chr21	+	ENSG000002	ENSG000002	TRUE	cassette^126|alt3^121|a		A_C2	chr21:	FALSE	FALSE	TRUE	0	0.203	0.187	1	0	cassette	0.187
121	ENSG000002	ENSG000002	ENSG000002	chr21	+	ENSG000002	ENSG000002	TRUE	cassette^126|alt3^121|a		A_C2	chr21:	FALSE	FALSE	TRUE	0	0.212	0.206	0.984	0.003	cassette	0.206
122	ENSG000002	ENSG000002	ENSG000002	chr21	+	ENSG000002	ENSG000002	TRUE	cassette^126	TRUE	Distal	chr21:83899	FALSE	FALSE	TRUE	0.019	0.195	0.167	0.987	0.003	AFE/ALE	0.167
123	ENSG000002	ENSG000002	ENSG000002	chr21	+	ENSG000002	ENSG000002	TRUE	cassette^126|alt3^121|a		A_C2	chr21:	FALSE	FALSE	TRUE	0.036	0.171	0.151	0.999	0	cassette	0.151
124	ENSG000002	ENSG000002	ENSG000002	chr21	+	ENSG000002	ENSG000002	TRUE	cassette^126|alt3^121|a		A_C2	chr21:	FALSE	FALSE	TRUE	0.001	0.287	0.26	1	0	cassette	0.26
125	ENSG000002	ENSG000002	ENSG000002	chr21	+	ENSG000002	ENSG000002	TRUE	cassette^126|alt3^121|a		A_C2	chr21:	FALSE	FALSE	TRUE	0.013	0.237	0.196	0.966	0.004	cassette	0.196
126	ENSG000002	ENSG000002	ENSG000002	chr21	+	ENSG000002	ENSG000002	TRUE	cassette^126|alt3^121|a		A_C2	chr21:	FALSE	FALSE	FALSE	0.003	0.157	0.128	0.932	0.025	cassette	0.128
127	ENSG000002	ENSG000002	ENSG000002	chr21	+	ENSG000002	ENSG000002	TRUE	cassette^126|alt3^121|a		A_C2	chr21:	FALSE	FALSE	TRUE	0.001	0.185	0.159	0.993	0.002	cassette	0.159
128	ENSG000002	ENSG000002	ENSG000002	chr21	+	ENSG000002	ENSG000002	TRUE	cassette^126|alt3^121|a		A_C2	chr21:	FALSE	FALSE	TRUE	0.024	0.279	0.259	1	0	cassette	0.259
129	ENSG000002	ENSG000002	ENSG000002	chr21	+	ENSG000002	ENSG000002	TRUE	cassette^126|alt3^121|a		A_C2	chr21:	FALSE	FALSE	TRUE	0.002	0.252	0.224	1	0	cassette	0.224
130	ENSG000002	ENSG000002	ENSG000002	chr21	+	ENSG000002	ENSG000002	TRUE	cassette^126|alt3^121|a		A_C2	chr21:	FALSE	FALSE	FALSE	0	0.137	0.123	0.933	0.018	cassette	0.123
131	ENSG000002	ENSG000002	ENSG000002	chr21	+	ENSG000002	ENSG000002	TRUE	cassette^126|alt3^121|a		A_C2	chr21:	FALSE	FALSE	FALSE	0.027	0.178	0.135	0.913	0.021	cassette	0.135
132	ENSG000002	ENSG000002	ENSG000002	chr21	+	ENSG000002	ENSG000002	TRUE	cassette^126|alt3^121|a		A_C2	chr21:	FALSE	FALSE	TRUE	0.034	0.665	0.634	1	0	cassette	0.634
133	ENSG000002	ENSG000002	ENSG000002	chr21	+	ENSG000002	ENSG000002	TRUE	cassette^126|alt3^121|a		A_C2	chr21:	FALSE	FALSE	TRUE	0.003	0.184	0.157	0.994	0.002	cassette	0.157
134	ENSG000002	ENSG000002	ENSG000002	chr21	+	ENSG000002	ENSG000002	TRUE	cassette^126|alt3^121|a		A_C2	chr21:	FALSE	FALSE	TRUE	0.038	0.278	0.253	0.994	0.001	cassette	0.253
135	ENSG000002	ENSG000002	ENSG000002	chr21	+	ENSG000002	ENSG000002	TRUE	cassette^126|alt3^121|a		A_C2	chr21:	FALSE	FALSE	TRUE	0.013	0.148	0.123	1	0	cassette	0.123
136	ENSG000002	ENSG000002	ENSG000002	chr21	+	ENSG000002	ENSG000002	TRUE	cassette^126|alt3^121|a		A_C2	chr21:	FALSE	FALSE	TRUE	0.041	0.305	0.262	1	0	cassette	0.262
137	ENSG000002	ENSG000002	ENSG000002	chr21	+	ENSG000002	ENSG000002	TRUE	cassette^126|alt3^121|a		A_C2	chr21:	FALSE	FALSE	TRUE	0.048	0.519	0.395	0.991	0	cassette	0.395
138	ENSG000002	ENSG000002	ENSG000002	chr21	+	ENSG000002	ENSG000002	TRUE	cassette^126	TRUE	C2_A	chr21:84421	FALSE	FALSE	TRUE	0.033	0.185	0.146	0.97	0.009	cassette	0.146
139	ENSG000002	ENSG000002	ENSG000002	chr21	+	ENSG000002	ENSG000002	TRUE	cassette^126|alt3^121|a		A_C2	chr21:	FALSE	FALSE	TRUE	0.029	0.127	0.102	0.972	0.014	cassette	0.102
140	ENSG000002	ENSG000002	ENSG000002	chr21	+	ENSG000002	ENSG000002	TRUE	cassette^126|alt3^121|a		A_C2	chr21:	FALSE	FALSE	TRUE	0	0.169	0.147	1	0	cassette	0.147
141	ENSG000002	ENSG000002	ENSG000002	chr21	+	ENSG000002	ENSG000002	TRUE	cassette^126	TRUE	Proximal	chr21:84331	FALSE	FALSE	TRUE	0	0.393	0.366	1	0	AFE/ALE	0.366
142	ENSG000002	ENSG000002	ENSG000002	chr21	+	ENSG000002	ENSG000002	TRUE	cassette^126|alt3^121|a		A_C2	chr21:	FALSE	FALSE	FALSE	0.002	0.155	0.124	0.936	0.025	cassette	0.124
143	ENSG000002	ENSG000002	ENSG000002	chr21	+	ENSG000002	ENSG000002	TRUE	cassette^126|alt3^121|a		A_C2	chr21:	FALSE	FALSE	TRUE	0	0.213	0.187	0.971	0.005	cassette	0.187

144	ENSG000002	ENSG000002	ENSG000002	chr21	+	ENSG000002	ENSG000002	TRUE	cassette^126|alt3^121|a		A_C2	chr21:	FALSE	FALSE	TRUE	0.008	0.31	0.292	0.997	0	cassette	0.292
145	ENSG000002	ENSG000002	ENSG000002	chr21	+	ENSG000002	ENSG000002	TRUE	cassette^126	TRUE	C2_A1	chr21:840278	FALSE	FALSE	TRUE	0.001	0.165	0.142	1	0	cassette	0.142
146	ENSG000002	ENSG000002	ENSG000002	chr14	-	ENSG000002	ENSG000002	TRUE	alt3^5|alt5^	TRUE	E1_E2_J2	chr14:498627	FALSE	FALSE	TRUE	0	0.164	0.138	0.981	0.007	cassette	0.138
147	ENSG000002	ENSG000002	ENSG000002	chr3	+	ENSG000002	ENSG000002	TRUE	orphan_junc	TRUE	Orphan	chr3:134783	FALSE	FALSE	FALSE	0.046	0.186	0.139	0.944	0.016	cassette	0.139
148	ENSG000002	ENSG000002	ENSG000002	chr3	+	ENSG000002	ENSG000002	TRUE	orphan_junc	TRUE	Orphan	chr3:134783	FALSE	FALSE	TRUE	0.014	0.528	0.514	1	0	cassette	0.514
